# Evaluation of energy, carbon dioxide, and air emission implications of medium- and heavy-duty truck electrification in the United States using EPA’s regional TIMES energy systems model

**DOI:** 10.1088/2753-3751/ad958b

**Published:** 2025-01-07

**Authors:** Andrew Zalesak, Noah Kittner, Daniel H Loughlin, Pervin Ozge Kaplan

**Affiliations:** 1 ORISE Research Fellow (Former), U.S. Environmental Protection Agency, Durham, NC 27711, United States of America; 2 University of North Carolina at Chapel Hill, Chapel Hill, NC 27599-7400, United States of America; 3 U.S. Environmental Protection Agency, Office of Research and Development, Durham, NC 27709, United States of America

**Keywords:** energy system analysis, carbon tax, heavy-duty trucks, electrification, decarbonizing transport, netzero, TIMES model

## Abstract

Electrifying on-road trucking is a strategy for decarbonizing the transportation sector. While battery-electric trucks have zero tailpipe emissions, the associated increase in electric sector grid emissions would offset a portion of on-road emission reductions. We utilize a techno-economic energy systems optimization model to assess the system-wide carbon dioxide (CO_2_) and air pollutant emission implications of medium- and heavy-duty truck electrification in the United States of America. Results suggest that by transitioning to 100% battery-electric medium- and heavy-duty vehicles (MDVs and HDVs, together MHDVs), sales by 2050 would result in net CO_2_ emission benefits should the electric sector decarbonize simultaneously. Combining a tax of $100 per ton of CO_2_, increasing at 5% per year, with electrification targets would yield a net 52% reduction in energy system CO_2_ emissions in 2050. Across regions, the transportation sector nitrogen oxide (NO_*X*_*)* emissions—an ozone precursor—further decrease by 9%–26% compared to the ‘business-as-usual’ (BAU) level in 2050. The level of emission reductions and the extent of transportation decarbonization are driven by vehicle cost and performance projections along with underlying assumptions on the time of charging.

## 1. Introduction

The Paris Agreement set a 2100 target of global mean temperatures of no more than 1.5 °C above pre-industrial levels (UNFCCC 2015). However, the world is currently on a trajectory to reach 1.5 °C warming over the next two decades unless governments take immediate, large-scale action to reduce greenhouse gas (GHG) emissions (IPCC 2021). In 2019, the United States of America contributed 6560 million metric tons of carbon dioxide (CO_2_)-equivalents to the ocean and atmosphere, and the transportation sector alone accounted for 29% of this total (U.S. Environmental Protection Agency 2022a). The transportation sector also contributes to air pollution impacting human health and the environment. Nitrogen oxide (NO_*X*_) emissions contribute to regional and local air pollution, causing as many as 20 000 premature deaths per year in the United States of America in recent years (Choma et al 2021, U.S. Environmental Protection Agency (U.S. EPA) 2022b). Furthermore, vehicle exhaust and brake and tire wear produce fine particulate matter (PM), which is responsible for health impacts such as impaired lung function, asthma, and increased risk of heart failure. Nearly 20% of the population in the United States of America lives near roadways, and minority and low-income households are over-represented in this statistic (Rowangould 2013, Mukherjee et al 2020). Along with mitigating local air pollution, transportation electrification could potentially reduce GHGs. Thus, transitioning the on-road fleet away from internal combustion engine vehicles is a crucial step in confronting the dual challenges of climate change and air pollution.

Electrifying on-road vehicles has been proposed as an effective way to decarbonize the transportation sector, particularly for light-duty passenger vehicles (Wu et al 2012, Muratori et al 2017, Isik et al 2021, Liimatainen 2021, Yuan et al 2021). For heavy-duty vehicles, previous work has suggested pathways such as hydrogen or biofuels may be more economically viable than electrification (Bahn et al 2013, Zhao et al 2013, Çabukoglu et al 2018, Lajevardi et al 2019, Gross 2020). One reason for this is that batteries capable of providing enough power and range for heavy-duty trucks have historically been large and heavy, limiting vehicle payload capacities. It remains unclear how much this will change as the technology develops; battery weight may continue to be a barrier to adoption in many vehicle segments. Additional barriers include the lack of rapid charging infrastructure on major highways and the high capital costs relative to conventional technologies (Cabukoglu et al 2018, Phadke et al 2021). Battery costs have fallen by nearly 90% since 2010, reaching a global average of $132 kWh^−1^ for light-duty vehicle (LDV) applications in 2021 (BNEF 2021). In the last two years, at least four comprehensive total cost of ownership studies have found battery-electric heavy-duty long-haul trucks can be less expensive than their diesel counterparts under specific conditions (Hunter et al 2021, Phadke et al 2021, Ledna et al 2022, Noll et al 2022). Battery costs are now estimated to drop to as low as $60 kWh^−1^ by 2030 for LDV application (Bloomberg NEF 2021), increasing the prospects for decarbonization via vehicle electrification. The advancements in technology could spill over to the medium- and heavy-duty vehicle (MDV and HDV, together MHDV) sectors. Policies could speed this transition, such as subsidies for fast-charging infrastructure, electricity pricing that allows truck operators to take advantage of cheaper, off-peak electricity when they charge, and incentives for reuse or recycling of batteries at the end of vehicles’ useful on-road lives (Harper et al 2019, Phadke et al 2021). Vehicle electrification, whether in the LDV, MDV, or HDV sectors, is expected to have a significant impact on the electric grid. For example, electrification of on-road transport may raise the electricity demand in the United States of America by as much as 38% in a high-electric vehicle (EV)-penetration scenario (Mai et al 2018, EIA 2020). Thus, increases in CO_2_ and air pollutant emissions from the electric sector can offset a portion of transportation sector reductions. However, trends in the electric sector are leading to a reduced role for fossil fuels. Electricity generation from coal declined by 39% in the United States of America between 2012 and 2021, and substantial cost reductions are leading to growing shares for renewables like solar and wind. Policies can speed this transition away from fossil fuels (Dubin 2021, EIA 2022b), including carbon taxes, cap-and-trade systems, feed-in tariffs, and quotas (Rockstrom et al 2017) as well as tax incentives, rebate programs and grants provided by federal government (through the Inflation Reduction Act 2022, Bipartisan Infrastructure Law 2021).

In this study, the CO_2_ and criteria air pollutant (CAP) emissions reduction potential of two measures in combination are assessed: (1) a target of 100% electrification of MDVs and HDVs by 2050 (zero-emission vehicles (ZEVs) target), and (2) a hypothetical national, economy-wide CO_2_ tax. The goal is to present air emission co-benefits of increased electrification in MHDVs. Using The Integrated MARKAL-EFOM system (TIMES)—a bottom-up, technology-rich energy systems optimization model—the cross-sector air and GHG emission impacts of the above-mentioned measures are examined, both of which are often overlooked in decarbonization studies. TIMES allows the consideration of alternative transport modes, such as buses and short- and long-haul trucking, the time required for stock turnover, how regulations reduce air pollutant emissions from future conventional truck vintages, impacts on fuel prices, and how these price changes could drive fuel switching in other sectors. We utilized and modified the U.S. EPA’s 9 Region TIMES database where we simulated alternative technology cost and performance scenarios along with representative charging times assumptions. By layering on a carbon tax, this study design allows us to explore the complimentary roles of pricing carbon versus technology mandates for electrification.

### 1.1. Background

Many studies have recognized the potential tradeoffs between tailpipe emissions and electricity generation emissions from electric vehicles, mostly focusing on LDVs. Studies have explored the role of a local energy mix through case studies of various regions around the world, including the United States of America or regions within it (Thomas 2012, Lee and Thomas 2017, Sen et al 2017, Spangher et al 2019, Isik et al 2021, Ou et al 2021, Raju et al 2021, Tong et al 2021, Wolfram et al 2021); individual provinces in Canada (Talebian et al 2018, Keller et al 2019, Lajevardi et al 2019, Doluweera et al 2020); Europe (Schill and Gerbaulet 2015, Plotz et al 2019), and all or part of China (Wu et al 2012). These studies differ in their assumptions about EV penetration, i.e. whether to include or exclude hybrid vehicles under the umbrella of vehicle electrification, whether they consider the electrification of LDVs, MDVs or HDVs in their analyses, and whether they consider GHGs, CAPs, or both. Research on lifecycle impacts of electric vehicles is still developing and becoming more conclusive. Muratori et al (2021) provided a comprehensive review of literature, exploring multiple facets of electric vehicles and still concluded that in the majority of cases, life-cycle GHG emissions from electric vehicles will have lower GHGs compared to their conventional counterparts (Muratori et al 2021). Modeling techniques differ also, ranging from life-cycle assessment (Thomas 2012, Wu et al 2012, Lee and Thomas 2017, Sen et al 2017, Talebian et al 2018, Raju et al 2021), to simulation (Lajevardi et al 2019, Spangher et al 2019, Doluweera et al 2020), and energy system modeling (Schill and Gerbaulet 2015, Keller et al 2019, Plötz et al 2019, Isik et al 2021, Ou et al 2021, Tong et al 2021, Wolfram et al 2021, Yuan et al 2021).

The early literature on freight truck electrification focused mostly on plug-in hybrid technology as the viable form of electrification for long-haul trucks, as in Bahn et al (2013). As battery costs have fallen, battery-electric technology has gained an increased presence in the research space. Of the studies that examine both GHGs and air pollutants, Ou et al (2021) found that high levels of LDV electrification reduced CO_2_ and NO_*X*_ across the United States of America. Isik et al (2021) similarly found that for New York City, investing in fuel-efficiency and electric vehicle technologies in the near term has benefits even under a carbon-intensive grid. Additionally, Tong et al (2021) suggested that electric heavy-duty vehicle adoption will have net health benefits under a business-as-usual grid only in the West Coast region, whereas it will have net health benefits in all regions under an 80% renewables grid.

Several of these studies are directly relevant to the present study. Ou et al (2021) used the multi-sector human-Earth systems model GCAM-USA to assess CO_2_ reductions due to LDV electrification under various electric sector decarbonization scenarios, whereas Isik et al (2021) used the bottom-up energy system optimization model—COMET-NYC—to evaluate the impact on electric-sector decarbonization on meeting New York City’s 80 × 50 emissions reductions goals through LDV electrification. Ou et al (2021) found net CO_2_, NO_*X*_, and SO_2_ reductions from electrification, even under a scenario where all new electric generation capacity is fossil fuel. While they showed the potential for increases in PM_2.5_ emissions, these increases were small relative to the total emissions and in relation to expected reductions by 2050. Isik et al (2021) found that investing in fuel-efficiency and electric vehicle technologies in the near term has benefits even under a carbon-intensive grid. In addition, Isik et al (2021) focused on urban areas, where electric vehicle technologies in the scenarios reduce urban CO_2_ and NO_*X*_ emissions. Additionally, Tong et al (2021) used an integrated assessment approach to model health damages from electric heavy-duty vehicle adoption in the United States. Their analysis suggested that electric heavy-duty vehicle adoption will have net health benefits under a business-as-usual grid only in the West Coast region, whereas it will have net health benefits in all regions under an 80% renewables grid. Other analyses have used TIMES to examine the role of carbon taxes on decarbonization and found limited opportunities for the transportation sector to reduce emissions, i.e. Roth et al (2020), even though they did not characterize MDVs and HDVs, i.e. Jordan et al (2023). More recently, Aryanpur and Rogan (2024) demonstrate how prioritizing heavy truck electrification creates new tipping points that could lead to dramatically greater emission reductions in the transportation sector due to the electrification or use of alternative fuels for MDVs and HDVs. This opens an opportunity to utilize TIMES to understand how electrification affects emissions but expands the use of previously uncharacterized transport representation in the medium and heavy-duty sectors, thus providing new insights not generated by other similar types of studies. Additionally, other work suggests that not only does freight electrification lead to emission reductions, but also that the underlying charging profiles and fleet management influence the extent to which emission reductions are realized (Lu et al 2023). Research on how the charging profiles for each transport mode will influence power system operations and the resultant emission reduction is an evolving subject. Muratori et al (2021) highlight the role of understanding load profiles in the context of increased transportation electrification and inherent uncertainty in the resultant load shapes. Tong et al (2021) estimate energy consumption, and location specific hourly charging load for long-haul electric trucks using a combination of vehicle energy modeling, truck traffic data and alternative charging and driving behaviors. Similarly, McNeil et al (2024) look at the evolution of electric grid and long-haul truck electrification issues from an analysis of highway corridors and when it is most advantageous from a health and climate standpoint.

The present study advances this literature by assessing net emissions benefits from electrifying the medium- and heavy-duty transport sectors, including investigating the effects of including an energy system-wide CO_2_ tax. This study is the first energy system-level study of which we are aware that considers the electrification of large portions of MHDV fleets, not just by focusing on long-haul freight in the United States of America, but also considering potential price-induced fuel switching in other sectors.

## 2. Methods

### 2.1. Times

The TIMES energy system optimization framework is used in this analysis. TIMES can be used to explore complex economic interactions within energy systems at local, regional, national or global scales. The model can represent every stage of energy transformation from the extraction of energy resources to end-use demand, including trade, and chooses between competing technologies to maximize the net total surplus of the system (Loulou et al 2016). The user specifies end-use demands for each sector, the existing energy technology stock, and estimates of current and future costs and efficiencies of technologies for each modeled region. TIMES then simulates the evolution of the energy system over the coming decades, seeking to meet growing energy demands at least in cost by selecting the technologies and fuels that minimize the net present value of energy system expenditures. In performing this optimization, the linear programming-based optimization routine used by TIMES also considers constraints, such as minimum market share for a particular technology, or policies such as taxes or caps on pollutants (see Note 1 in the supplementary information for more details on TIMES and EPAUS9rT).

**Figure 1.**
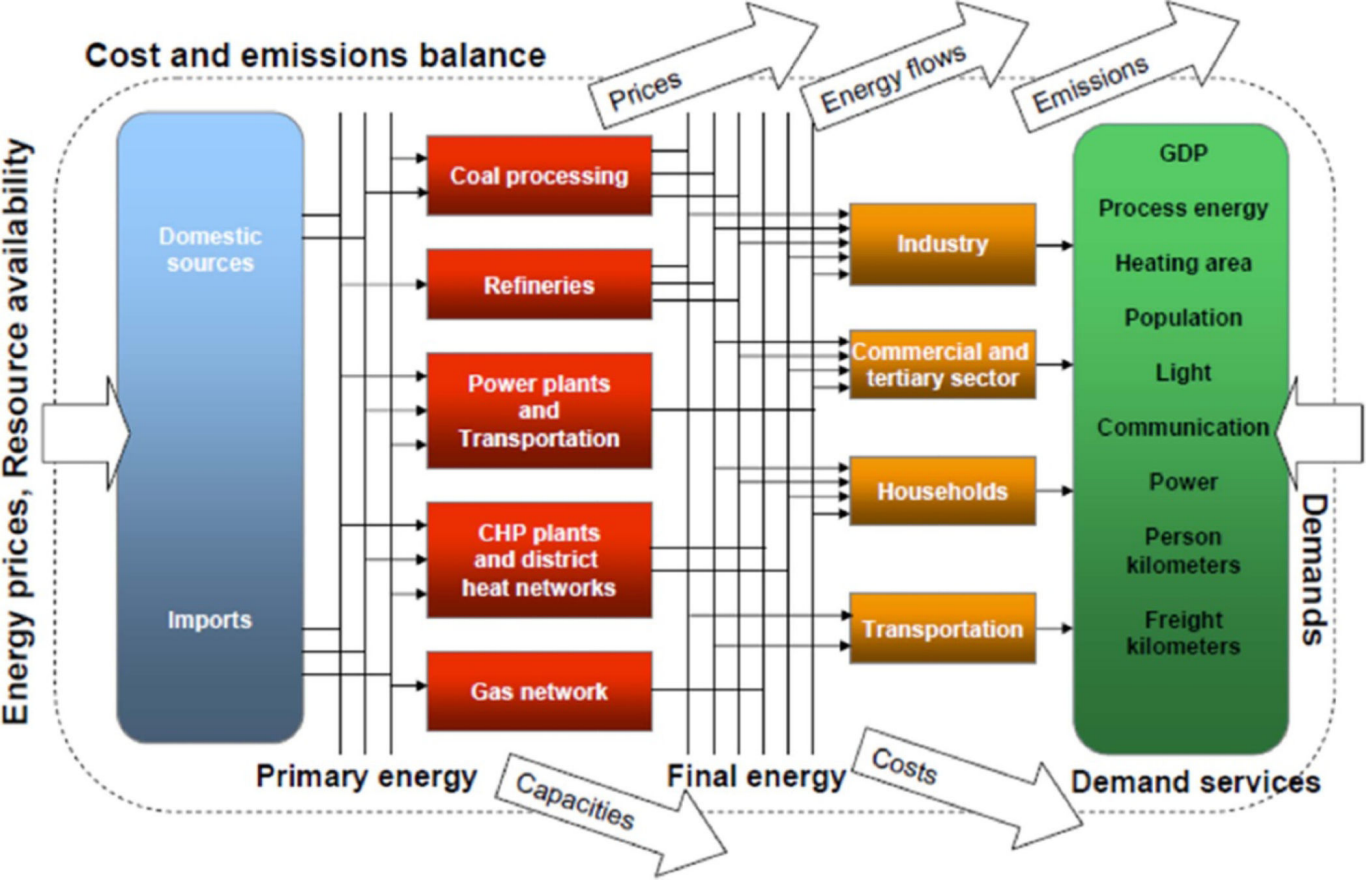
Reference energy system in EPAUS9rT.

For this study, we use the U.S. Environmental Protection Agency (EPA)’s publicly maintained nine-region database, the EPAUS9rT database, version 20.4, as the source of data for TIMES. The U.S. EPA’s Office of Research and Development maintains this publicly available database, which represents the entire U.S. energy system in nine regions (corresponding to the U.S. Census divisions) (Lenox et al 2013, Lenox 2022, U.S. Census Bureau 2022 and supplementary data note 1). The database includes: supply curves covering cost and emissions associated with the extraction and processing of primary energy such as coal, natural gas, crude oil, biomass feedstocks, and other non-biomass renewable resources; energy conversion technologies (e.g. refineries, electric generating units); end-use demand technologies (e.g. process heaters to meet industrial demand, furnaces for space heating demand in buildings, LDVs for transportation demand), and end-use demands in residential, commercial, industrial and transportation sectors (e.g. vehicle miles of travel, lumens of lighting, and value of shipments for the industrial sector). Technologies are specified by their cost (e.g. capital, operation, and maintenance (O&M)) and performance characteristics (capacity, efficiency, availability, and emission rates for CAPs and GHGs). EPAUS9rT represents all the electricity generation units in the U.S. including peaking fossil-fuel based generators and distributed energy resources (i.e. roof-top solar photovoltaic, and combined heat and power). We utilized generator-level power sector data collected and published (form EIA-860) by the U.S. Department of Energy which reports operational electric sector capacity in 2010 and 2015. Fossil fuel generation includes all the pollution control equipment as required by regulations. Renewable energy resources such as wind (both onshore and offshore) and solar representation are at regional level and account for the geographic and economic variability of the resource, including regionally varied parameters for wind class, and cost class. Capacity factors vary by time of day, season, technology, and region. The database includes capacity bounds for each type of renewable energy source based on technical, temporal and geographical feasibility. Curtailment of renewables is not considered because the model builds only the capacity needed to meet demand based on the resource availability across regions and time slices. The load duration curves are represented in 16 time-slices that facilitate balancing of load and end-use demand in building, transportation and industrial sectors. The time-slices are combinations of the four seasons and the time periods: day-AM, day-PM, night, and peak. The number of time-slice choices is a matter of balancing the representation of the energy system as close to reality to the computational requirements of the optimization model. An increased number of time slices would provide a better representation of the power sector and enable a better analysis of the transportation sector (Prina et al 2020). The goal of time slice representation via of seasons/intraday time slices is to better capture the fluctuations in load (instantaneous demand). This allows the alternative generating technologies to compete more directly without the need to impose user constraints, instead relying to a greater extent on their relative underlying capital costs, operating costs, and restrictions on startup/shut down. Due to it being a regional model, the 16 time-slice option provided us the best compromise in terms of computational aspects and yields results that are representative of the historical operations of the competing technologies. The supplementary information includes time-slice assumptions and load shapes for end-use service demands in buildings, industry, and transportation. The energy system is captured through a reference energy system, as depicted in figure 1.

The database incorporates several current energy and environmental policies, standards, and regulations (U.S. Environmental Protection Agency 2010a, 2014, 2010b, 2012, 2014a, 2016). The business-as-usual case is calibrated to the U.S. Energy Information Administration’s (EIA) Annual Energy Outlook (AEO) 2020, and the 2015 and 2020 time periods in the model reflect the actual fuel use, demands, and electricity generation. For 2025 through to 2050, technology shares and fuel use are simulated by the model, and ‘business-as-usual’ (BAU) trends follow AEO 2020 projections out into 2050. While TIMES EPAUS9rT includes representations of many technologies, it also serves as a starting point to which users can add, subtract, and update technology and policy portfolios to analyze their own technologies or modify technology representations to reflect alternative scenarios. In the following subsections, modifications that were made for this study are presented.

#### 2.1.1. Updates to EPAUS9rT’s transportation sector

**Figure 2.**
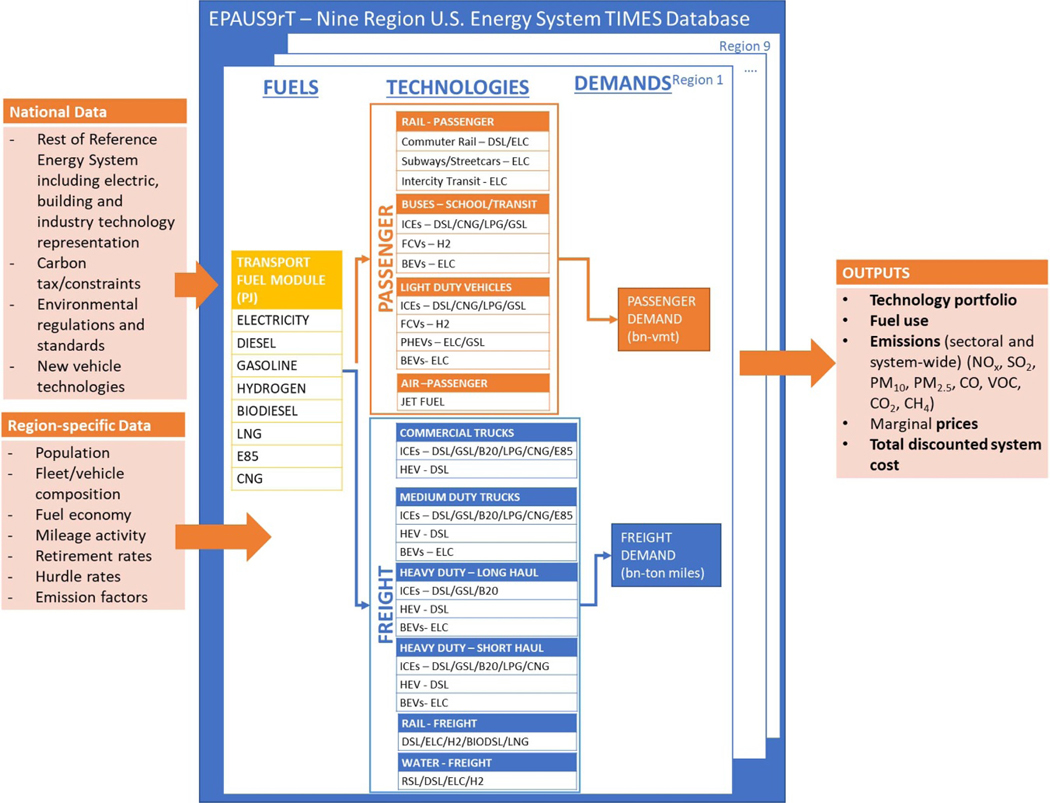
Transportation sector detail in EPAUS9rT. (Fuels: Electricity (ELC); Diesel (DSL); Gasoline (GSL); Hydrogen (H2); Biodiesel (B20); Liquified Natural Gas (LNG); Compressed Natural Gas (CNG) | Technologies: Internal Combustion Engine (ICE); Fuel Cell Vehicle (FCV); Plug-in Hybrid Electric Vehicle (PHEV); Battery Electric Vehicle (BEV); Hybrid Electric Vehicle (HEV))

One key input to the EPAUS9rT is the passenger ((LDV), rail, bus and air) and freight (commercial, medium-duty, heavy-duty trucks, rail and shipping) transport demand projections. These projections are taken from AEO, and regional distinctions across transport modes are incorporated. The transport modes are further refined to capture nuances between drive cycles, cost and performance. For instance, we split heavy-duty trucking to represent short-haul and long-haul modes using state-level data on freight shipments. To make a single delivery, long-haul truckers drive for more than 250 miles, however, the distance covered by short-haul trucking is an approximate 150–250 mile radius on a maximum. Long-haul trucking requires larger vehicles like tractor-trailers to carry huge loads of goods. These improvements enabled us to capture major highway corridors in the regional model. Similarly, we split buses into school and transit. Demand projections along with other assumptions on transportation modeling are provided in the supplementary information. Technology and fuel combination coverage for each transportation demand are illustrated in figure 2.

For this study, the LDV and MHDV fleet cost and performance characterization data in EPAUS9rT are updated using data from the National Renewable Energy Laboratory’s Transportation Annual Technology Baseline (NREL ATB) (NREL 2023). The dataset includes rich characterization per class size and fuel, as well as future year projections including characterization for electric and hydrogen fuels. Although the dataset included performance data for hydrogen vehicles, the EPAUS9rT v20.4 does not have full supply chain representation of hydrogen production. Therefore, hydrogen vehicles are excluded from our analysis. The supplementary information includes input data assumptions for the cost and efficiency assumptions for MHDV fleets.

Using these inputs, the TIMES framework finds the most cost-effective technology portfolio (fuel and vehicle type) to meet the transport demands. The model calculates the total cost of ownership for all vehicles through discounting capital costs over the lifetime of the vehicles in annual installments. Combined with annual operating (including fuel expenditures) and maintenance costs, the model calculates the full cost of ownership of the vehicles while determining which technology to utilize to meet the demand.

The TIMES framework can incorporate temporal end-use load shapes per transport mode, such that electric vehicle charging times would be represented. For heavy duty long-haul freight demand, the end-use load duration curves were based on data and insights gathered from figure 5 of Tong et al (2021). According to the study, the charging load profile for electric trucks is likely to peak in the middle of the day, though the exact timing varies across electric grid regions. The most striking observation was the occurrence of a very low charging load from 2 PM–1 AM and midnight–5 AM. Based on this information, we reduced the charging required during night hours significantly. In addition, a study conducted by Lawrence Berkeley National Laboratory (LBNL) (2021) for electric vehicle charging infrastructure assessment (AB-2127) provided load curves for other transport modes. The resultant load shapes per transport mode used in the model are provided in table S10 in the supplementary information.

Another key distinction of this study is the utilization of state-level air and GHG emission factors (EFs) for the transportation sector. EPAUS9rT included emissions factors derived from an older version of MOVES (MOVES 2014). MOVES is an emissions modeling system that estimates GHGs, CAPs, and other air toxic emissions from on-road vehicles such as cars, trucks and buses by considering federal emissions standards, fuels, temperatures, humidity, and emission control activities at the county to national scales (U.S. Environmental Protection Agency 2021). The EPAUS9rT included EFs where MOVES 2014 was simulated at national level, and these EFs generated from MOVES 2014 applied to all regions uniformly. Due to variations in drive cycles, temperature and humidity, as well as the age of the fleets, one expects variations in EFs for long-haul and short-haul trucking across the nation. To remedy this, we updated the transportation EFs in the EPAUS9rT by post-processing EFs after simulations conducted by the U.S. EPA’s MOVES version 3.0 (MOVES3). MOVES3 includes updated emission estimation methodologies and the impacts of regulations affecting trucks that were enacted after MOVES 2014 was developed, such as the Heavy-Duty Phase 2 GHG rule (U.S. Environmental Protection Agency 2022c). The MOVES3 simulations are conducted at state-level. In terms of air emissions, one advantage of battery-electric vehicles is the absence of an exhaust. Brake-wear emissions for EVs are expected to be lower than those for ICE vehicles due to the regenerative braking system of BEVs. Tire and road emissions are expected to be higher for BEVs due to their increased weight. Kriit et al (2021) assumes 50% more tire wear emissions and 30% less brake-wear emissions from BEVs compared to ICEs, whereas Liu et al (2022) assumes the same amount of tire and brake wear emissions from BEVs as ICEs. Currently, there are limited measurement data available on non-exhaust emissions from battery-electric vehicles due to tire-, brake- and road-wear. Therefore, we assumed electric vehicle PM_2.5_ emissions are equal to the tire wear and brake wear components of diesel vehicle PM_2.5_ emissions. State-level EFs generated from MOVES3 are post-processed and included in the EPAUS9rT v20.4. Note 2 detailed in the supplementary information includes additional details on EF development and key updates to the transportation sector updates to the EPAUS9rT. Note that the availability of chargers fast enough to replenish the large batteries used by heavy-duty trucks is an essential part of the long-haul truck electrification prevalence (Phadke et al 2021). We assumed that a sufficient fast-charging infrastructure will be made available in the future so that normal heavy-duty freight operations are not impeded. This study did not consider the role of factors such as the time-cost of charging, the potential cost of reduced payload capacity, or the cost of a charging infrastructure. Phadke et al (2021) included an additional upcharge for the electricity prices in their total cost of ownership which is around $0.03 kWh^−1^. Jordan et al (2023) and Aryanpur and Rogan (2024) included the costs of charging time, a reduced payload capacity and capital expenditures related to infrastructure build. They found that designing policies targeting and relieving the operational costs are more beneficial than incentivizing the capital cost of equipment. In our analysis, to capture these externalities, we utilized technology at specific larger discount rates for the early modeling years (applied to BEV trucks) that would result in a higher discounted present value compared to conventional options.

### 2.2. Scenario design

We evaluated the emission impacts of increasing the sales shares of ZEVs to 100% by 2050, both with and without a national, energy system-level carbon tax. In the BAU case, no carbon tax or ZEV target were applied. The ‘ZEV’ scenario featured a ZEV target and did not include the tax, while the ‘TAX’ scenario featured the tax without the ZEV target. The ‘ZEV + TAX’ scenarios featured both.

The ZEV target is patterned after the ‘Multi-state medium- and heavy-duty zero emission vehicle memorandum of understanding’, in which 16 states and the District of Columbia have agreed to reach a target of zero emissions from these vehicles by 2050 (ZEV Task Force 2022, 2020, 2018). For our scenarios, we assume this target is adopted nationally and met with battery-electric MHDVs. The ZEV target applies to new MHDV sales: battery-electric buses and trucks were constrained to comprise 30% of total new bus and truck capacity in 2030, increasing linearly to 100% in 2050. Since these vehicles have lifetimes between 10 and 20 years, this mandate did not guarantee that diesel technology will be cleared from the fleet by the end of the model period (2010–2050), only that the new capacity will be entirely electric.

The carbon tax was applied across the entire energy system, and thus influenced technology and fuel choices across all modeled sectors. The tax started at $100 ton^−1^ CO_2_ in 2025, rising linearly to $340 ton^−1^ CO_2_ in 2050 (in 2020 dollars). This starting point was approximately the carbon tax level implemented in Switzerland and Liechtenstein in 2020, which were close to level in Sweden ($119 per ton CO_2_eq in 2020) (World Bank Group 2020).

The scenarios use the mid-vehicle scenario cost and performance data from the NREL ATB. A set of sensitivity scenarios are simulated using the advanced-vehicle scenario from the NREL ATB where the cost and performance of fuel and technology combinations for fleets is improving at a much faster rate than the mid-vehicle scenario.

### 2.3. Constraints

A variety of constraints were used to enhance the behavioral realism of the model. Growth constraints set bounds on technology capacities to prevent the model from suddenly switching the entire fleet from one fuel type to another when it became cheaper to do so (the ‘winner-takes-all’ behavior of least-cost optimization models). In the LDV and HDV sectors, growth constraints were in place that set lower bounds on gasoline or diesel technologies, and these constraints were gradually relaxed to allow for fuel switching to occur across the modeled time horizon. To allow for the rapid electrification of fleets, constraints on fuel shares and technology growth rates were removed. A constraint was added to the school bus mode of transport to require 30% of the demand to be met by battery-electric school buses in 2030 and 100% by 2050 in the electrification scenarios, ZEV and ZEV + TAX. The Bipartisan Infrastructure Law of 2021 authorizes the U.S. EPA to offer rebates to replace existing school buses with clean and zero-emission models. Although the rebates may end up replacing a small portion of the fleet, an increase in the share of electric buses within the next decade is feasible (Environmental Protection Agency 2022, World Resources Institute 2022).

Lastly, the model includes technology-specific hurdle rates, similar to discount rates used in engineering economics, to capture consumer risk aversion and other barriers to adoption. Higher hurdle rates effectively increase the weight of capital costs in calculating the levelized costs that TIMES uses when optimizing. Isik et al (2021) delved deeper into hurdle rates and their implications on the energy system modeling results. Here, hurdle rates for conventional, gasoline- or diesel-powered technologies were assigned hurdle rates of 10%. Alternative technologies like battery-electric, compressed natural gas, liquified petroleum gas, or diesel hybrid technologies were assigned hurdle rates of 15% for earlier modeling years, then reduced to 10% for later modeling years. The temporal variation is implemented to capture unmodeled barriers toward adopting new and emerging technologies in the near future, such as range anxiety, limited experience of mechanics, uncertainty in and cost of infrastructure buildout including permitting challenges.

### 2.4. Sensitivity to vehicle cost and performance

In our analysis, we hypothesized that vehicle cost and performance would be key drivers in the adoption of battery-powered vehicles. Therefore, we repeated the scenarios using an advance vehicle scenario cost and performance projections from the NREL ATB where cost and performance of vehicles—especially battery-powered vehicles—are reducing at a much faster pace than the mid-vehicle scenario. The underlying cost and performance assumptions are provided in the supplementary information (tables S11 and S12).

## 3. Results

The four scenarios were evaluated using the TIMES model. We examined model outputs including fuel consumption by transportation subsector, electricity consumption by sector, and CO_2_, NO_*X*_ and PM_2.5_ emissions.

### 3.1. Fuel consumption

**Figure 3.**
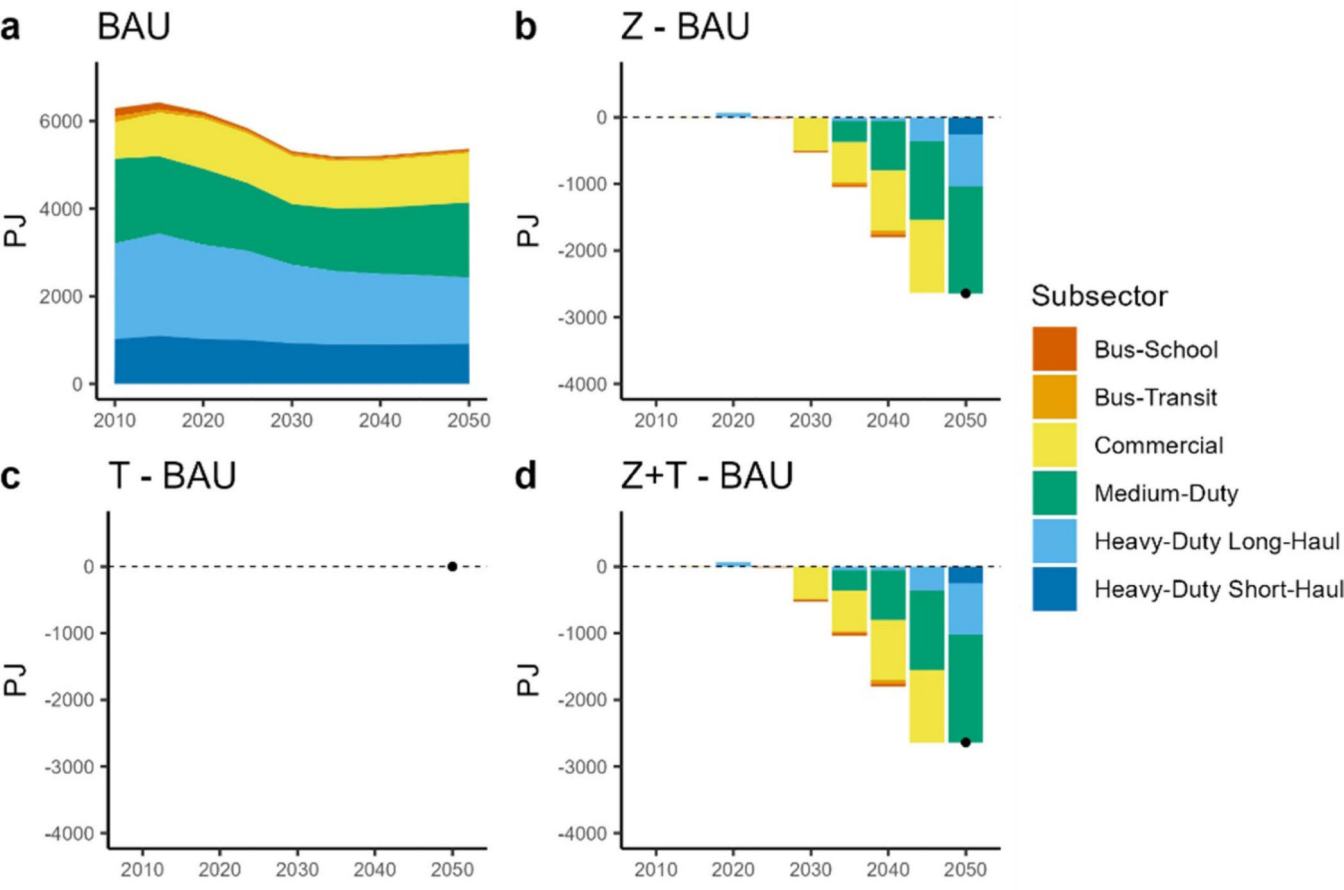
Fossil fuel consumption by transport mode. (a): total fuel consumption under BAU. (b): difference in fuel consumption between the ZEV scenario and BAU (Z - BAU). (c): difference in fuel consumption between TAX scenario and BAU (T - BAU). (d): difference in fuel consumption between the ZEV + TAX scenario and BAU (Z+T - BAU).

**Figure 4.**
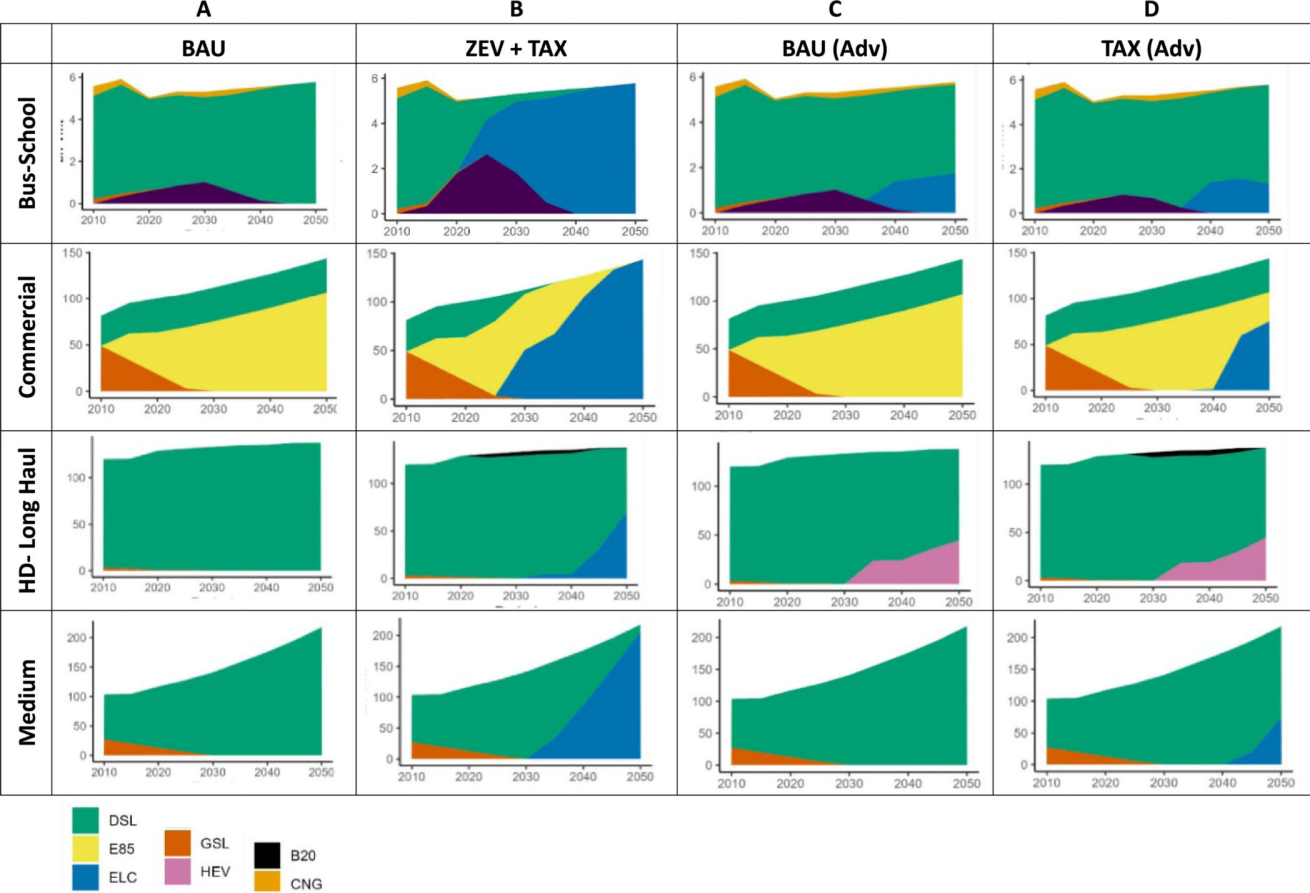
Level of demand in billion vehicle miles traveled (bn-vmt met by fuels per transport mode. Column (A) presents results for BAU under the NREL ATB’s mid-vehicle scenario cost and performance assumptions. Column (B) presents results for the ZEV + TAX scenario under the NREL ATB’s mid-vehicle scenario. Column (C) presents results for the BAU scenario under the NREL ATB’s advanced-vehicle scenario. Column (D) presents results for the TAX scenario under the NREL ATB’s advanced-vehicle scenario). Results for other modes for all scenarios are provided in the supplementary information: figures S2–S8 and S16–S22.

According to AEO projections, the on-road MHDV demand increases 60% by 2050, where the most increase (two folds) is seen in medium duty trucking. Commercial trucking and heavy-duty short-haul trucking increase by 50%, while heavy-duty long haul trucking increases by 15%. When these demand projections are simulated under the BAU scenario, the fuel consumption of MHDVs decreased from 6209 PJ in 2020 to 5365 PJ in 2050 (figure 3(a)), where most of the decrease resulted from heavy-duty long-haul vehicles. These vehicles, along with the rest of the MHDVs, are primed for efficiency improvements. Although we have not explicitly included EPA’s final rule in our analysis: GHG emissions standards for heavy-duty vehicles—Phase 3, the reductions in the BAU very much mimic the rule in reducing GHG emissions from this sector starting in 2027. The other transport modes, such as medium and commercial trucks, resulted in a steady consumption of fuel despite growing demand. This illustrates the role of efficiency gains in the transport fleet. Figure 4 shows the distribution of fuels meeting the demand per transport mode for select scenarios. In figure 4(A), the BAU trends show a continuous reliance on fossil fuels. Further analysis of the scenarios revealed the impact of sectoral policies and top-down carbon tax such that electrification of transport modes such as buses, commercial and medium duty trucks ramp up significantly (figure 4(B)). In the ZEV + TAX scenario, MHDV fleets are converted to electric resulting in a 63% reduction in fossil fuel consumption in 2050 relative to BAU (figure 3(d)). Half of the savings result from medium duty and heavy-duty long-haul modes. Starting in 2040, all the school bus demand is met by electric buses (figure 4(B)). By 2050 almost all the commercial truck, medium truck and transit bus demand and half of the heavy-duty long-haul truck demand is met by electric vehicles both in the ZEV + TAX scenario. Under the ZEV scenario, the level of electrification of the fleets was almost the same (figure 3(b)). Interestingly, the TAX scenario results in no electrification of MHDVs (figures S2–8), as well as LDVs (figure S7). However, when we changed the cost and performance assumptions using the NREL ATB’s advanced-vehicle scenario, we observed electrification of school buses, commercial and medium-duty trucks in the TAX scenario (figure 4(D)). The improved cost and performance data also result in some electrification and use of hybrid vehicles in the MHDV transport modes in the BAU scenario (figure 4(C)).

### 3.2. Electricity consumption and generation

**Figure 5.**
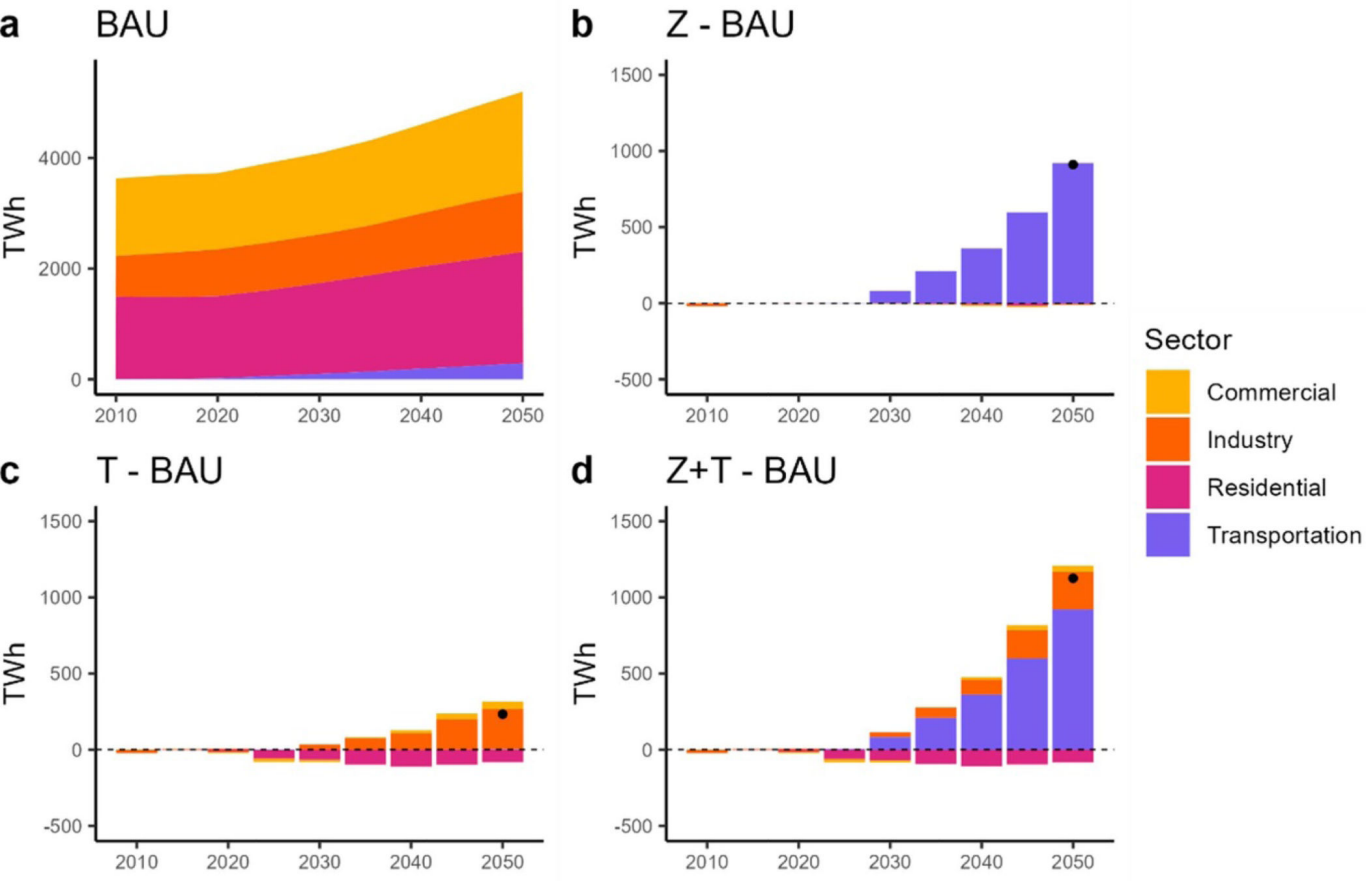
Electricity consumption by end-use. (a): total electricity consumption under BAU. (b): difference in electricity consumption between the ZEV scenario and BAU. (c): difference in electricity consumption between the TAX scenario and BAU. (d): difference in electricity consumption between the ZEV + TAX scenario and BAU. Dots show net difference.

System-level electricity consumption is projected to grow in every end-use sector under BAU, rising from 3910 TWh in 2020 to 5340 TWh in 2050 (figure 5(a)). The electricity demand increases by nearly 20% to accommodate MHDV fleet electrification in the ZEV scenario (figure 5(b)). By 2050, almost 70% of the MHDV fleet demand is met by electricity. The addition of tax in the scenarios induces additional electrification in the industrial sector (figures 5(c) and (d)). The ZEV + TAX scenario results in the greatest increase in electricity demand, meeting additional transportation and industrial electrification reaching 6600 TWh in 2050 (figure 4(D)). The building sector reduces electricity consumption under the TAX and TAX + ZEV scenarios (figures 5(c) and (d)).

**Figure 6.**
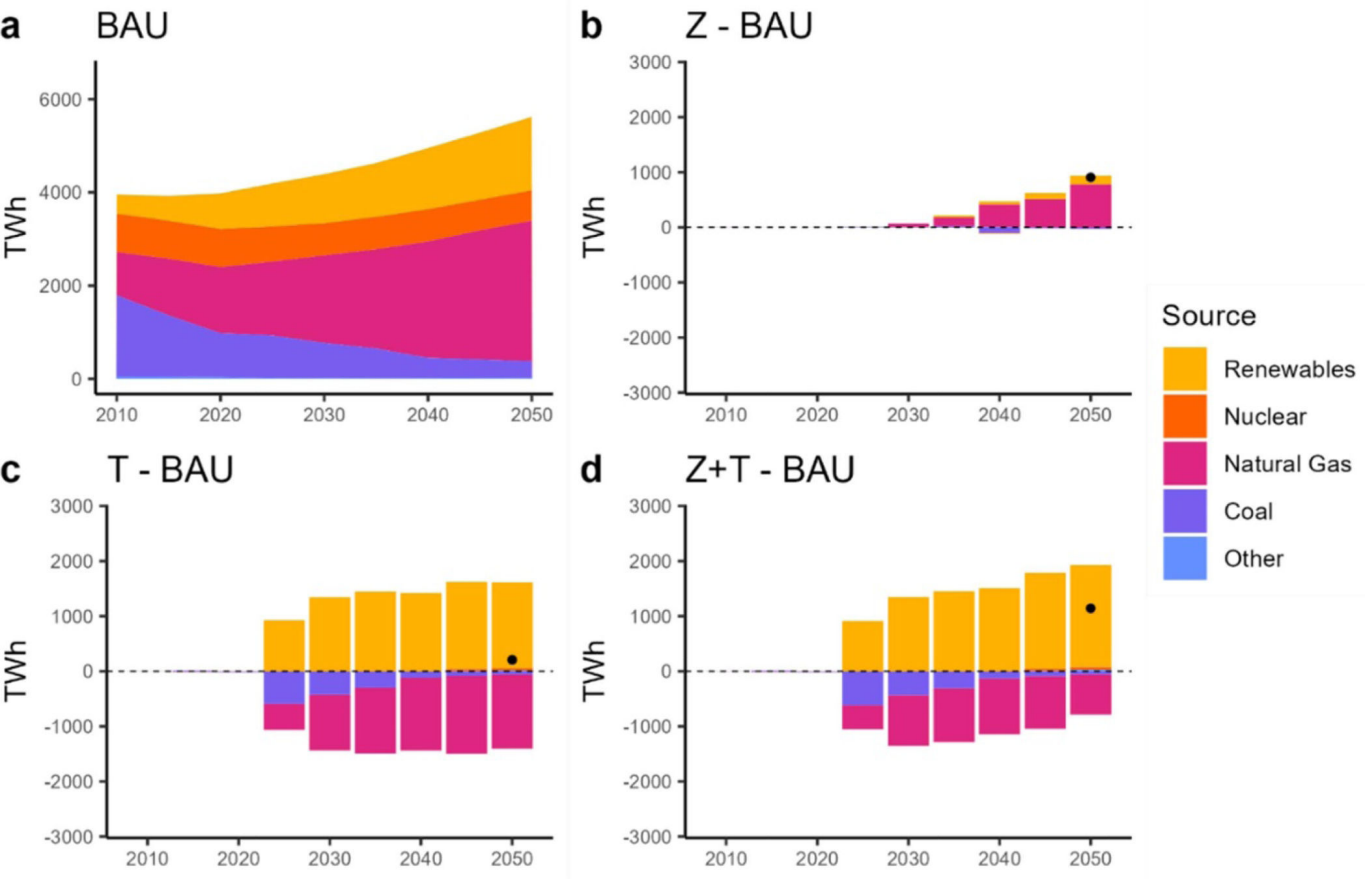
Electricity generation by source. (a): total electricity generation under BAU. (b): difference in electricity generation between the ZEV scenario and BAU. (c): difference in electricity generation between the TAX scenario and BAU. (d): difference in electricity generation between the ZEV + TAX scenario and BAU. Dots show the net difference.

**Figure 7.**
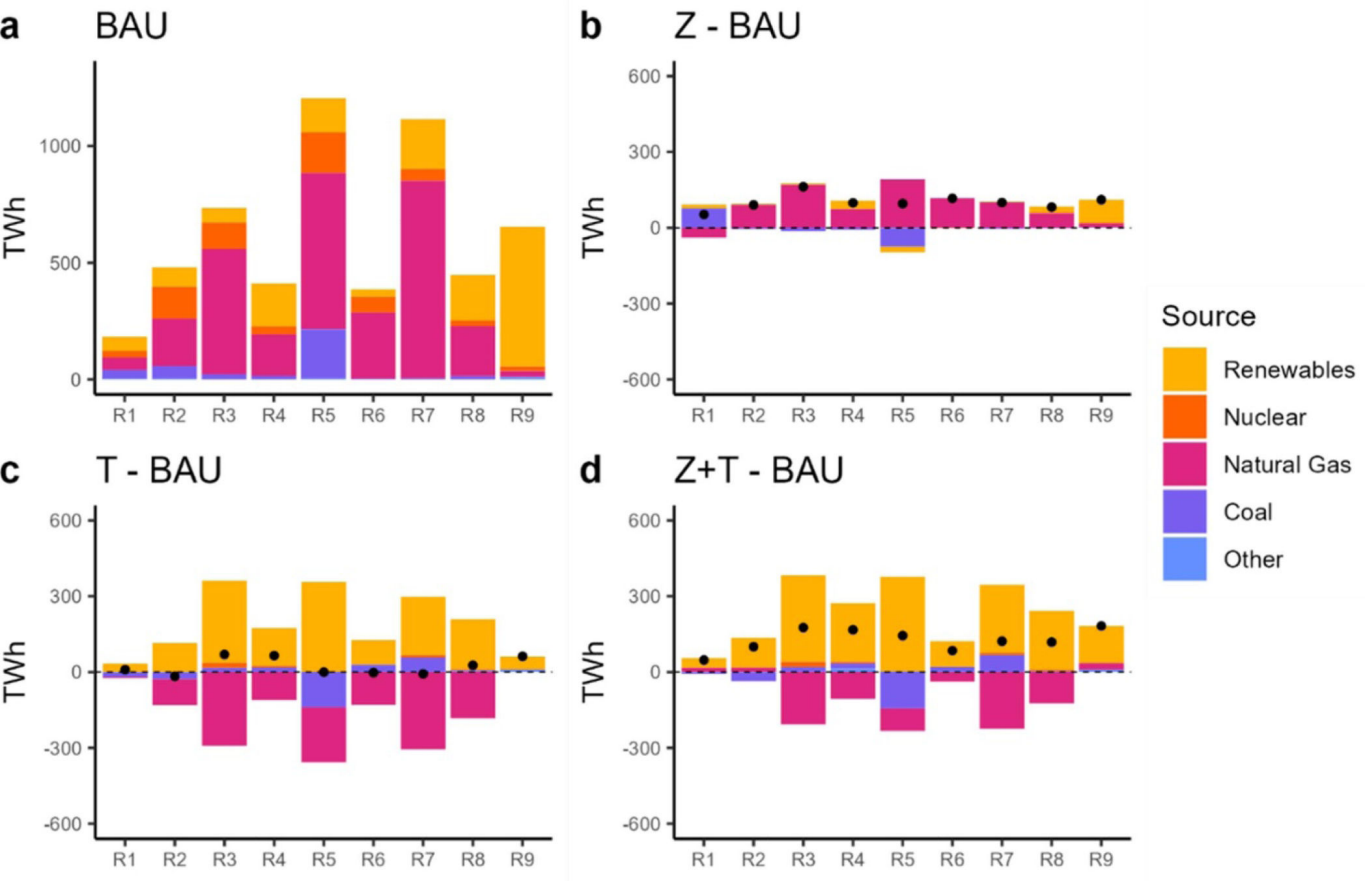
Electricity generation in 2050 by source and region. (a): 2050 electricity generation under BAU. (b): difference in electricity generation between the ZEV scenario and BAU. (c): difference in electricity generation between the TAX scenario and BAU. (d): difference in electricity generation between the ZEV + TAX scenario and BAU. Dots show the net difference.

Considering the electric generation mix, natural gas-powered electricity generation doubles from 2020 to 2050 under BAU, while coal-powered generation decreases (figure 6(a)). Solar power generation also rises five times to a total of 610 TWh by 2050. In the ZEV scenario, the electricity demand resulting from MHDV electrification is met by additional natural gas capacity (figure 6(b)). Under the TAX scenario, the CO_2_ tax leads to a 1960 TWh increase in renewable power generation and a 1730 TWh decrease in natural gas generation by 2050 relative to BAU. While there is very little change in the total electricity generation level, there is a significant switch in the generation mix (figure 6(c)). The ZEV + TAX scenario uses approximately the same amount of renewable energy as the TAX scenario and adds natural gas to meet the additional demand from MHDV fleet electrification (figure 6(d)). In total, both the TAX and TAX + ZEV scenarios retire coal capacity starting in 2025; however, the regional trends in coal retirement are heterogenous. Figure 7 illustrates electricity generation by source and region in 2050 compared to BAU. For instance, we see that Region 1 keeps the coal generation to compensate for the increased demand in the ZEV scenario (figure 7(b)), whereas Region 5 retires further capacity. Similarly, in the ZEV + TAX scenario (figure 9(d)), Region 5 retires even more coal capacity whereas Region 7 keeps some compared to BAU. The next section will explore the emission implications of these results as well.

### 3.3. Emissions

**Figure 8.**
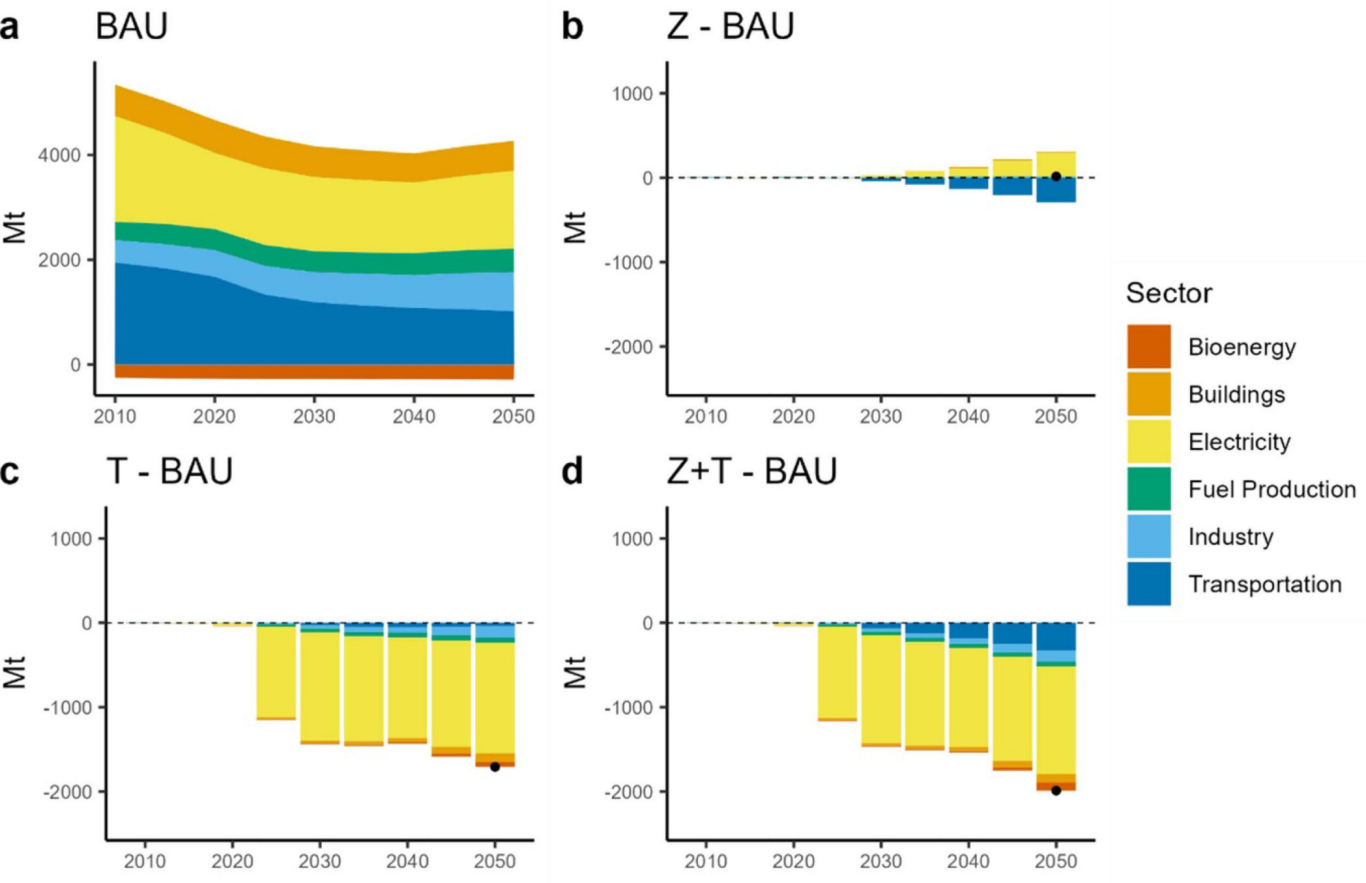
CO_2_ emissions by sector. (a): total CO_2_ emissions under BAU. (b): difference in CO_2_ emissions between the ZEV scenario and BAU. (c): difference in CO_2_ emissions between the TAX scenario and BAU. (d): difference in CO_2_ emissions between the ZEV + TAX scenario and BAU. Dots show the net difference.

Under BAU, CO_2_ emissions reduce to approximately 3900 Mt by 2050. Further along, the TAX scenarios reduce emissions too but are reduced to 2273 Mt and 1990 Mt for the TAX and ZEV + TAX scenarios, respectively. Interestingly, the ZEV scenario results in no CO_2_ change. The increased electric demand is met by additional natural gas generation, and the reduction observed in the transportation sector almost offsets the increase in electric sector emissions (figure 8(b)). The CO_2_ tax induced a transition to low CO_2_ fuels and renewable resources for electricity generation such as more wind and solar, and also biomass integrated gasification combined-cycle (BIO-IGCC). Alone, the TAX scenario does not drive electrification of the transportation sector, however it does result in more CO_2_ reductions compared to the ZEV scenarios due to the transition to a cleaner grid. We observed the greatest savings when the tax and mandate were applied in tandem (figures 8(c) and (d)), resulting in a 51% reduction in CO_2_ emissions in 2050 from BAU.

**Figure 9.**
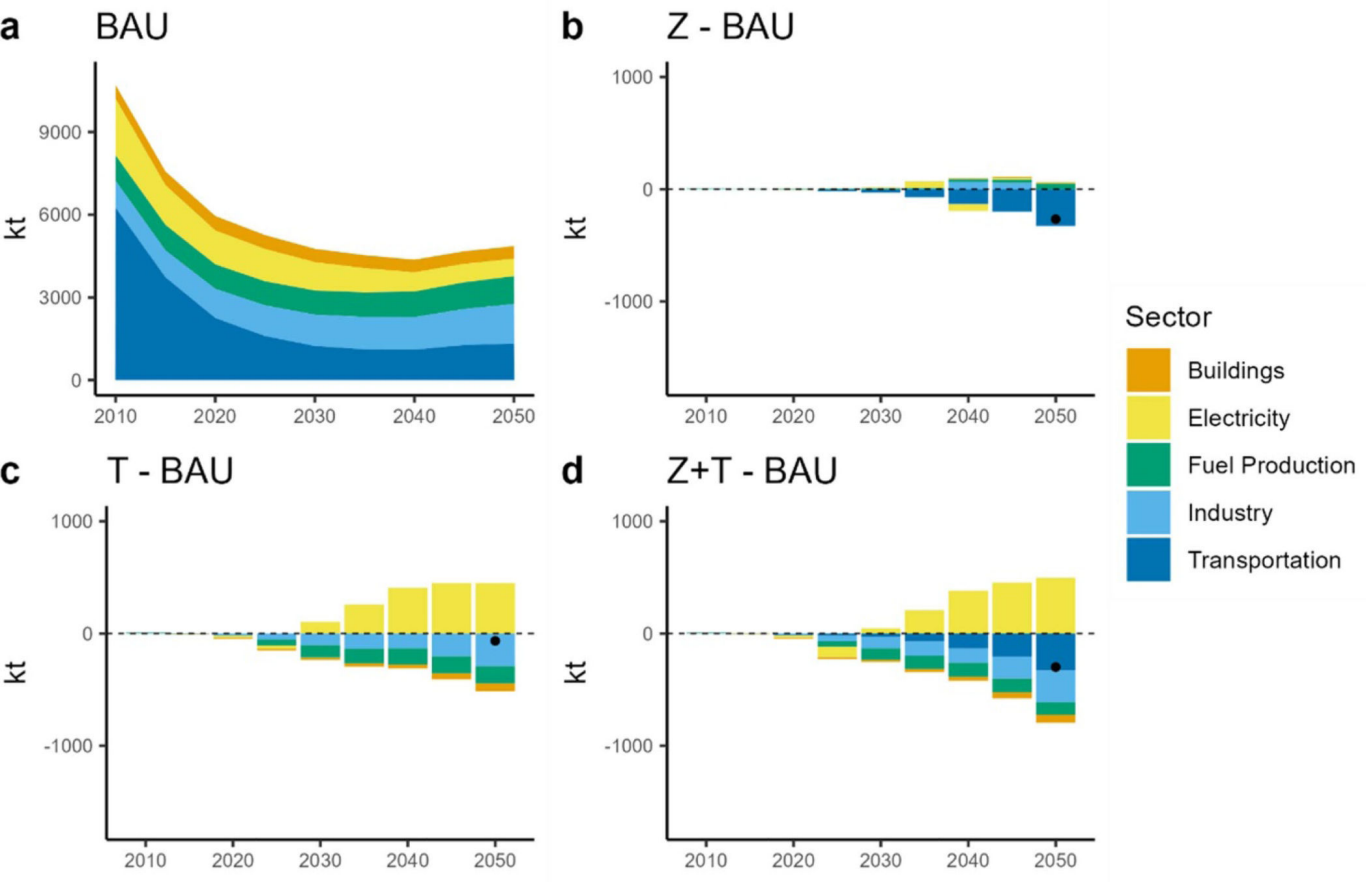
NO_*X*_ emissions by sector. (a): total NO_*X*_ emissions under BAU. (b) difference in NO_*X*_ emissions between the ZEV scenario and BAU. (c): difference in NO_*X*_ emissions between the TAX scenario and BAU. (d): difference in NO_*X*_ emissions between the ZEV + TAX scenario and BAU. Dots show the net difference.

Under various environmental and energy standards and regulations, the air emission impact of the transportation sector has been on a declining trend. Under BAU, NO_*X*_ emissions decrease from 5950 kt in 2020 to 4862 kt in 2050, driven by more stringent standards on tailpipe NO_*X*_ emissions in the transport sector (figure 9(a)) (U.S. Environmental Protection Agency 2014b). The ZEV mandate decreases transport sector NO_*X*_ emissions further, with an additional 325 kt and 328 kt avoided emissions in the ZEV and ZEV + TAX scenarios, respectively (figures 9(b) and (d)). The TAX scenario reveals an unexpected lack of overall NO_*X*_ benefits due to a 69% rise in electric sector emissions (figure 9(c)). The rise in electric sector NO_*X*_ emissions in the TAX and TAX + ZEV scenarios are especially surprising given that the tax had succeeded in reducing the share of fossil fuels and increasing the share of renewables on the grid.

**Figure 10.**
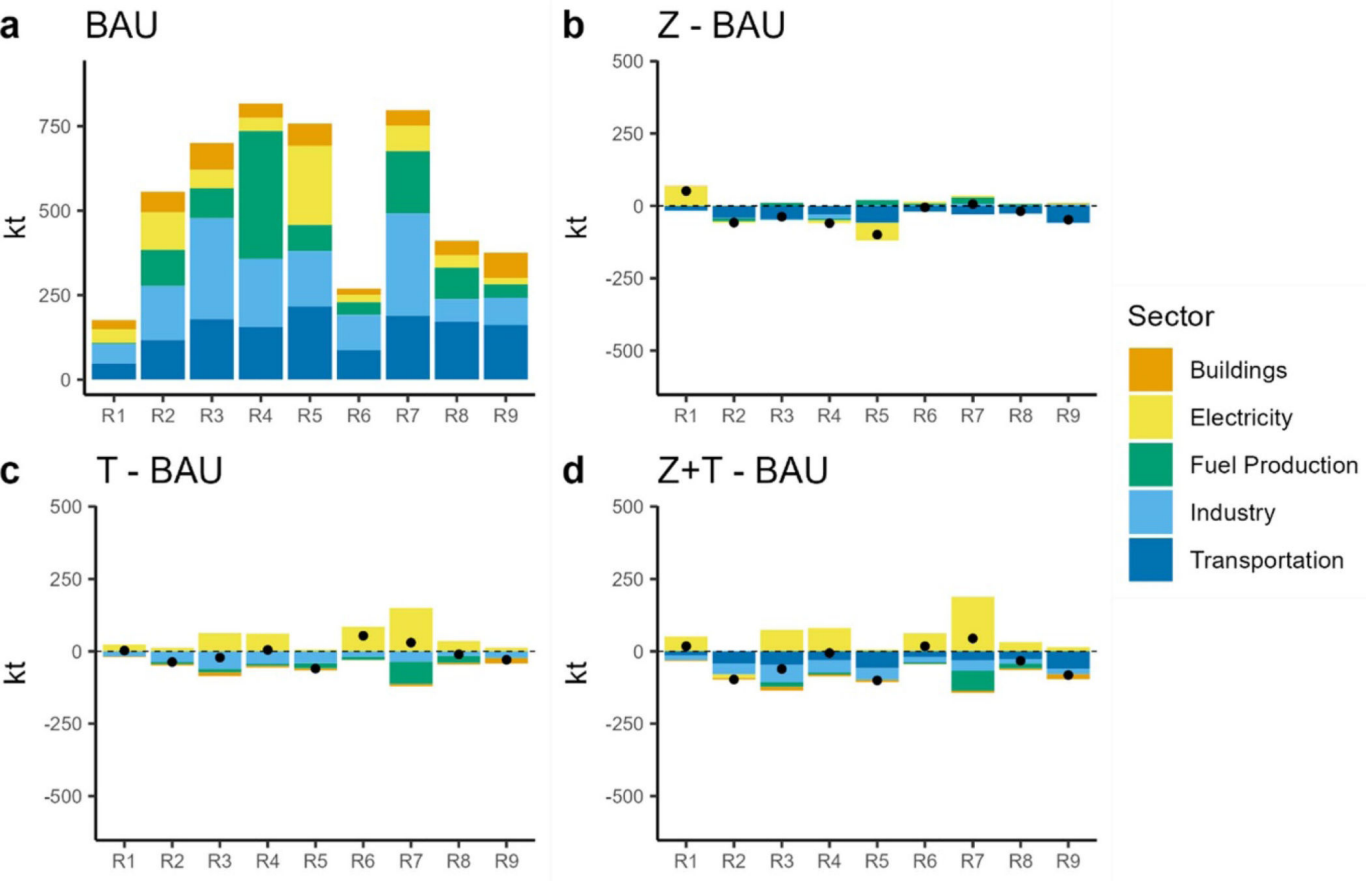
NO_*X*_ emissions in 2050 by sector and region. (a): 2050 NO_*X*_ emissions under BAU. (b): difference in NO_*X*_ emissions between the ZEV scenario and BAU. (c): difference in NO_*X*_ emissions between the TAX scenario and BAU. (d): difference in NO_*X*_ emissions between the ZEV + TAX scenario and BAU. Dots show the net difference.

Close examination of the NO_*X*_ emissions and electricity generation fuel mix at a regional level (figures 10 and 7) reveals that the rise in electric sector NO_*X*_ emissions comes primarily from Regions 1 (New England covering ME, VT, NH, MA, CT, RI), 6 (East South Central covering KY, TN, MS and AL) and 7 (West South Central covering OK, AR, LA, TX) (figures 10(b)–(d)). These regions swapped out natural gas generation with renewables, under the CO_2_ tax in 2050. The renewables include BIO-IGCC along with wind and solar. However, we also observe some lifetime extension of coal power plants. Here, we dissect this issue starting with using biomass in the power sector, specifically BIO-IGCC, although this technology reduces CO_2_ emissions, the NO_*x*_ EFs per unit of electricity generated is high compared to natural gas. Secondly, these scenarios retire less coal capacity compared to BAU (figure 10). The delayed retirement of coal in these regions may seem counterintuitive given the CO_2_ tax. The TIMES algorithm employs perfect foresight and finds delaying coal capacity retirement a cost-effective strategy to meet the increased electricity demand while also meeting air quality constraints. Compared to new wind and solar capacity, these plants will not incur any investment cost, and the model will include their O&M costs along with the carbon tax in the total discounted cost calculations. Their O&M cost will also include air pollution control equipment. One other thing we observed was related to investments in low-NO_*x*_ burners for power plants. Power sector NO_*x*_ and SO_2_ emissions are regulated under the EPA’s cross-state air pollution rule (CSAPR) (U.S. Environmental Protection Agency 2022e). Cross-state air pollution, also known as interstate air pollution or transported air pollution, is emitted at one location (upwind) and then blown by the wind to another location (downwind). The rule aims to reduce NO_*x*_ emissions from power plants in 12 states in the eastern United States. To model this, we included regional upper limits for electric NO_*x*_ and SO_2_ emissions for Regions 2, 3, 4 and 5.

Although the rule covers some states in Region 6, it did not cover all the region. Similarly, Regions 1, 7, 8 and 9 did not have any regional limits. We added an overall nation-wide upper limit on NO_*x*_ and SO_2_ to cover nationwide limits to meet air quality standards. In all the scenarios, the power sector NO_*x*_ and SO_2_ emissions are below the limits imposed (see tables S6 and S7 in the supplementary information on the total electric NO_*x*_ levels and how they compare to overall limits). The retirement of some coal capacity relieves the NO_*x*_ budgets allocated to the regions. While running the coal plants, the model chooses to invest less capacity on low NO_*x*_ burners; this results in an increase in NO_*x*_ emissions. This strategy was also cheaper than further adding renewable generation in those regions from a total cost accounting perspective.

**Figure 11.**
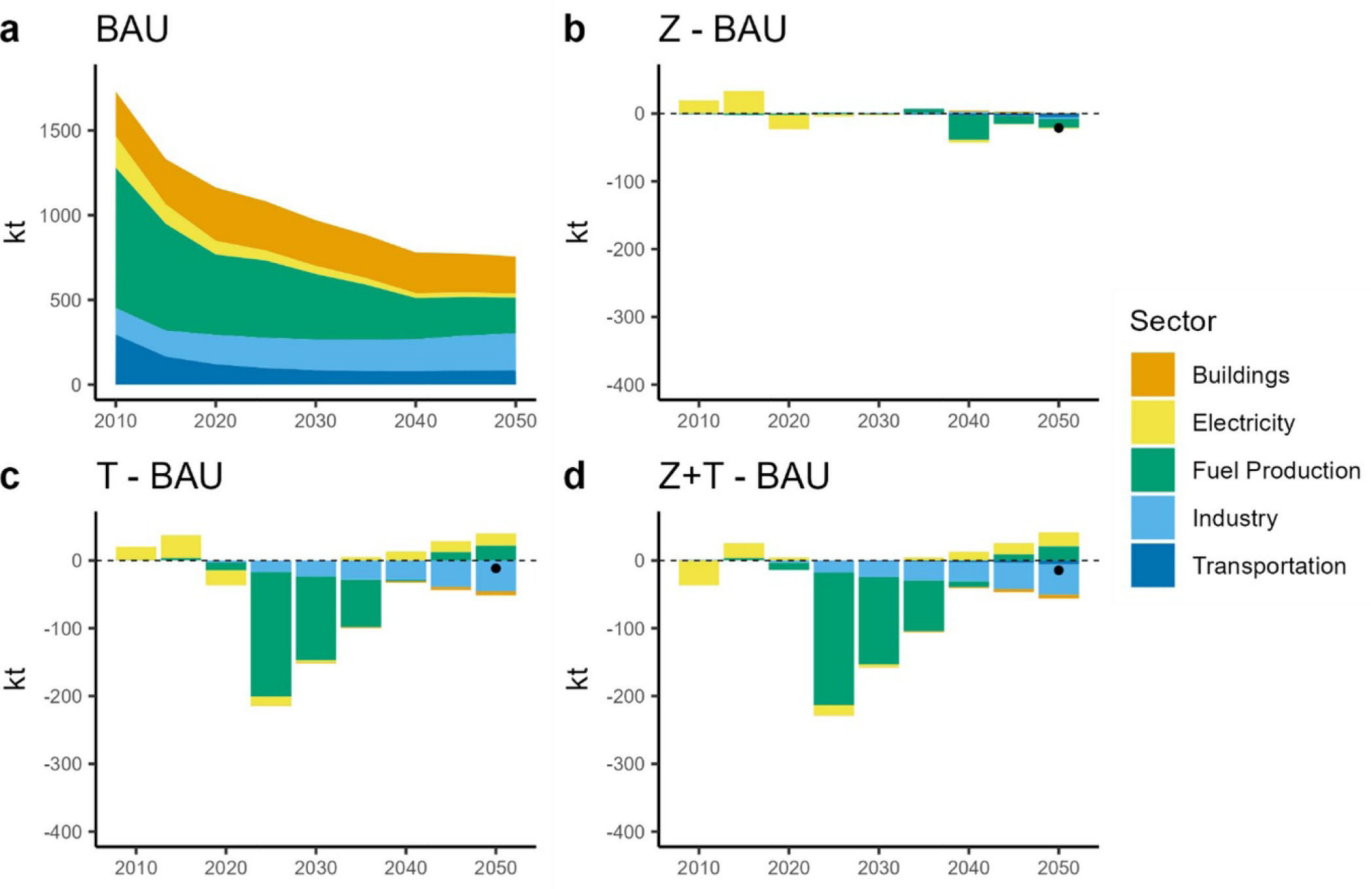
PM_2.5_ emissions by sector. (a): total PM_2.5_ emissions under BAU. (b): difference in PM_2.5_ emissions between the ZEV scenario and BAU. (c): difference in PM_2.5_ emissions between the TAX scenario and BAU. (d): difference in PM_2.5_ emissions between the ZEV + TAX scenario and BAU. Dots show the net difference.

**Figure 12.**
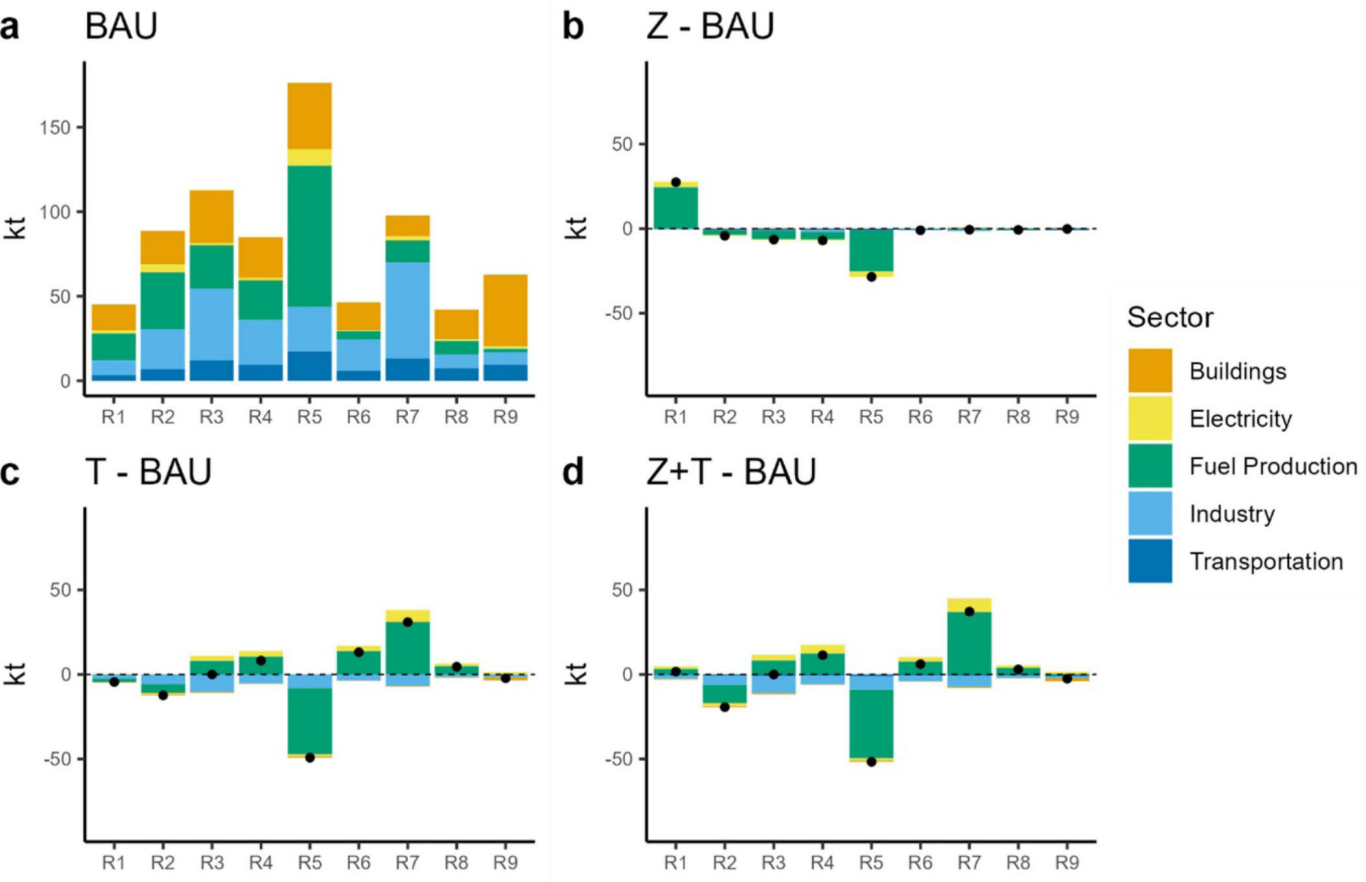
PM_2.5_ emissions in 2050 by sector and region. (a): 2050 PM_2.5_ emissions under BAU. (b): difference in PM_2.5_ emissions between the ZEV scenario and BAU. (c): difference in PM_2.5_ emissions between the TAX scenario and BAU. (d): difference in PM_2.5_ emissions between the ZEV + TAX scenario and BAU. Dots show the net difference.

Another air pollutant of concern is ambient fine PM_2.5_ which is one of the leading environmental health risk factors contributing to many diseases. According to the EPA’s 2017 National Emission Inventory, the resource extraction sector is one of the biggest contributors to PM_2.5_ emissions. Although transportation related PM_2.5_ emissions are lower than most other parts of the energy system, their prevalent exposure pathway is still important and contributes to the total PM_2.5_ disease burden (McDuffie et al 2021). Under BAU, PM_2.5_ emissions decrease from 1163 kt in 2020 to 756 kt in 2050 in BAU, driven by the resource supply sector as coal powered electric generation capacity is retired (figure 11(a)). Earlier, the coal retirement PM_2.5_ benefits are observed in the tax scenarios, but these benefits diminish in later years when BAU also retires coal (figures 11(c) and (d)). Although, total nation-wide PM_2.5_ emissions decrease, regional differences emerge. A rise in PM_2.5_ emissions in Region 4 (West North Central), Region 6 (East South Central) and Region 7 (West South Central) is seen in the TAX scenarios in 2050 (figures 12(c) and (d)). These regions keep some of the coal capacity running well into 2050 (figure 7). Region 6 and Region 7 are major coal supply regions in the United States of America. Prevalent use of coal for electricity generation increases fuel demand, therefore increasing extraction and production related PM_2.5_ emissions. Transport sector PM_2.5_ emissions are small in magnitude compared to emissions from other sectors (figures 11 and 12).

Results suggest that transitioning to 100% ZEV sales in MHDV fleets nationally by 2050 would yield CO_2_ benefits in the United States of America when additional cost and performance improvements are observed and the electric grid transforms to renewables at a faster pace. The results indicated that the time of charging and the pace of grid emission intensity reductions influenced the level of emissions benefits. As of 2023, some states are already adopting policies that would enforce the ramped up sale of clean vehicles and trucks (NRDC 2022). These initiatives were led by the State of California which introduced Advanced Clean Trucks and the Advanced Clean Fleets Program and many states are adopting similar pathways (CARB 2022). This transition would require an estimated 18% increase in electricity generation (1000 TWh) in 2050 compared to the BAU case, though almost 75% of MHDV fleet demand is met by battery-powered vehicles. The model meets this demand in the ZEV scenario (no CO_2_ tax) with additional natural gas capacity in most regions. The resulting increase in CO_2_ emissions in the electric sector was on par with the decrease in tailpipe CO_2_ emissions from the MHDV sectors when conservative improvements in cost and the performance of MHDVs are assumed and variation in charging time is considered.

The TAX and ZEV + TAX scenarios reflect the cross-sector emissions impact of a $100 ton^−1^ CO_2_ (in 2020 American dollars) system-wide CO_2_ tax. Differences between these reveal insights into the electric generation mix. Although the ZEV scenario results in no net CO_2_ emission benefits, implementing the tax further increases system-level CO_2_ reduction by converting nearly half of the electric grid capacity to low-emission renewable sources. This scenario results in the halving of CO_2_ emissions in 2050 compared to BAU. At the regional level, the CO_2_ tax had complicated effects due to the existing coal generating capacity and still prevalent cost-differentials when it comes to renewable energy capital investment costs. In Region 6 (East South Central) and Region 7 (West South Central), the model delays coal retirement compared to BAU. At the regional level, these delays take away some of the emission benefits resulting due to electrification and the introduction of renewables.

The impact of the electrification target on NO_*X*_ and PM_2.5_ emissions shows some change at the system level. Transport NO_*X*_ emissions are projected to decrease significantly under BAU due to stringent tailpipe NO_*X*_ emission standards. With MHDV electrification, the ZEV scenario showed a 16% (210 kt) additional benefit compared to BAU by 2050. This result is consistent with the outcome of PM_2.5_ emission benefits in the ZEV scenario. Although the emissions reductions were small in magnitude; the resulting 5% improvement may still be significant due to the localization of transport sector PM_2.5_ emissions along major roadways.

## 4. Conclusions

We used the EPAUS9rT TIMES model to assess the emission impacts of an electrification sales target for medium- and heavy-duty ZEVs that grows to 100% by 2050, both with and without a CO_2_ tax. We found that transitioning to 100% ZEV sales nationally by 2050, for MDVs and HDVs, would yield net CO_2_ benefits should the electric grid transition to low carbon sources simultaneously. Bistline et al (2023) analyzed an ensemble of energy system modeling scenarios that evaluated the Inflation Reduction Act (IRA) 2022. They concluded that most IRA-induced mitigation comes from electricity, representing 38%–80% of 2030 reductions (64% average) from the reference in economy-wide models. There is consistency across models that the IRA will accelerate power sector decarbonization. Although we did not include IRA provisions in our study, one can then expect the transitions to be materialized. The electrification of MHDVs is illustrated to be sensitive to cost and performance, the carbon intensity of the electric grid as well as the charging time of the MHDV fleets. Earlier studies excluding demand load profiles concluded net CO_2_ benefits, however, considering the distinct time of charging profiles per transport mode, our analysis showed that the benefits would diminish due to the increase in power sector emissions. An all-sector CO_2_ tax of $100 ton^−1^ CO_2_ (in 2020 American dollars), rising by 5% per year, would significantly increase these benefits but will not drive the pace of transportation electrification. The pace of transportation electrification increased when the assumptions for the cost of vehicles decreased, and performance improvements accelerated. From a policy perspective, these results support the utility of measures that would drive clean electricity transition goals along with a national electrification goal similar to the medium and heavy-duty electrification MOU that has been adopted by 16 states and DC.

In interpreting the results, one should note three main assumptions surrounding this analysis. First, that travel demand is specified exogenously in TIMES. Demand is inelastic to energy prices. With exogenous demands, mode choice decisions within the TIMES model are limited. Secondly, while TIMES allows simulation at the U.S. Census Division level, many policies that may impact the electric sector response are specified at the state level (e.g. Renewable Portfolio Standards in the electric sector), and thus approximations are required to map these to the model’s regions. Lastly, the level of BEV penetration we observed in this analysis for the MOU scenarios would not change should other levers be included. As the MOU is designed as a lower bound on the scenarios, this means that no matter the costs, the TIMES model will invest in BEVs. The tax scenario with the level of BEVs we saw without the MOU is more flexible. In that sense, sectoral policies are then shown to result in more reductions, rather than top-down policies covering the whole energy system.

This study focused on the air and carbon emission implications of the electrification of MDVs and HDVs. Observing increased electrification of the LDV fleets along with MHDV electrification could lead to further efficiencies and synergies with charging availability and demand management. The charging timing is increasingly important to model since such a high electricity demand could either cause reliability issues or smooth the load curve on a decarbonized grid. This analysis illustrated the system-level emission implications of a scenario where electric vehicle mandates are combined with CO_2_ tax. Future studies could focus on the interaction between policies incentivizing electric vehicle uptake (e.g. newly proposed used and new EV tax credits) and policies (beyond the CO_2_ tax) to decarbonize other sectors.

## Supplementary Material

SI

## Data Availability

The study utilized US EPA’s TIMES database version: EPAUS9rT_v20.4. The modified version of the database can be made available upon request. All data that support the findings of this study are included within the article (and any supplementary information files).

## References

[R1] Stemming warming and augmenting pay Act, H.R 4058, 115th Cong 2019 (available at: www.congress.gov/bill/116th-congress/house-bill/4058)

[R2] Aryanpur V and Rogan F 2024 Decarbonising road freight transport: the role of zero-emission trucks and intangible costs Nat. Sci. Rep. 14 2113 10.1038/s41598-024-52682-4 PMC10810084 38267587

[R3] Bahn O, Marcy M, Vaillancourt K and Waaub J-P 2013 Electrification of the Canadian road transportation sector: a 2050 outlook with TIMES-Canada Energy Policy 62 593–606

[R4] Bipartisan Infrastructure Law (BIL) 2021 Infrastructure investment and jobs Act, Public Law No. 117–58, 135 STAT. 429 (available at: www.congress.gov/bill/117th-congress/house-bill/3684/textwww.congress.gov/117/plaws/publ58/PLAW-117publ58.pdf)

[R5] Bistline J 2023 Emissions and energy impacts of the inflation reduction Act. Sci. 380 1324–7 10.1126/science.adg3781 PMC10336889 37384684

[R6] Bloomberg NEF 2021 Battery pack prices fall to an average of $132/kWh, but rising commodity prices start to bite (available at: https://about.bnef.com/blog/battery-pack-prices-fall-to-an-average-of-132-kwh-but-rising-commodity-prices-start-to-bite/)

[R7] Cabukoglu E, Georges G, Küng L, Pareschi G and Boulouchos K 2018 Battery-electric propulsion: an option for heavy-duty vehicles? Results from a Swiss case-study Transp. Res. C 88 107–23

[R8] California Air Resources Board 2022 Advanced clean cars program (available at: ww2.arb.ca.gov/our-work/programs/advanced-clean-cars-program)

[R9] Choma EF, Evans JS, Gomez-Ibanez JA, Di Q, Schwartz JD, Hammitt JK and Spengler JD 2021 Health benefits of decreases in on-road transportation emissions in the United States from 2008 to 2017 Proc. Natl Acad. Sci. USA 118 e2107402118 10.1073/pnas.2107402118 PMC8713776 34903648

[R10] Doluweera G, Hahn F, Bergerson J and Pruckner M 2020 A scenario-based study on the impacts of electric vehicles on energy consumption and sustainability in Alberta Appl. Energy 268 114961

[R11] Dubin K 2021 EIA projects renewables share of U.S. electricity generation mix will double by 2050 (Energy Information Administration) (available at: www.eia.gov/todayinenergy/detail.php?id=46676) (10.1215/03616878-9349114)

[R12] Energy Information Administration 2020 Annual Energy Outlook 2020 (available at: https://www.eia.gov/outlooks/aeo/pdf/aeo2020.pdf)

[R13] Energy Information Administration 2022b Table 2.1.A. Coal: consumption for electricity generation Electric Power Monthly (available at: www.eia.gov/electricity/monthly/epm_table_grapher.php?t=table_2_01_a)

[R14] Environmental Protection Agency 2022 Clean school bus program (website) (available at: www.epa.gov/cleanschoolbus)

[R15] EPA 2017 (available at: https://www.epa.gov/air-emissions-inventories/2017-national-emissions-inventory-nei-data)

[R16] Gross S 2020 The challenge of decarbonizing heavy transport (available at: www.brookings.edu/wp-content/uploads/2020/09/FP_20201001_challenge_of_decarbonizing_heavy_transport.pdf)

[R17] Harper G 2019 Recycling lithium-ion batteries from electric vehicles Nature 575 75–86 10.1038/s41586-019-1682-5 31695206

[R18] Hunter C, Penev M, Reznicek E, Lustbader J, Birky A and Zhang C 2021 Spatial and temporal analysis of the total cost of ownership for class 8 tractors and class 4 parcel delivery trucks NREL/TP-5400–71796 (National Renewable Energy Laboratory) (available at: www.nrel.gov/docs/fy21osti/71796.pdf)

[R19] Inflation Reduction Act 2022 (IRA) of 2022 Public Law No. 117–169, 136 STAT. 1818 (available at: www.congress.gov/bill/117th-congress/house-bill/5376/text; www.congress.gov/117/plaws/publ169/PLAW-117publ169.pdf)

[R20] IPCC 2021 Climate Change 2021: the Physical Science Basis Contribution of Working Group I to the Sixth Assessment Report of the Intergovernmental Panel on Climate Change ed V Masson-Delmotte et al (Cambridge University Press) accepted

[R21] Isik M, Dodder R and Kaplan PO 2021 Transportation emissions scenarios for New York City under different carbon intensities of electricity and electric vehicle adoption rates Nat. Energy 6 92–104 10.1038/s41560-020-00740-2 PMC8597912 34804594

[R22] Jordan K, Adams P, Jaramillo P and Muller N 2023 Closing the gap: achieving U.S. climate goals beyond the Inflation Reduction Act Renew. Sustain. Energy Transit. 4 100065

[R23] Keller V, Lyseng B, Wade C, Scholtysik S, Fowler M, Donald J, Palmer-Wilson K, Robertson B, Wild P and Rowe A 2019 Electricity system and emission impact of direct and indirect electrification of heavy-duty transportation Energy 172 740–51

[R24] Kriit HK, Sommar JN, Forsberg B, Åström S, Svensson M and Johansson C 2021 A health economic assessment of air pollution effects under climate neutral vehicle fleet scenarios in Stockholm, Sweden J. Transp. Health 22 101084

[R25] Lajevardi SM, Axsen J and Crawford C 2019 Comparing alternative heavy-duty drivetrains based on GHG emissions, ownership and abatement costs: simulations of freight routes in British Columbia Transp. Res. D 76 19–55

[R26] LBNL 2021 Medium-& heavy-duty electric vehicle infrastructure load, operations and deployment tool (HEVI-LOAD): methods, scenarios, and load profiles by Bin Wang, Cong Zhang Slide Deck (available at: https://efiling.energy.ca.gov/getdocument.aspx?tn=234209)

[R27] Ledna C, Muratori M, Yip A, Jadun P and Hoehne C 2022 Decarbonizing medium-& heavy-duty on-road vehicles: zero-emission vehicles cost analysis No. NREL/TP-5400–82081 (National Renewable Energy Lab.(NREL))

[R28] Lee D-Y and Thomas VM 2017 Parametric modeling approach for economic and environmental life cycle assessment of medium-duty truck electrification J. Clean. Prod. 142 3300–21

[R29] Lenox C 2022 EPAUS9rT—An energy systems database for use with the TIMES model (U.S. Environmental Protection Agency) (available at: www.epa.gov/air-research/epaus9rt-energy-systems-database-use-times-model)

[R30] Lenox C, Dodder R, Gage C, Loughlin D, Kaplan O and Yelverton W 2013 EPA U.S. nine-region markal database, database documentation EPA/600/B-13/203 (U.S. Environmental Protection Agency) (available at: https://cfpub.epa.gov/si/si_public_record_report.cfm?Lab=NRMRL&dirEntryId=278925)

[R31] Liimatainen H 2021 Truck electrification has minor grid impacts Nat. Energy 6 580–1

[R32] Liu Y, Wu S, Chen H, Federici M, Perricone G, Li Y, Lv G, Munir S, Luo Z and Mao B 2022 Brake wear induced PM10 emissions during the world harmonised light-duty vehicle test procedure-brake cycle J. Clean. Prod. 361 132278

[R33] Loulou R 2016 Documentation for the TIMES model Energy Technology Systems Analysis Programme

[R34] Lu J, Shan R, Kittner N, Hu W and Zhang N 2023 Emission reductions from heavy-duty freight electrification aided by smart fleet management Transp. Res. D 121 103846

[R35] Mai T 2018 Electrification futures study: scenarios of electric technology adoption and power consumption for the United States

[R36] McDuffie EE 2021 Source sector and fuel contributions to ambient PM2.5 and attributable mortality across multiple spatial scales Nat. Commun. 12 3594 34127654 10.1038/s41467-021-23853-y PMC8203641

[R37] McNeil WH, Tong F, Harley RA, Auffhammer M and Scown CD 2024 Corridor-level impacts of battery-electric heavy-duty trucks and the effects of policy in the United States corridor-level impacts of battery-electric heavy-duty trucks and the effects of policy in the United States Environ. Sci. Technol. 58 33–42 38109378 10.1021/acs.est.3c05139 PMC10785805

[R38] Mukherjee A, McCarthy MC, Brown SG, Huang S, Landsberg K and Eisinger DS 2020 Influence of roadway emissions on near-road PM2.5: monitoring data analysis and implications Transp. Res. D 86 102442

[R39] Muratori M 2021 The rise of electric vehicles—2020 status and future expectations Prog. In Energy 3 022002

[R40] Muratori M, Smith SJ, Kyle GP, Link R, Mignone BK and Kheshgi HS 2017 Role of the freight sector in future climate mitigation scenarios Environ. Sci. Technol. 51 3526–33 28240022 10.1021/acs.est.6b04515

[R41] Natural Resources Defense Council (NRDC) 2022 States embrace the transition to clean cars (available at: www.nrdc.org/bio/kathy-harris/states-embrace-transition-clean-cars)

[R42] Noll B, Del Val S, Schmidt TS and Steffen B 2022 Analyzing the competitiveness of low-carbon drive-technologies in road-freight: a total cost of ownership analysis in Europe Appl. Energy 306 118079

[R43] NREL (National Renewable Energy Laboratory) 2023 2022 transportation annual technology baseline (National Renewable Energy Laboratory) (available at: https://atb.nrel.gov)

[R44] Ou Y, Kittner N, Babaee S, Smith SJ, Nolte CG and Loughlin DH 2021 Evaluating long-term emission impacts of large-scale electric vehicle deployment in the US using a human-Earth systems model Appl. Energy 300 117364 10.1016/j.apenergy.2021.117364 PMC8576614 34764534

[R45] Phadke A, Khandekar A, Abhyankar N, Wooley D and Rajagopal D 2021 Why regional and long-haul trucks are primed for electrification now (available at: 10.2172/1834571; www.osti.gov/servlets/purl/1834571)

[R46] Plötz P, Gnann T, Jochem P, Yilmaz HU¨ and Kaschub T 2019 Impact of electric trucks powered by overhead lines on the European electricity system and CO2 emissions Energy Policy 130 32–40

[R47] Prina MG, Manzolini G, Moser D, Nastasi B and Sparber W 2020 Classification and challenges of bottom-up energy system models—A review Renew. Sustain. Energy Rev. 129 109917

[R48] Raju ASK, Wallerstein BR and Johnson KC 2021 Achieving NOx and greenhouse gas emissions goals in California’s heavy-duty transportation sector Transp. Res. D 97 102881

[R49] Rockstrom J, Gaffney O, Rogelj J, Meinshausen M, Nakicenovic N and Schellnhuber HJ 2017 A roadmap for rapid decarbonization Science 355 1269–71 28336628 10.1126/science.aah3443

[R50] Roth MB, Adams PJ, Jaramillo P and Muller NZ 2020 Near term carbon tax policy in the US economy: limits to deep decarbonization Environ. Res. Commun. 2 051004

[R51] Rowangould GM 2013 A census of the US near-roadway population: public health and environmental justice considerations Transp. Res. D 25 59–67

[R52] Schill W-P and Gerbaulet C 2015 Power system impacts of electric vehicles in Germany: charging with coal or renewables? Appl. Energy 156 185–96

[R53] Sen B, Ercan T and Tatari O 2017 Does a battery-electric truck make a difference?—Life cycle emissions, costs, and externality analysis of alternative fuel-powered class 8 heavy-duty trucks in the United States J. Clean. Prod. 141 110–21

[R54] Spangher L, Gorman W, Bauer G, Xu Y and Atkinson C 2019 Quantifying the impact of U.S. electric vehicle sales on light-duty vehicle fleet CO2 emissions using a novel agent-based simulation Transp. Res. D 72 358–77

[R55] State of California, State of Colorado State of Connecticut, District of Columbia, State of Hawaii, State of Maine, State of Maryland, State of Massachusetts, State of New Jersey, State of New York, State of North Carolina, State of Oregon, State of Pennsylvania, State of Rhode Island, State of Vermont, and State of Washington 2020 Multi-state zero emission medium- and heavy-duty vehicle initiative—memorandum of understanding (available at: www.transportpolicy.net/wp-content/uploads/2021/08/multistate-truck-zev-governors-mou-20200714.pdf)

[R56] State of California 2018 The assembly bill 2127 second electric vehicle charging infrastructure assessment (available at: www.energy.ca.gov/data-reports/reports/electric-vehicle-charging-infrastructure-assessment-ab-2127)

[R57] Talebian H, Herrera OE, Tran M and Mérida W 2018 Electrification of road freight transport: policy implications in British Columbia Energy Policy 115 109–18

[R58] Thomas CE 2012 How green are electric vehicles? Int. J. Hydrog. Energy 37 6053–62

[R59] Tong F, Jenn A, Wolfson D, Scown CD and Auffhammer M 2021 Health and climate impacts from long-haul truck electrification Environ. Sci. Technol. 55 8514–23 34124900 10.1021/acs.est.1c01273

[R60] U.S. Census Bureau 2022 Census regions and divisions of the United States (available at: www2.census.gov/geo/pdfs/maps-data/maps/reference/us_regdiv.pdf)

[R61] U.S. Environmental Protection Agency 2010a Light-duty vehicle greenhouse gas emission standards and corporate average fuel economy standards; final rule RIN: 2060-AP58 (available at: www.govinfo.gov/content/pkg/FR-2018-12-21/pdf/2018-27160.pdf)

[R62] U.S. Environmental Protection Agency 2010b Light-duty vehicle greenhouse gas emission standards and corporate average fuel economy standards; final rule RIN: 2127-AK50 (available at: www.govinfo.gov/content/pkg/FR-2012-10-15/pdf/2012-21972.pdf)

[R63] U.S. Environmental Protection Agency 2012 2017 and later model year light-duty vehicle greenhouse gas emissions and corporate average fuel economy standards RIN: 2127-AK79 (available at: www.govinfo.gov/content/pkg/FR-2010-05-07/pdf/2010-8159.pdf)

[R64] U.S. Environmental Protection Agency 2014a Control of air pollution from motor vehicles: tier 3 motor vehicle emission and fuel standard RIN: 2050-AQ86 (available at: www.epa.gov/greenvehicles/light-duty-vehicle-emissions#standards)

[R65] U.S. Environmental Protection Agency 2014b Control of air pollution from motor vehicles: tier 3 motor vehicle emission and fuel standard RIN: 2060-AQ86 (available at: www.govinfo.gov/content/pkg/FR-2014-04-28/pdf/2014-06954.pdf)

[R66] U.S. Environmental Protection Agency 2016 Light-duty vehicles and light-duty trucks: clean fuel fleet exhaust emission standards (available at: www.govinfo.gov/content/pkg/FR-2010-05-07/pdf/2010-8159.pdf)

[R67] U.S. Environmental Protection Agency 2021 Overview of EPA’s Motor Vehicle Emission Simulator (MOVES3) (available at: https://nepis.epa.gov/Exe/ZyPDF.cgi?Dockey=P1011KV2.pdf)

[R68] U.S. Environmental Protection Agency 2022a Sources of greenhouse gas emissions (available at: www.epa.gov/ghgemissions/sources-greenhouse-gas-emissions)

[R69] U.S. Environmental Protection Agency 2022b Smog, soot, and other air pollution from transportation (available at: www.epa.gov/transportation-air-pollution-and-climate-change/smog-soot-and-other-air-pollution-transportation)

[R70] U.S. Environmental Protection Agency 2022c Latest version of motor vehicle emission simulator (MOVES) (available at: www.epa.gov/moves/latest-version-motor-vehicle-emission-simulator-moves)

[R71] U.S. Environmental Protection Agency 2022e Overview of the cross-state air pollution rule (CSAPR) (available at: www.epa.gov/csapr/overview-cross-state-air-pollution-rule-csapr)

[R72] United Nations/Framework Convention on Climate Change 2015 Adoption of the Paris Agreement 21st Conf. Parties (United Nations)

[R73] Wolfram P, Weber S, Gillingham K and Hertwich EG 2021 Pricing indirect emissions accelerates low-carbon transition of US light vehicle sector Nat. Commun. 12 7121 34880225 10.1038/s41467-021-27247-y PMC8654946

[R74] World Bank Group 2020 State and trends of carbon pricing 2020

[R75] World Resources Institute 2022 Electric school bus initiative (available at: www.wri.org/initiatives/electric-school-bus-initiative)

[R76] Wu Y, Yang Z, Lin B, Liu H, Wang R, Zhou B and Hao J 2012 Energy consumption and CO2 emission impacts of vehicle electrification in three developed regions of China Energy Policy 48 537–50

[R77] Yuan M, Thellufsen JZ, Lund H and Liang Y 2021 The electrification of transportation in energy transition Energy 236 121564

[R78] ZEV Task Force 2022 Multi-state medium- and heavy-duty zero-emission vehicle action plan: a policy framework to eliminate harmful truck and bus emissions. Zero Emission Vehicle Task Force (available at: www.nescaum.org/documents/multi-state-medium-and-heavy-duty-zev-action-plan.pdf)

[R79] Zhao H, Burke A and Miller M 2013 Analysis of class 8 truck technologies for their fuel savings and economics Transp. Res. D 23 55–63

