# Supplementary material for: Evaluation of energy, carbon dioxide, and air emission implications of medium- and heavy-duty truck electrification in the United States using EPA’s regional TIMES energy systems model
Source: Environ Res Energy. Author manuscript; Available in PMC 2026 Jan 7. (PMC12180930; doi:10.1088/2753-3751/ad958b)
Supplement: SI [file NIHMS2052796-supplement-SI.docx]

Evaluation of energy, carbon dioxide, and air emission implications of medium- and heavy-duty truck electrification in the United States using EPA’s regional TIMES energy systems model

# Supplementary Information

## Note 1. US EPA Nine Region TIMES Database

TIMES is an optimization framework that aims to reach a partial equilibrium at every period for energy forms and services. Partial equilibrium is attained in such a way that the suppliers produce the same amount that customers are ready to purchase to meet their energy service demands. To reach the market equilibrium, TIMES assumes that; (1) There is a linear relationship between outputs and inputs of a technology; (2) Total cost is minimized over the entire horizon with perfect foresight; (3) The market price of any commodity in the model is the same as its marginal value attained by the model; (4) The whole market is competitive; and (5) Each agent in the system aims to maximize the profit.

| $Discounted total system cost=\sum_{r=1}^{R} \sum_{t=1}^{t=y} \left( 1+i \right)^{z\times\left( 1-t \right)}\times Annualized Cost \left( r,t \right)\times[(1+\left( 1+i \right)^{-1}+\left( 1+i \right)^{-2}+ \cdots+\left( 1+i \right)^{1-z}]$ | *Equation (1)* |
| --- | --- |

*where,*

*R = the number of regions.*

*y = the number of periods in the modeling horizon*

*z = the number of years in each period*

*i = the discount rate which is defined as a global parameter*

*Annualized cost (r,t) = the annualized total cost which belongs to region r for period t as presented in equation 2.*

A linear programming model is then set up to minimize total discounted system cost while satisfying the following set of constraints; (1) end-use demand satisfaction constraints; (2) capacity transfer constraints; (3) use of capacity; (4) balance for commodities (import/export); (5) electricity & heat balance; (6) peaking reserve constraints and (7) environmental constraints. TIMES has perfect foresight, and therefore is limited in making “real-world” decisions based on market conditions.

The TIMES energy economy is made up of producers and consumers of commodities such as energy carriers, materials, energy services, and emissions. By default, TIMES assumes competitive markets for all commodities, unless the modeler imposes regulatory or other constraints on some parts of the energy system, in which case the equilibrium is (partially) regulated. The result is a supply-demand equilibrium that maximizes the net total surplus (i.e. the sum of producers’ and consumers’ surpluses). TIMES may however depart from perfectly competitive market assumptions by the introduction of user-defined explicit constraints, such as limits to technological penetration, constraints on emissions, exogenous oil price, etc. Market imperfections can also be introduced in the form of taxes, subsidies and hurdle rates^[[1]](#footnote-2)^. In addition, model includes a general discount rate. The hurdle rates can be viewed as reflecting the perspective of an individual investor (descriptive approach), whereas the general discount rate reflects a social perspective (prescriptive approach). In our modeling we use hurdle rates (i.e., technology specific discount rates) as a parameter to influence technology penetration. High discount rates are justified by an implicit representation of financial constraints, information gaps or bounded rationality (other decision criteria) in the behavioral model of an investor. In addition, the hurdle rates would capture investment related risks such as policy-induced risks, country-specific risks, as well as technology-related risks. 10-15% hurdle rate is commonly established value for transportation sector^[[2]](#footnote-3)^.

Alongside the constraints mentioned above, the user can impose other “user-defined” constraints to shape the model in accordance with the real-life conditions for both demand and supply side of the model. The constraints are grouped for each end-use energy service demand. In this context, the constraints that are set for the building sector is composed of five sub-categories: lighting, space cooling, space heating, water heating, industrial facilities, and transportation sector constraints are also disaggregated into five sub-sectorial categories: bus, heavy-duty, light-duty, medium-duty and rail. On the other hand, the constraints that are imposed on the power sector have eight main categories according to the area of influence; the type of energy source, and the type of technology.

### Electric Sector Representation in EPAUS9rT

The Electric sector consists of conversion technologies that take in fuel resources and convert them to electricity for use in the end-use sectors. Power plant capacity is modeled as gigawatts (GW), and power plant costs are given in terms of dollars per GW. As electricity is produced, the output is converted to PJ of electricity through a conversion factor of 31.536 PJ/GW. The technologies represented range from fossil fuel conversion technologies to nuclear and renewable technologies. In addition to the regular emissions tracking, water consumption is characterized for all power plants in terms of million gallons per PJ of output electricity. The EPAUS9rT database represents the U.S. by census divisions to capture regional differences (Figure S1).

Coal Plant Retrofits. Several air pollution control retrofits are available to existing coal powered plants in the database. For NOx reductions the model can choose between Low NOx Burner (LNB), Selective Catalytic Reduction (SCR), Selective Non-Catalytic Reduction (SNCR), or a combination set-up. For SO2 reductions the model has can build flue gas desulfurization (FGD). PM10 retrofits include fabric filters (FFR), cyclones (CYC), ESP, and ESP upgrades.

New Electricity Conversion Technologies. Several electricity production technology options are included in the database including renewables such as solar and wind.

Renewables. The economics of wind and solar depend strongly on the quality of the available resources, which vary by region. Regionally-specific availability factors (AF) differentiate the cost-effectiveness of wind and solar across regions. Two broad solar technology types are modeled: solar photovoltaic (PV) and concentrating solar thermal (ST). Three types of solar PV technology are modeled: central electricity generation plants, distributed generation for residential application and distributed generation for commercial application. The technology representations for commercial and residential PV are found in the commercial and residential end-use sector workbooks. One concentrating central solar thermal technology is modeled.

Three wind technology types are available based on the class of wind resource (Class 4-6) and five cost categories (A-E). We utilized the The cost categories are based on the ease of access to wind resources for each wind class. The difference in capital cost from one category to another considers the cost of transmission interconnection. The total amount of wind development (i.e., installed wind capacity) was constrained by region per regional availability factors. In addition, inter-regional constraints on maximum installed wind capacity by cost category and wind class were applied at the national level using the NEMS input data from the AEO.

Both solar and wind cost and performance data is taken from National renewable Energy Laboratory Annual Technology Baseline^[[3]](#footnote-4)^.

### Time slices in the database and end use load curves

The way the MARKAL model represents the electric load curve impacts on the economic assessment of electric generating technologies. The fixed cost of competing alternative electric generating technologies can vary by a factor of 3^[[4]](#footnote-5)^. EPAUS9rT incorporates sixteen time slice to represent the load data. 16 timeslice assumes load is divided into seasons, day/night and around 1.5% of the load is allocated to peak slice. The data is calculated for three seasons, shoulder, summer and winter. In the EPAUS9rT, the intermediate season is further divided into two to represent Fall and Spring. The corresponding month walkthrough for the seasons are presented in Table S1. Further the daylight hours are divided into day AM and day PM. Table S2 presents assumed definitions for daylight AM/PM, night, and peak hours per seasons. The peak load time slice is allocated to weekdays, as most utilities consider peak pricing during weekdays except holidays between 4PM – 9 PM. The total peak time slice corresponds to around 1.5% of the year. Next using the number of hours calculated based on these assumptions per season, we come up with value corresponding fraction of year that falls into each time slice. The fractions and their definitions are listed in Table S3.

One additional note on the peaking units is that as far as the demand it can constitute up to 5% of generation. For instance, in 2021, peakers accounted for 3.1 percent of annual net electricity generation^[[5]](#footnote-6)^. In the model, the peaking units are represented, and the total generation is bound at 5%.

**Table S1: Month Definitions Load Slice Definitions**

| January | W |
| --- | --- |
| February | W |
| March | W |
| April | P |
| May | P |
| June | S |
| July | S |
| August | S |
| September | S |
| October | F |
| November | F |
| December | W |

**Table S2: Definition of daylight hours and peak hours per season**

|  | Day Hours | | # of Days | # of weekend days | # of hours | | | |
| --- | --- | --- | --- | --- | --- | --- | --- | --- |
|  |  |  |  |  | Peak | Night | DAM | DPM |
| Summer | 5:00 | 21:00 | 122 | 35 | 0 | 8 | 7 | 9 |
| Winter | 7:00 | 20:00 | 121 | 35 | 0 | 11 | 5 | 8 |
| Intermediate | 6:00 | 20:00 | 122 | 35 | 0 | 10 | 6 | 8 |
|  | Day Hours |  |  | # of weekdays |  |  |  |  |
| Summer | 5:00 | 21:00 |  | 87 | 2 | 8 | 7 | 7 |
| Winter | 7:00 | 20:00 |  | 86 | 1.2 | 11 | 5 | 6.8 |
| Intermediate | 6:00 | 20:00 |  | 87 | 1.2 | 10 | 6 | 6.8 |

**Table S3: Load Slice Definitions and Fraction of Year Per Time Slice**

|  | Description | Fraction of year |
| --- | --- | --- |
| I-DAM | Shoulder/Intermediate, Daytime, AM | 0.0836 |
| I-DPM | Shoulder/Intermediate, Daytime, PM | 0.0995 |
| I-N | Shoulder/Intermediate, Nighttime, AM | 0.1393 |
| I-P | Shoulder/Intermediate, Peak, AM | 0.0119 |
| S-DAM | Summer, Daytime, AM | 0.0975 |
| S-DPM | Summer, Daytime, PM | 0.1054 |
| S-N | Summer, Nighttime, AM | 0.1114 |
| S-P | Summer, Peak, AM | 0.0199 |
| W-DAM | Winter, Daytime, AM | 0.0691 |
| W-DPM | Winter, Daytime, PM | 0.0987 |
| W-N | Winter, Nighttime, AM | 0.1519 |
| W-P | Winter, Peak, AM | 0.0118 |

*Shoulder/Intermediate Season is further divided into FALL (F) and SPRING (P) in the EPAUS9rT database

*
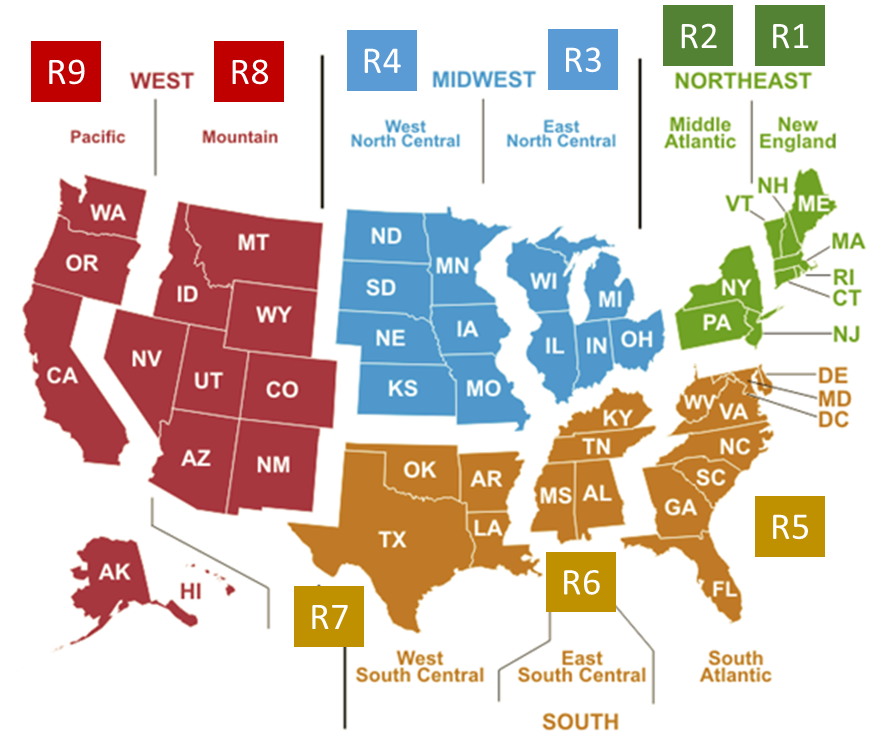
*

Figure S1. EPAUS9rT nine region coverage

## Note 2. Transportation Sector Updates to EPAUS9rT

The U.S. Environmental Protection Agency’s MOtor Vehicle Emission Simulator (MOVES) is an emissions modeling system that estimates greenhouse gas, criteria air pollutant, and air toxics emissions from on-road vehicles such as cars, trucks, and buses by considering federal emissions standards, fuels, temperatures, humidity, and emission control activities at the county to national scales (U.S. Environmental Protection Agency, 2021). The default transportation emission factors in the EPAUS9rT were national average values gathered using MOVES 2014. We updated the on-road transportation emission factors in EPAUS9rT by employing data from MOVES version 3. This latest version makes several improvements to better account for vehicle starts, long-haul truck hoteling, and off-network idling, and incorporates both the Heavy-Duty Greenhouse Gas Phase 2 rule and the Safer Affordable Fuel-Efficient Vehicles rule (U.S. Environmental Protection Agency, 2022a). The updated MOVES dataset had state-level emission factors which were then aggregated to the 9-region level to capture regional differences in transport emissions. Both datasets used the MOVES vehicle source classes with the difference that the original EPAUS9rT data broke down

light-duty vehicles into categories like compact car, large car, small SUV, etc., while the new dataset did not. This new dataset featured one emission factor per vehicle source class, region, pollutant, fuel, and year combination. Fuels included depend on the vehicle type, but may be gasoline, diesel fuel, compressed natural gas, ethanol 85, and/or electricity. Hydrogen fuel cell technology was not included since this study sought to isolate the impacts of electrification. Pollutants included criteria air pollutants and hazardous air pollutants; however, in this analysis we will be reporting on nitrogen oxides, particulate matter (2.5 scale), and sulfur dioxide. Tailpipe CO2 emissions were not derived from MOVES and were instead calculated endogenously within the model based on technology efficiency and fuel consumption.

The EPAUS9rT database includes existing (current stock of) technologies as well as future technology characterizations. It also contains a variety of hybrid technologies such as plug-in hybrid diesel trucks. MOVES’ fuel/vehicle combinations do not include some of these categories, so substitutions were necessary. For instance, light-duty vehicles fueled by compressed natural gas [CNG] were not represented in the MOVES data and so were assigned the emission factors for gasoline-fueled light-duty vehicles. Similarly, CNG-fueled heavy-duty short-haul trucks were assigned the emission factors for gasoline-fueled heavy-duty short-haul trucks. All transport technologies using liquified petroleum gas fuel were assigned corresponding gasoline emission factors. Battery electric technologies are not represented in MOVES for most applications where they have yet to experience widespread adoption, including heavy-duty trucks, so for these instances, all pollutant categories except PM were assigned emission factors of zero, while for PM, the sum of the brake wear and tire wear components of the corresponding diesel emission factors were assigned as the total PM emission factors for electric vehicles. The premise for this is that BEVs have no tailpipe emissions but produce PM from brakes and tires matching or exceeding that of conventional vehicles due to the significant weight conferred by their batteries (Emissions Analytics, 2020). Plug-in hybrid diesel vehicles were assigned corresponding diesel emission factors. Plug-in hybrid gasoline vehicles, only represented among light-duty vehicles, were assigned corresponding gasoline emission factors but were scaled by the proportion of the drive cycle using fossil fuel; those with an electric range of 10 miles used a factor of 0.82 and those with an electric range of 40 miles used a factor of 0.5, based on Short & Denholm (2006). Vehicles using B20 fuel, a mixture of 20% biodiesel and 80% diesel fuel, were assigned corresponding diesel emission factors with some pollutants scaled up or down according to data from the DOE’s Alternative Fuels Data Center [AFDC]: NOx was raised 2% compared to diesel levels while tailpipe PM and CO were lowered 13% compared to diesel levels (AFDC, 2022). In one case only, CNG-fueled commercial truck emission factors were drawn from MOVES-derived New York state-specific dataset since neither of the two datasets referred to above contained emission factors for this fuel/vehicle combination. Lastly, the MOVES3 dataset begins with the year 2015, so 2010 emission factors retained their existing EPAUS9rT MOVES2014 values.

The EPAUS9rT database uses different vehicle categories than MOVES (Table S4). The mapping used by this study was one-to-one between a MOVES source class and an EPAUS9rT category in most cases, while in other cases, two or more MOVES source classes were combined to form one EPAUS9rT category, like THL [heavy-duty long-haul truck]. For EPAUS9rT categories TM [medium-duty truck) and THS [heavy-duty short-haul truck], subsets of MOVES source classes were used based on regulatory class, which corresponds to vehicle weight. When multiple MOVES categories mapped to one EPAUS9rT category, emissions factors were calculated using a weighted average based on each MOVES category’s emission factor and vehicle-miles-traveled [VMT]. The new MOVES3 dataset contains VMT data, so this strategy could be used for THL, but the dataset does not have regulatory class-level detail, so more information was needed to calculate emission factors for TM and THS. In these cases, the New York State MOVES2014 dataset referred to above (Isik, et al., 2021) was employed since it does feature regulatory class-level detail. Therefore, which fractions of MOVES source classes 51 and 52 VMT corresponded to TM or THS in that dataset could be ascertained, and these fractions were then applied as weights to the MOVES3 source class-level emission factors. The result of this process was that THS final emission factors were a weighted average of the emission factors of source classes 51 (6%), 52 (41%) and 61 (53%), while TM final emission factors were a weighted average of those of source classes 51 (0%) and 52 (100%). THL used a weighted average of the emission factors of source classes 53 (4%) and 62 (96%).

**Table S4.** MOVES to TIMES mapping.

| MOVES classification | | EPAUS9rT classification* | | | | | | | |  |
| --- | --- | --- | --- | --- | --- | --- | --- | --- | --- | --- |
| Source class | Reg. Class** | TL | TL*** | TC | TBT | TBS | TM | THS | THL | |
| 21 (passenger car) | - | X |  |  |  |  |  |  |  | |
| 31 (passenger truck) | - |  | X |  |  |  |  |  |  | |
| 32 (light commercial truck) | - |  |  | X |  |  |  |  |  | |
| 42 (transit buses) | - |  |  |  | X |  |  |  |  | |
| 43 (school buses) | - |  |  |  |  | X |  |  |  | |
| 51 (refuse trucks) | 41 |  |  |  |  |  | X |  |  | |
|  | 42 |  |  |  |  |  | X |  |  | |
|  | 46 |  |  |  |  |  |  | X |  | |
|  | 47 |  |  |  |  |  |  | X |  | |
| 52 (single unit short haul truck) | 41 |  |  |  |  |  | X |  |  | |
|  | 42 |  |  |  |  |  | X |  |  | |
|  | 46 |  |  |  |  |  |  | X |  | |
|  | 47 |  |  |  |  |  |  | X |  | |
| 53 (single unit long haul truck) | - |  |  |  |  |  |  |  | X | |
| 61 (combination short haul truck) | - |  |  |  |  |  |  | X |  | |
| 62 (combination long haul truck) | - |  |  |  |  |  |  |  | X | |

*EPAUS9rT categories: TL=light duty, TC=commercial truck, TBT=transit bus, TBS=school bus, TM=medium-duty truck, THS=heavy-duty short-haul truck, THL=heavy-duty long-haul truck. **Regulatory classes shown only when a subset is used. 41= Class 2b Trucks with 2 Axles and at least 6 Tires or Class 3 Trucks (8,500 lbs < GVWR <= 14,000 lbs), 42= Class 4 and 5 Trucks (14,00 lbs < GVWR <= 19,500 lbs), 46= Class 6 and 7 Trucks (19,500 lbs < GVWR < =33,000 lbs), 47= Class 8a and 8b Trucks (GVWR > 33,000 lbs). ***TL pickup trucks categorized under regulatory class 41. Source: (U.S. Environmental Protection Agency, 2016).

# Transportation Demands

Annual demands by region for the transportation sector were based on the Energy Information Administration’s Annual Energy Outlook [AEO] (EIA, 2022). Heavy-duty demands for the years 2010 and 2011 used the 2013 AEO, the year 2015 used the 2017 AEO, and all subsequent years used the 2020 AEO. The AEO provides data for the total heavy-duty freight sector, so the vehicle-miles-traveled information from the MOVES3 data was used to estimate fractions of this demand to attribute to short-haul and long-haul heavy-duty trucking. This process assigned slightly different fractions of the overall AEO heavy-duty demand to THS or THL categories in each region because each region has a slightly different ratio of THS to THL VMT. For most regions and years, this method apportioned roughly one-third of the heavy-duty demand to short-haul trucking and two-thirds to long-haul trucking. Light duty demands for each year were based on the 2018 AEO. While TIMES can represent elastic demand, all demands were fixed in this model. Demands are calculated by using national level energy consumption from the AEO which are regionalized using data collected from several different sources.

**Table S5.** Light Duty Vehicle Fuel and Technology Combinations

|  |  | **Car Class** | | | | | | |
| --- | --- | --- | --- | --- | --- | --- | --- | --- |
| Fuel | Technology | **Mini-Compact** | **Compact** | **Full-size** | **Minivan** | **Pickup** | **Small SUV** | **Large SUV** |
| Gasoline | Conventional | X | X | X | X | X | X | X |
|  | Advanced | X | X | X | X | X | X | X |
|  | Hybrid |  | X | X | X | X | X | X |
|  | Plug-in Hybrid (20 miles per charge) |  | X | X | X | X | X | X |
|  | Plug-in Hybrid (40 miles per charge) |  | X | X | X | X | X | X |
| Diesel | Conventional |  | X | X | X | X | X | X |
|  | Hybrid |  | X | X | X |  | X | X |
| E85 | Flexfuel |  | X | X | X | X | X | X |
|  | Advanced |  | X | X | X | X | X | X |
|  | Hybrid |  | X | X | X | X | X | X |
|  | Plug-in Hybrid (20 miles per charge) |  | X | X | X | X | X | X |
|  | Plug-in Hybrid (40 miles per charge) |  | X | X | X | X | X | X |
| CNG | Conventional |  | X | X | X | X |  |  |
|  | Flexfuel |  | X | X | X | X |  |  |
| LPG | Conventional |  |  |  | X | X |  |  |
|  | Flexfuel |  | X | X | X | X |  |  |
| Electric | 100 mile range | X | X | X | X | X | X | X |
|  | 200 mile range | X | X | X | X | X | X | X |

**Table S6:** Medium – and Heavy - Duty Transportation Demands Description and Units

| Name | Description | Units | Unit Description |
| --- | --- | --- | --- |
| TA | Domestic Air Transport | bn-pass-miles | billion passenger miles |
| TBS | Bus - School | bn-vmt | billion vehicle miles traveled |
| TBT | Bus - Transit | bn-vmt | billion vehicle miles traveled |
| TC | Commercial Trucks (Class 2b) | bn-vmt | billion vehicle miles traveled |
| TM | Medium Duty Trucks (Class 3-6) | bn-vmt | billion vehicle miles traveled |
| THS | Short Haul Heavy Duty Trucks (Class 7-8) | bn-vmt | billion vehicle miles traveled |
| THL | Long Haul Heavy Duty Trucks (Class 7-8) | bn-vmt | billion vehicle miles traveled |
| TRF | Freight Rail | bn-t-m | billion ton miles |
| TRP | Passenger Rail | bn-pass-miles | billion passenger miles |
| TS | Shipping (Marine) | bn-t-m | billion ton miles |

**Table S7:** Medium – and Heavy – Duty Transportation Demands

| **Region 1** | **2010** | **2011** | **2015** | **2020** | **2025** | **2030** | **2035** | **2040** | **2045** | **2050** | **2055** |
| --- | --- | --- | --- | --- | --- | --- | --- | --- | --- | --- | --- |
| TA | 33.36 | 32.81 | 36.39 | 41.53 | 44.48 | 48.38 | 52.76 | 57.31 | 62.45 | 68.36 | 68.36 |
| TBT | 0.20 | 0.19 | 0.20 | 0.19 | 0.20 | 0.21 | 0.21 | 0.21 | 0.21 | 0.21 | 0.21 |
| TBS | 0.27 | 0.26 | 0.28 | 0.24 | 0.25 | 0.25 | 0.26 | 0.27 | 0.27 | 0.28 | 0.28 |
| TM | 5.19 | 5.40 | 5.24 | 5.86 | 6.38 | 7.06 | 7.92 | 8.80 | 9.77 | 10.88 | 10.88 |
| THS | 2.37 | 2.39 | 2.38 | 2.52 | 2.73 | 2.90 | 3.05 | 3.18 | 3.34 | 3.49 | 3.49 |
| THL | 4.68 | 4.72 | 4.70 | 5.03 | 5.12 | 5.19 | 5.25 | 5.26 | 5.34 | 5.35 | 5.35 |
| TRF | 6.48 | 6.38 | 7.58 | 7.04 | 6.80 | 7.05 | 7.23 | 7.27 | 7.52 | 7.83 | 7.83 |
| TRP | 2.42 | 2.32 | 2.32 | 2.73 | 2.89 | 3.04 | 3.18 | 3.32 | 3.44 | 3.57 | 3.70 |
| TS | 18.59 | 18.81 | 18.13 | 14.91 | 13.24 | 11.59 | 10.86 | 10.10 | 9.88 | 9.61 | 9.61 |
| TC | 10.72 | 11.92 | 12.54 | 13.21 | 13.90 | 14.80 | 15.78 | 16.72 | 17.82 | 18.95 | 18.95 |
|  |  |  |  |  |  |  |  |  |  |  |  |
|  |  |  |  |  |  |  |  |  |  |  |  |
|  |  |  |  |  |  |  |  |  |  |  |  |
| **Region 2** | **2010** | **2011** | **2015** | **2020** | **2025** | **2030** | **2035** | **2040** | **2045** | **2050** | **2055** |
| TA | 111.36 | 109.54 | 121.49 | 138.65 | 148.48 | 161.50 | 176.13 | 191.34 | 208.47 | 228.22 | 228.22 |
| TBT | 0.92 | 0.90 | 0.94 | 0.91 | 0.94 | 0.97 | 0.99 | 1.01 | 1.01 | 1.01 | 1.01 |
| TBS | 1.26 | 1.24 | 1.34 | 1.14 | 1.17 | 1.20 | 1.23 | 1.25 | 1.28 | 1.31 | 1.34 |
| TM | 14.53 | 15.13 | 14.67 | 16.40 | 17.87 | 19.76 | 22.18 | 24.63 | 27.35 | 30.47 | 30.47 |
| THS | 4.00 | 4.04 | 4.02 | 4.25 | 4.61 | 4.90 | 5.17 | 5.39 | 5.66 | 5.91 | 5.91 |
| THL | 8.33 | 8.41 | 8.37 | 8.97 | 9.12 | 9.25 | 9.36 | 9.39 | 9.53 | 9.55 | 9.55 |
| TRF | 63.41 | 62.42 | 74.14 | 68.90 | 66.51 | 68.96 | 70.72 | 71.15 | 73.59 | 76.57 | 76.57 |
| TRP | 19.43 | 18.65 | 18.65 | 21.94 | 23.20 | 24.41 | 25.59 | 26.67 | 27.67 | 28.68 | 29.72 |
| TS | 5.99 | 6.07 | 5.85 | 4.81 | 4.27 | 3.74 | 3.50 | 3.26 | 3.18 | 3.10 | 3.10 |
| TC | 5.71 | 6.34 | 6.68 | 7.03 | 7.40 | 7.88 | 8.40 | 8.90 | 9.48 | 10.09 | 10.09 |
|  |  |  |  |  |  |  |  |  |  |  |  |
|  |  |  |  |  |  |  |  |  |  |  |  |
|  |  |  |  |  |  |  |  |  |  |  |  |
| **Region 3** | **2010** | **2011** | **2015** | **2020** | **2025** | **2030** | **2035** | **2040** | **2045** | **2050** | **2055** |
| TA | 121.35 | 119.36 | 132.38 | 151.08 | 161.80 | 175.98 | 191.92 | 208.50 | 227.17 | 248.69 | 248.69 |
| TBT | 0.65 | 0.63 | 0.66 | 0.64 | 0.66 | 0.68 | 0.69 | 0.71 | 0.71 | 0.71 | 0.71 |
| TBS | 0.88 | 0.87 | 0.94 | 0.80 | 0.82 | 0.84 | 0.86 | 0.88 | 0.90 | 0.92 | 0.94 |
| TM | 14.53 | 15.13 | 14.67 | 16.40 | 17.87 | 19.76 | 22.18 | 24.63 | 27.35 | 30.47 | 30.47 |
| THS | 7.47 | 7.54 | 7.51 | 7.95 | 8.61 | 9.15 | 9.65 | 10.06 | 10.57 | 11.03 | 11.03 |
| THL | 15.43 | 15.58 | 15.50 | 16.61 | 16.89 | 17.13 | 17.34 | 17.39 | 17.65 | 17.69 | 17.69 |
| TRF | 249.84 | 245.94 | 292.11 | 271.48 | 262.05 | 271.71 | 278.65 | 280.33 | 289.94 | 301.70 | 301.70 |
| TRP | 3.00 | 2.88 | 2.88 | 3.38 | 3.58 | 3.76 | 3.95 | 4.11 | 4.27 | 4.42 | 4.58 |
| TS | 12.75 | 12.90 | 12.43 | 10.22 | 9.08 | 7.95 | 7.45 | 6.93 | 6.77 | 6.59 | 6.59 |
| TC | 8.83 | 9.81 | 10.33 | 10.88 | 11.44 | 12.19 | 13.00 | 13.77 | 14.67 | 15.61 | 15.61 |
|  |  |  |  |  |  |  |  |  |  |  |  |
|  |  |  |  |  |  |  |  |  |  |  |  |
|  |  |  |  |  |  |  |  |  |  |  |  |
| **Region 4** | **2010** | **2011** | **2015** | **2020** | **2025** | **2030** | **2035** | **2040** | **2045** | **2050** | **2055** |
| TA | 50.34 | 49.51 | 54.91 | 62.67 | 67.12 | 73.00 | 79.61 | 86.49 | 94.23 | 103.16 | 103.16 |
| TBT | 0.29 | 0.28 | 0.29 | 0.28 | 0.29 | 0.30 | 0.31 | 0.31 | 0.31 | 0.31 | 0.31 |
| TBS | 0.39 | 0.39 | 0.41 | 0.35 | 0.36 | 0.37 | 0.38 | 0.39 | 0.40 | 0.40 | 0.41 |
| TM | 9.34 | 9.73 | 9.43 | 10.54 | 11.49 | 12.70 | 14.26 | 15.83 | 17.58 | 19.59 | 19.59 |
| THS | 6.87 | 6.94 | 6.91 | 7.31 | 7.93 | 8.42 | 8.88 | 9.26 | 9.72 | 10.15 | 10.15 |
| THL | 14.26 | 14.40 | 14.33 | 15.35 | 15.61 | 15.84 | 16.04 | 16.09 | 16.33 | 16.36 | 16.36 |
| TRF | 234.82 | 231.15 | 274.55 | 255.16 | 246.30 | 255.37 | 261.90 | 263.48 | 272.51 | 283.56 | 283.56 |
| TRP | 0.43 | 0.42 | 0.42 | 0.49 | 0.52 | 0.55 | 0.57 | 0.60 | 0.62 | 0.64 | 0.66 |
| TS | 80.64 | 81.63 | 78.67 | 64.67 | 57.45 | 50.28 | 47.12 | 43.83 | 42.86 | 41.71 | 41.71 |
| TC | 8.36 | 9.29 | 9.78 | 10.30 | 10.83 | 11.54 | 12.30 | 13.04 | 13.89 | 14.77 | 14.77 |
|  |  |  |  |  |  |  |  |  |  |  |  |
|  |  |  |  |  |  |  |  |  |  |  |  |
|  |  |  |  |  |  |  |  |  |  |  |  |
| **Region 5** | **2010** | **2011** | **2015** | **2020** | **2025** | **2030** | **2035** | **2040** | **2045** | **2050** | **2055** |
| TA | 235.51 | 231.64 | 256.92 | 293.21 | 314.01 | 341.53 | 372.47 | 404.64 | 440.88 | 482.64 | 482.64 |
| TBT | 0.61 | 0.60 | 0.62 | 0.60 | 0.62 | 0.64 | 0.66 | 0.67 | 0.67 | 0.67 | 0.67 |
| TBS | 0.83 | 0.82 | 0.88 | 0.75 | 0.77 | 0.79 | 0.81 | 0.83 | 0.84 | 0.86 | 0.89 |
| TM | 19.72 | 20.54 | 19.91 | 22.26 | 24.26 | 26.82 | 30.10 | 33.42 | 37.12 | 41.36 | 41.36 |
| THS | 7.90 | 7.97 | 7.94 | 8.40 | 9.12 | 9.69 | 10.21 | 10.65 | 11.18 | 11.68 | 11.68 |
| THL | 16.76 | 16.92 | 16.84 | 18.04 | 18.35 | 18.62 | 18.86 | 18.92 | 19.21 | 19.25 | 19.25 |
| TRF | 205.57 | 202.35 | 240.35 | 223.37 | 215.61 | 223.56 | 229.27 | 230.65 | 238.56 | 248.23 | 248.23 |
| TRP | 3.32 | 3.19 | 3.19 | 3.75 | 3.97 | 4.18 | 4.38 | 4.56 | 4.73 | 4.91 | 5.09 |
| TS | 38.29 | 38.76 | 37.35 | 30.71 | 27.28 | 23.87 | 22.38 | 20.81 | 20.35 | 19.81 | 19.81 |
| TC | 15.81 | 17.56 | 18.49 | 19.47 | 20.48 | 21.82 | 23.27 | 24.65 | 26.26 | 27.93 | 27.93 |
|  |  |  |  |  |  |  |  |  |  |  |  |
|  |  |  |  |  |  |  |  |  |  |  |  |
|  |  |  |  |  |  |  |  |  |  |  |  |
|  |  |  |  |  |  |  |  |  |  |  |  |
| **Region 6** | **2010** | **2011** | **2015** | **2020** | **2025** | **2030** | **2035** | **2040** | **2045** | **2050** | **2055** |
| TA | 40.25 | 39.59 | 43.91 | 50.11 | 53.67 | 58.37 | 63.66 | 69.16 | 75.35 | 82.49 | 82.49 |
| TBT | 0.21 | 0.21 | 0.21 | 0.21 | 0.22 | 0.22 | 0.23 | 0.23 | 0.23 | 0.23 | 0.23 |
| TBS | 0.29 | 0.28 | 0.31 | 0.26 | 0.27 | 0.27 | 0.28 | 0.29 | 0.29 | 0.30 | 0.31 |
| TM | 5.19 | 5.40 | 5.24 | 5.86 | 6.38 | 7.06 | 7.92 | 8.80 | 9.77 | 10.88 | 10.88 |
| THS | 5.88 | 5.94 | 5.91 | 6.26 | 6.81 | 7.24 | 7.63 | 7.96 | 8.36 | 8.73 | 8.73 |
| THL | 13.49 | 13.62 | 13.56 | 14.51 | 14.78 | 15.00 | 15.21 | 15.27 | 15.52 | 15.57 | 15.57 |
| TRF | 108.16 | 106.47 | 126.46 | 117.53 | 113.45 | 117.63 | 120.63 | 121.36 | 125.52 | 130.61 | 130.61 |
| TRP | 0.24 | 0.23 | 0.23 | 0.27 | 0.28 | 0.30 | 0.31 | 0.32 | 0.34 | 0.35 | 0.36 |
| TS | 12.24 | 12.39 | 11.94 | 9.82 | 8.72 | 7.63 | 7.15 | 6.65 | 6.50 | 6.33 | 6.33 |
| TC | 6.31 | 7.01 | 7.38 | 7.77 | 8.17 | 8.71 | 9.28 | 9.84 | 10.48 | 11.15 | 11.15 |
|  |  |  |  |  |  |  |  |  |  |  |  |
|  |  |  |  |  |  |  |  |  |  |  |  |
|  |  |  |  |  |  |  |  |  |  |  |  |
|  |  |  |  |  |  |  |  |  |  |  |  |
| **Region 7** | **2010** | **2011** | **2015** | **2020** | **2025** | **2030** | **2035** | **2040** | **2045** | **2050** | **2055** |
| TA | 105.67 | 103.94 | 115.28 | 131.56 | 140.89 | 153.24 | 167.12 | 181.55 | 197.82 | 216.55 | 216.55 |
| TBT | 0.37 | 0.36 | 0.38 | 0.37 | 0.38 | 0.39 | 0.40 | 0.40 | 0.40 | 0.40 | 0.40 |
| TBS | 0.50 | 0.50 | 0.54 | 0.46 | 0.47 | 0.48 | 0.49 | 0.50 | 0.51 | 0.52 | 0.54 |
| TM | 8.30 | 8.65 | 8.38 | 9.37 | 10.21 | 11.29 | 12.68 | 14.07 | 15.63 | 17.41 | 17.41 |
| THS | 8.73 | 8.81 | 8.77 | 9.29 | 10.09 | 10.73 | 11.31 | 11.80 | 12.39 | 12.94 | 12.94 |
| THL | 19.45 | 19.64 | 19.55 | 20.93 | 21.30 | 21.62 | 21.91 | 21.99 | 22.34 | 22.40 | 22.40 |
| TRF | 138.99 | 136.82 | 162.51 | 151.03 | 145.79 | 151.16 | 155.02 | 155.96 | 161.30 | 167.85 | 167.85 |
| TRP | 0.44 | 0.43 | 0.43 | 0.50 | 0.53 | 0.56 | 0.58 | 0.61 | 0.63 | 0.66 | 0.68 |
| TS | 246.90 | 249.93 | 240.86 | 198.01 | 175.90 | 153.94 | 144.28 | 134.18 | 131.21 | 127.71 | 127.71 |
| TC | 6.33 | 7.04 | 7.41 | 7.80 | 8.21 | 8.74 | 9.32 | 9.88 | 10.52 | 11.19 | 11.19 |
|  |  |  |  |  |  |  |  |  |  |  |  |
|  |  |  |  |  |  |  |  |  |  |  |  |
| **Region 8** | **2010** | **2011** | **2015** | **2020** | **2025** | **2030** | **2035** | **2040** | **2045** | **2050** | **2055** |
| TA | 120.65 | 118.67 | 131.62 | 150.21 | 160.87 | 174.97 | 190.82 | 207.29 | 225.86 | 247.25 | 247.25 |
| TBT | 0.23 | 0.22 | 0.23 | 0.23 | 0.23 | 0.24 | 0.25 | 0.25 | 0.25 | 0.25 | 0.25 |
| TBS | 0.31 | 0.31 | 0.33 | 0.28 | 0.29 | 0.30 | 0.30 | 0.31 | 0.32 | 0.32 | 0.33 |
| TM | 8.30 | 8.65 | 8.38 | 9.37 | 10.21 | 11.29 | 12.68 | 14.07 | 15.63 | 17.41 | 17.41 |
| THS | 4.33 | 4.37 | 4.35 | 4.61 | 5.00 | 5.32 | 5.61 | 5.86 | 6.15 | 6.43 | 6.43 |
| THL | 9.76 | 9.85 | 9.81 | 10.50 | 10.69 | 10.85 | 11.00 | 11.04 | 11.21 | 11.24 | 11.24 |
| TRF | 487.19 | 479.57 | 569.62 | 529.39 | 511.00 | 529.83 | 543.37 | 546.64 | 565.39 | 588.32 | 588.32 |
| TRP | 0.50 | 0.48 | 0.48 | 0.56 | 0.59 | 0.62 | 0.65 | 0.68 | 0.71 | 0.73 | 0.76 |
| TS | 22.55 | 22.82 | 22.00 | 18.08 | 16.06 | 14.06 | 13.18 | 12.25 | 11.98 | 11.66 | 11.66 |
| TC | 10.15 | 11.28 | 11.88 | 12.51 | 13.16 | 14.02 | 14.95 | 15.84 | 16.87 | 17.94 | 17.94 |
|  |  |  |  |  |  |  |  |  |  |  |  |
|  |  |  |  |  |  |  |  |  |  |  |  |
| **Region 9** | **2010** | **2011** | **2015** | **2020** | **2025** | **2030** | **2035** | **2040** | **2045** | **2050** | **2055** |
| TA | 180.28 | 177.32 | 196.67 | 224.45 | 240.37 | 261.43 | 285.12 | 309.74 | 337.48 | 369.45 | 369.45 |
| TBT | 0.61 | 0.60 | 0.62 | 0.61 | 0.63 | 0.64 | 0.66 | 0.67 | 0.67 | 0.67 | 0.67 |
| TBS | 0.84 | 0.83 | 0.89 | 0.76 | 0.77 | 0.80 | 0.82 | 0.83 | 0.85 | 0.87 | 0.89 |
| TM | 18.69 | 19.46 | 18.86 | 21.09 | 22.98 | 25.41 | 28.52 | 31.67 | 35.17 | 39.18 | 39.18 |
| THS | 8.57 | 8.66 | 8.62 | 9.12 | 9.89 | 10.51 | 11.07 | 11.55 | 12.13 | 12.67 | 12.67 |
| THL | 17.84 | 18.02 | 17.93 | 19.21 | 19.54 | 19.82 | 20.07 | 20.13 | 20.43 | 20.47 | 20.47 |
| TRF | 86.65 | 85.30 | 101.32 | 94.16 | 90.89 | 94.24 | 96.65 | 97.23 | 100.56 | 104.64 | 104.64 |
| TRP | 3.87 | 3.72 | 3.72 | 4.37 | 4.62 | 4.86 | 5.10 | 5.32 | 5.51 | 5.71 | 5.92 |
| TS | 69.93 | 70.79 | 68.21 | 56.08 | 49.82 | 43.60 | 40.86 | 38.00 | 37.16 | 36.17 | 36.17 |
| TC | 9.23 | 10.25 | 10.79 | 11.37 | 11.96 | 12.74 | 13.58 | 14.39 | 15.33 | 16.31 | 16.31 |


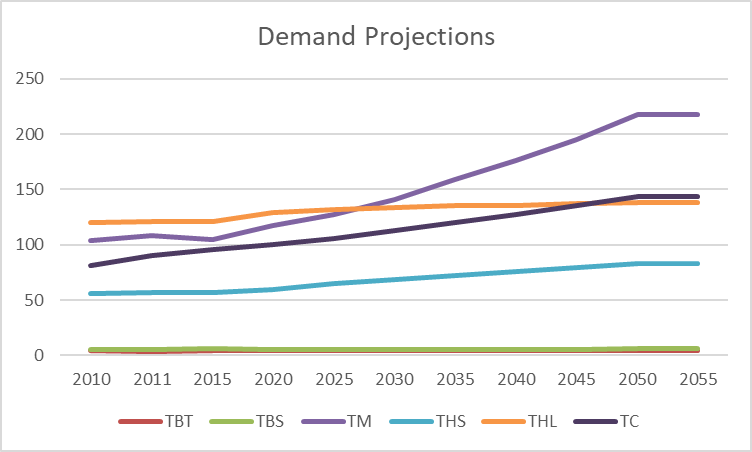


**Table S8:** Cost assumptions for medium- and heavy-duty vehicles (Mid-Vehicle Scenario from NREL ATB)

| Technology Description | Start Year | Lifetime | Investment Cost, 2005 $US M/demand unit | | | | | | | | |
| --- | --- | --- | --- | --- | --- | --- | --- | --- | --- | --- | --- |
|  |  |  | 2011 | 2015 | 2020 | 2025 | 2030 | 2035 | 2040 | 2045 | 2050 |
| Air Jet, Passenger | 2011 | 30 | 815 | 815 | 809 | 803 | 797 | 791 | 785 |  | 774 |
| Air Jet, Passenger, EV | 2030 | 30 |  |  |  |  | 1214 |  |  |  | 971 |
| Bus Transit, Conventional Diesel | 2011 | 12 | 2752 | 2752 | 2752 | 2769 | 2786 | 2803 | 2820 | 2838 | 2872 |
| Bus Transit, Conventional GSL | 2011 | 12 | 2972 | 2972 | 2972 | 2990 | 3009 | 3028 | 3046 | 3065 | 3102 |
| Bus Transit, Conventional EV | 2015 | 12 | 6140 | 6140 | 6140 | 5469 | 4506 | 3990 | 3784 | 3560 | 3354 |
| Bus Transit, Hydrogen Fuel Cell | 2011 | 12 | 6002 | 6002 | 6002 | 4695 | 4179 | 3904 | 3801 | 3698 | 3594 |
| Bust Transit, Conventional DHEV | 2015 | 12 | 2958 | 2958 | 2958 | 2975 | 2975 | 2992 | 3010 | 3027 | 3044 |
| Bus Transit, Conventional B20 | 2011 | 12 | 2752 | 2752 | 2752 | 2769 | 2786 | 2803 | 2820 | 2838 | 2872 |
| Bus Transit, Conventional CNG | 2011 | 12 | 2752 | 2752 | 2752 | 2769 | 2786 | 2803 | 2820 | 2838 | 2872 |
| Bus School, Conventional Diesel | 2011 | 12 | 9681 | 9681 | 4277 | 4277 | 4308 | 4303 | 4313 | 4324 | 4329 |
| Bus School, Conventional GSL | 2011 | 12 | 10456 | 10456 | 4277 | 4277 | 4308 | 4303 | 4313 | 4324 | 4329 |
| Bus School, Conventional EV | 2015 | 12 | 29564 | 29564 | 5521 | 5159 | 4700 | 4453 | 4334 | 4220 | 4102 |
| Bust School, Conventional DHEV | 2015 | 12 | 16135 | 16135 | 8049 | 6449 | 6088 | 5779 | 5624 | 5521 | 5366 |
| Bus School, Conventional B20 | 2011 | 12 | 9681 | 9681 | 4783 | 4736 | 4721 | 4685 | 4674 | 4669 | 4659 |
| Bus School, Conventional LPG | 2011 | 12 | 9991 | 9991 | 4277 | 4277 | 4308 | 4303 | 4313 | 4324 | 4329 |
| Bus School, Conventional CNG | 2011 | 12 | 12233 | 12233 | 4277 | 4277 | 4308 | 4303 | 4313 | 4324 | 4329 |
| Commercial Truck, Conventional Diesel | 2011 | 15.5 | 1457 | 1457 | 4277 | 4277 | 4308 | 4303 | 4313 | 4324 | 4329 |
| Commercial Truck, Conventional Gasoline | 2011 | 15.5 | 1061 | 1061 | 4277 | 4277 | 4308 | 4303 | 4313 | 4324 | 4329 |
| Commercial Truck, ELC | 2020 | 15.5 | 2099 | 2099 | 5521 | 5159 | 4700 | 4453 | 4334 | 4220 | 4102 |
| Commercial Truck, Diesel HEV | 2025 | 15.5 | 1544 | 1544 | 8049 | 6449 | 6088 | 5779 | 5624 | 5521 | 5366 |
| Commercial Truck, Conventional B20 | 2015 | 15.5 | 1457 | 1457 | 4783 | 4736 | 4721 | 4685 | 4674 | 4669 | 4659 |
| Commercial Truck, Conventional LPG | 2011 | 15.5 | 1355 | 1355 | 4277 | 4277 | 4308 | 4303 | 4313 | 4324 | 4329 |
| Commercial Truck, Conventional CNG | 2011 | 15.5 | 1610 | 1610 | 4277 | 4277 | 4308 | 4303 | 4313 | 4324 | 4329 |
| Commercial Truck, Conventional E85 | 2011 | 15.5 | 1100 | 1100 | 4277 | 4277 | 4308 | 4303 | 4313 | 4324 | 4329 |
| Medium Duty Truck, Conventional Diesel | 2011 | 19 | 3139 | 3139 | 4277 | 4277 | 4308 | 4303 | 4313 | 4324 | 4329 |
| Medium Duty Truck, Conventional Gasoline | 2011 | 19 | 3139 | 3139 | 4619 | 4619 | 4653 | 4647 | 4658 | 4669 | 4675 |
| Medium Duty Truck, ELC | 2020 | 19 | 7414 | 7414 | 5521 | 5159 | 4700 | 4453 | 4334 | 4220 | 4102 |
| Medium Duty Truck, Diesel HEV | 2020 | 19 | 3987 | 3987 | 4783 | 4736 | 4721 | 4685 | 4674 | 4669 | 4659 |
| Medium Duty Truck, Conventional B20 | 2011 | 19 | 3139 | 3139 | 4277 | 4277 | 4308 | 4303 | 4313 | 4324 | 4329 |
| Medium Duty Truck, Conventional LPG | 2011 | 19 | 3855 | 3855 | 4277 | 4277 | 4308 | 4303 | 4313 | 4324 | 4329 |
| Medium Duty Truck, Conventional CNG | 2011 | 19 | 5835 | 5835 | 4277 | 4277 | 4308 | 4303 | 4313 | 4324 | 4329 |
| Heavy Truck Long Haul, Conv. Diesel | 2011 | 19 | 683 | 683 | 1905 | 1917 | 1929 | 1941 | 1953 | 1965 | 1988 |
| Heavy Truck Long Haul, ELC | 2025 | 19 | 3870 | 3870 | 4251 | 3786 | 3119 | 2762 | 2619 | 2465 | 2322 |
| Heavy Truck Long Haul, Diesel HEV | 2020 | 19 | 2048 | 2048 | 2048 | 2060 | 2060 | 2072 | 2084 | 2096 | 2107 |
| Heavy Truck Long Haul, Conventional B20 | 2011 | 19 | 1905 | 1905 | 1905 | 1917 | 1929 | 1941 | 1953 | 1965 | 1988 |
| Heavy Truck Short Haul, Conv. Diesel | 2011 | 19 | 4953 | 4953 | 4953 | 4984 | 5015 | 5046 | 5077 | 5108 | 5170 |
| Heavy Truck Short Haul, ELC | 2025 | 19 | 11052 | 11052 | 11052 | 9844 | 8111 | 7182 | 6810 | 6408 | 6037 |
| Heavy Truck Short Haul, Diesel HEV | 2020 | 19 | 5325 | 5325 | 5325 | 5355 | 5355 | 5386 | 5417 | 5448 | 5479 |
| Heavy Truck Short Haul, Conventional B20 | 2011 | 19 | 4953 | 4953 | 4953 | 4984 | 5015 | 5046 | 5077 | 5108 | 5170 |
| Heavy Truck Short Haul, Conventional LPG | 2011 | 19 | 4953 | 4953 | 4953 | 4984 | 5015 | 5046 | 5077 | 5108 | 5170 |
| Heavy Truck Short Haul, Conventional CNG | 2011 | 19 | 4953 | 4953 | 4953 | 4984 | 5015 | 5046 | 5077 | 5108 | 5170 |

**Table S9:** Performance assumptions for medium- and heavy-duty vehicles (Mid-Vehicle scenario from NREL ATB)

| Technology Description | Start Year |  | Efficiency, demand unit/PJ | | | | | | | | | O&M Cost, 2005 $US M/demand unit | Technology-specific discount rate | | |
| --- | --- | --- | --- | --- | --- | --- | --- | --- | --- | --- | --- | --- | --- | --- | --- |
|  |  |  | 2011 | 2015 | 2020 | 2025 | 2030 | 2035 | 2040 | 2045 | 2050 |  | All years | 2025 | 2035 |
| Air Jet, Passenger | 2011 | 30 | 0.451 |  |  |  | 0.519 |  |  |  | 0.587 | 7.46 | 18% |  |  |
| Air Jet, Passenger, EV | 2030 | 30 |  |  |  |  | 1.038 |  |  |  | 1.101 | 9.65 | 24% |  |  |
| Bus Transit, Conventional Diesel | 2011 | 12 | 0.061 | 0.061 | 0.061 | 0.067 | 0.074 | 0.081 | 0.084 | 0.088 | 0.091 | 385 | 10% |  |  |
| Bus Transit, Conventional GSL | 2011 | 12 | 0.068 | 0.068 | 0.068 | 0.074 | 0.082 | 0.089 | 0.093 | 0.097 | 0.100 | 385 | 10% |  |  |
| Bus Transit, Conventional EV | 2015 | 12 | 0.107 | 0.107 | 0.107 | 0.118 | 0.132 | 0.145 | 0.151 | 0.157 | 0.163 | 248 |  | 15% | 10% |
| Bus Transit, Hydrogen Fuel Cell | 2015 | 12 | 0.057 | 0.057 | 0.057 | 0.065 | 0.074 | 0.082 | 0.086 | 0.091 | 0.096 | 248 |  | 15% | 10% |
| Bust Transit, Conventional DHEV | 2011 | 12 | 0.048 | 0.048 | 0.048 | 0.053 | 0.059 | 0.065 | 0.068 | 0.071 | 0.073 | 385 | 10% |  |  |
| Bus Transit, Conventional B20 | 2011 | 12 | 0.061 | 0.061 | 0.061 | 0.067 | 0.074 | 0.081 | 0.084 | 0.088 | 0.091 | 385 |  | 15% | 10% |
| Bus Transit, Conventional CNG | 2011 | 12 | 0.061 | 0.061 | 0.061 | 0.067 | 0.074 | 0.081 | 0.084 | 0.088 | 0.091 | 385 | 10% |  |  |
| Bus School, Conventional Diesel | 2011 | 12 | 0.057 | 0.057 | 0.083 | 0.093 | 0.102 | 0.111 | 0.117 | 0.122 | 0.127 | 818 | 10% |  |  |
| Bus School, Conventional GSL | 2015 | 12 | 0.053 | 0.053 | 0.083 | 0.093 | 0.102 | 0.111 | 0.117 | 0.122 | 0.127 | 538 |  | 15% | 10% |
| Bus School, Conventional EV | 2015 | 12 | 0.148 | 0.148 | 0.178 | 0.193 | 0.210 | 0.230 | 0.237 | 0.244 | 0.253 | 764 |  | 15% | 10% |
| Bust School, Conventional DHEV | 2011 | 12 | 0.062 | 0.062 | 0.114 | 0.125 | 0.138 | 0.151 | 0.157 | 0.164 | 0.170 | 781 | 10% |  |  |
| Bus School, Conventional B20 | 2011 | 12 | 0.057 | 0.057 | 0.083 | 0.093 | 0.103 | 0.112 | 0.118 | 0.124 | 0.131 | 818 | 10% |  |  |
| Bus School, Conventional LPG | 2011 | 12 | 0.048 | 0.048 | 0.083 | 0.093 | 0.102 | 0.111 | 0.117 | 0.122 | 0.127 | 538 |  | 15% | 10% |
| Bus School, Conventional CNG | 2011 | 15.5 | 0.049 | 0.049 | 0.083 | 0.093 | 0.102 | 0.111 | 0.117 | 0.122 | 0.127 | 831 | 10% |  |  |
| Commercial Truck, Conventional Diesel | 2011 | 15.5 | 0.103 | 0.108 | 0.083 | 0.093 | 0.102 | 0.111 | 0.117 | 0.122 | 0.127 | 309 | 10% |  |  |
| Commercial Truck, Conventional Gasoline | 2020 | 15.5 | 0.095 | 0.100 | 0.083 | 0.093 | 0.102 | 0.111 | 0.117 | 0.122 | 0.127 | 309 |  | 15% | 10% |
| Commercial Truck, ELC | 2025 | 15.5 | 0.269 | 0.269 | 0.269 | 0.269 | 0.269 | 0.230 | 0.237 | 0.244 | 0.253 | 188 |  | 15% | 10% |
| Commercial Truck, Diesel HEV | 2015 | 15.5 | 0.118 | 0.118 | 0.114 | 0.125 | 0.138 | 0.151 | 0.157 | 0.164 | 0.170 | 278 | 10% |  |  |
| Commercial Truck, Conventional B20 | 2011 | 15.5 | 0.103 | 0.103 | 0.083 | 0.093 | 0.103 | 0.112 | 0.118 | 0.124 | 0.131 | 309 |  | 15% | 10% |
| Commercial Truck, Conventional LPG | 2011 | 15.5 | 0.086 | 0.086 | 0.083 | 0.093 | 0.102 | 0.111 | 0.117 | 0.122 | 0.127 | 203 |  | 15% | 10% |
| Commercial Truck, Conventional CNG | 2011 | 15.5 | 0.082 | 0.082 | 0.083 | 0.093 | 0.102 | 0.111 | 0.117 | 0.122 | 0.127 | 203 | 10% |  |  |
| Commercial Truck, Conventional E85 | 2011 | 19 | 0.086 | 0.086 | 0.083 | 0.093 | 0.102 | 0.111 | 0.117 | 0.122 | 0.127 | 203 | 10% |  |  |
| Medium Duty Truck, Conventional Diesel | 2011 | 19 | 0.083 | 0.083 | 0.083 | 0.093 | 0.102 | 0.111 | 0.117 | 0.122 | 0.127 | 101 | 10% |  |  |
| Medium Duty Truck, Conventional Gasoline | 2020 | 19 | 0.083 | 0.083 | 0.083 | 0.093 | 0.102 | 0.111 | 0.117 | 0.122 | 0.127 | 153 |  | 15% | 10% |
| Medium Duty Truck, ELC | 2020 | 19 | 0.178 | 0.178 | 0.178 | 0.193 | 0.210 | 0.230 | 0.237 | 0.244 | 0.253 | 104 |  | 15% | 10% |
| Medium Duty Truck, Diesel HEV | 2011 | 19 | 0.083 | 0.083 | 0.083 | 0.093 | 0.103 | 0.112 | 0.118 | 0.124 | 0.131 | 118 | 10% |  |  |
| Medium Duty Truck, Conventional B20 | 2011 | 19 | 0.083 | 0.083 | 0.083 | 0.093 | 0.102 | 0.111 | 0.117 | 0.122 | 0.127 | 153 |  | 15% | 10% |
| Medium Duty Truck, Conventional LPG | 2011 | 19 | 0.083 | 0.083 | 0.083 | 0.093 | 0.102 | 0.111 | 0.117 | 0.122 | 0.127 | 101 |  | 15% | 10% |
| Medium Duty Truck, Conventional CNG | 2011 | 19 | 0.083 | 0.083 | 0.083 | 0.093 | 0.102 | 0.111 | 0.117 | 0.122 | 0.127 | 162 | 10% |  |  |
| Heavy Truck Long Haul, Conv. Diesel | 2011 | 19 | 0.061 | 0.045 | 0.061 | 0.067 | 0.074 | 0.081 | 0.084 | 0.088 | 0.091 | 157 | 10% |  |  |
| Heavy Truck Long Haul, ELC | 2025 | 19 | 0.068 | 0.037 | 0.068 | 0.074 | 0.082 | 0.089 | 0.093 | 0.097 | 0.100 | 132 |  | 15% | 10% |
| Heavy Truck Long Haul, Diesel HEV | 2020 | 19 | 0.065 | 0.065 | 0.107 | 0.118 | 0.132 | 0.145 | 0.151 | 0.157 | 0.163 | 142 |  | 15% | 10% |
| Heavy Truck Long Haul, Conventional B20 | 2011 | 19 | 0.060 | 0.045 | 0.060 | 0.067 | 0.074 | 0.081 | 0.085 | 0.088 | 0.092 | 157 | 10% |  |  |
| Heavy Truck Short Haul, Conv. Diesel | 2011 | 19 | 0.061 | 0.045 | 0.061 | 0.067 | 0.074 | 0.081 | 0.084 | 0.088 | 0.091 | 157 | 10% |  |  |
| Heavy Truck Short Haul, ELC | 2011 | 19 | 0.061 | 0.044 | 0.061 | 0.067 | 0.074 | 0.081 | 0.084 | 0.088 | 0.091 | 132 | 10% |  |  |
| Heavy Truck Short Haul, Diesel HEV | 2025 | 19 | 0.061 | 0.036 | 0.061 | 0.067 | 0.074 | 0.081 | 0.084 | 0.088 | 0.091 | 142 |  | 15% | 10% |
| Heavy Truck Short Haul, Conventional B20 | 2020 | 19 | 0.107 | 0.107 | 0.107 | 0.118 | 0.132 | 0.145 | 0.151 | 0.157 | 0.163 | 157 |  | 15% | 10% |
| Heavy Truck Short Haul, Conventional LPG | 2011 | 19 | 0.060 | 0.049 | 0.060 | 0.067 | 0.074 | 0.081 | 0.085 | 0.088 | 0.092 | 101 | 10% |  |  |
| Heavy Truck Short Haul, Conventional CNG | 2011 | 19 | 0.061 | 0.044 | 0.061 | 0.067 | 0.074 | 0.081 | 0.084 | 0.088 | 0.091 | 162 |  | 15% | 10% |

**Table S10:** End-use load shape – fraction of demand per time slices

| Load Shape | FDAM | FDPM | FN | FP | PDAM | PDPM | PN | PP | SDAM | SDPM | SN | SP | WDAM | WDPM | WN | WP |
| --- | --- | --- | --- | --- | --- | --- | --- | --- | --- | --- | --- | --- | --- | --- | --- | --- |
| TBS | 0.0374 | 0.0303 | 0.0891 | 0.0107 | 0.0374 | 0.0303 | 0.0891 | 0.0107 | 0.0873 | 0.0623 | 0.1425 | 0.0356 | 0.0618 | 0.0601 | 0.1943 | 0.0212 |
| TBT | 0.0409 | 0.0348 | 0.0834 | 0.0083 | 0.0409 | 0.0348 | 0.0834 | 0.0083 | 0.0954 | 0.0737 | 0.1334 | 0.0278 | 0.0676 | 0.0689 | 0.1820 | 0.0165 |
| THS | 0.0774 | 0.0731 | 0.0000 | 0.0129 | 0.0774 | 0.0731 | 0.0000 | 0.0129 | 0.1807 | 0.1506 | 0.0000 | 0.0430 | 0.1280 | 0.1451 | 0.0000 | 0.0256 |
| TM | 0.0119 | 0.0483 | 0.0994 | 0.0085 | 0.0119 | 0.0483 | 0.0994 | 0.0085 | 0.0278 | 0.0994 | 0.1590 | 0.0284 | 0.0197 | 0.0965 | 0.2168 | 0.0161 |
| THL | 0.0928 | 0.0276 | 0.0309 | 0.0133 | 0.0928 | 0.0276 | 0.0309 | 0.0133 | 0.2165 | 0.0586 | 0.0495 | 0.0442 | 0.1534 | 0.0548 | 0.0675 | 0.0263 |
| TC | 0.0119 | 0.0483 | 0.0994 | 0.0085 | 0.0119 | 0.0483 | 0.0994 | 0.0085 | 0.0278 | 0.0994 | 0.1590 | 0.0284 | 0.0197 | 0.0965 | 0.2168 | 0.0161 |

# Constraints

A variety of constraints were used to enhance the behavioral realism of the model. Growth constraints set bounds on technology capacities to prevent the model from suddenly switching the entire fleet from one fuel type to another when it became cheap to do so (“winner-takes-all” behavior). In the light- and heavy-duty sectors, growth constraints were in place that set lower bounds on gasoline or diesel technologies, and these constraints gradually relaxed to allow for fuel switching to occur across the model period. Many of these constraints were dropped for the heavy-duty sector in the mandate scenarios since they directly conflicted with rapid, large-scale electrification of the fleet. Other constraints were in place to reflect modeler’s judgment about technology adoption that may have conflicted with TIMES’ purely cost-based decision-making. For instance, the prices of gasoline and the capital costs of gasoline-fueled heavy-duty short-haul trucks could lead the model to commit most of the heavy-duty short-haul fleet to gasoline when the growth constraints described above were lifted for the mandate scenarios. This conflicted with the reality that most heavy-duty trucks use diesel fuel, not gasoline, due to its higher volumetric energy density, its ability to provide higher torque, and the tendency of diesel-powered trucks to last longer before needing maintenance than gasoline-powered trucks. Therefore, gasoline was constrained to an upper limit of 30% of THS demand. In another instance, a constraint was added to the school bus sub-sector to require 30% of demand to be met by EV school buses in 2030 and 100% by 2050 in the mandate scenarios since runs without this constraint resulted in school buses being the last sector to electrify, with the model sometimes not even buying the first electric buses until just after the modeling period (since the mandate constrains only new capacity, and the model can choose to buy zero new capacity). Since school buses are already being electrified and initiatives are in place to dramatically increase the share of electric buses within the next decade, this constraint ensured that the relative ease of electrifying school buses compared to heavy-duty long-haul trucks was reflected.


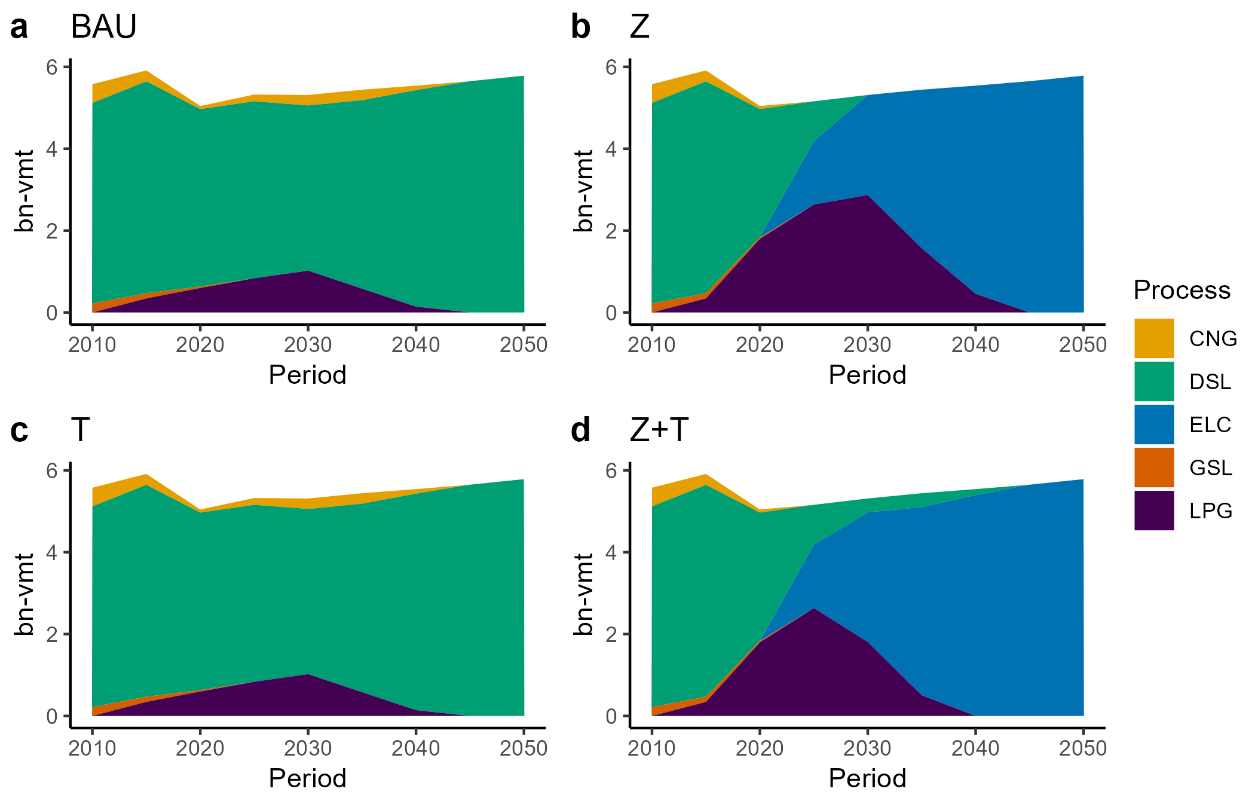


Figure S2. School bus (“TBS”) demand by technology in billion vehicle miles traveled. *CNG: Compressed natural gas, DSL: diesel, ELC: battery electric, GSL: gasoline, LPG: liquified petroleum gas.*


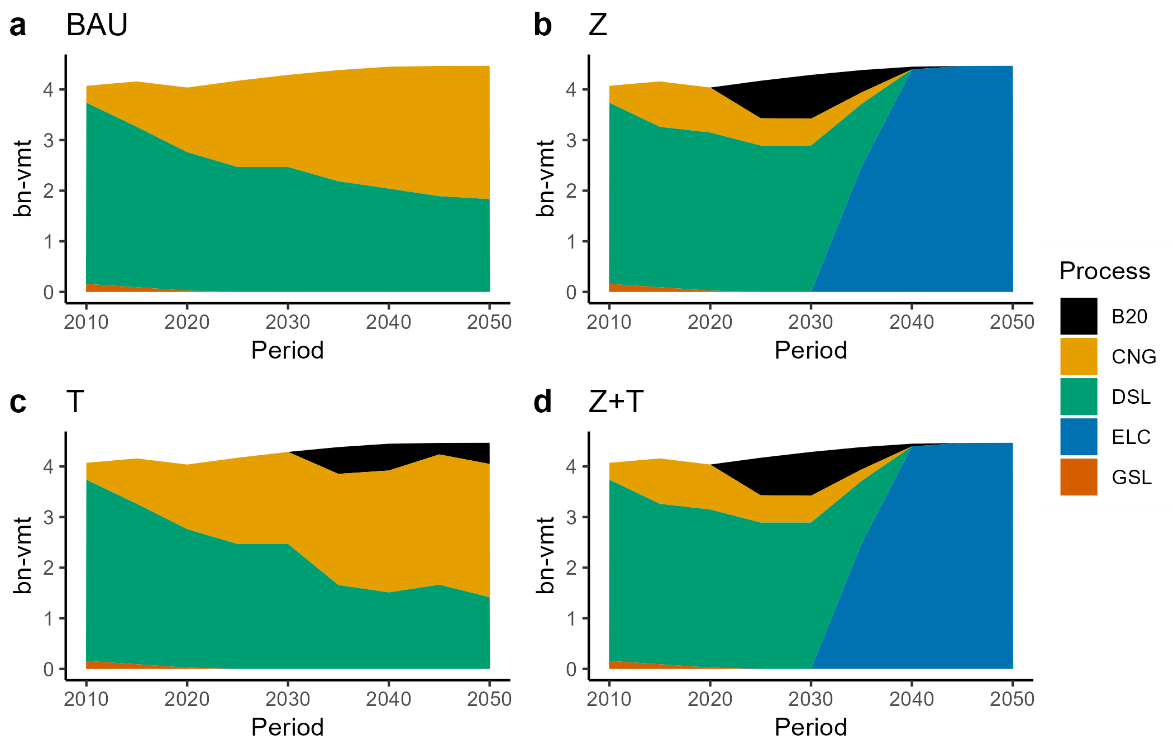


Figure S3. Transit bus (“TBT”) demand by technology in billion vehicle miles traveled. *B20: biodiesel,* *CNG: Compressed natural gas, DSL: diesel, ELC: battery electric, GSL: gasoline.*


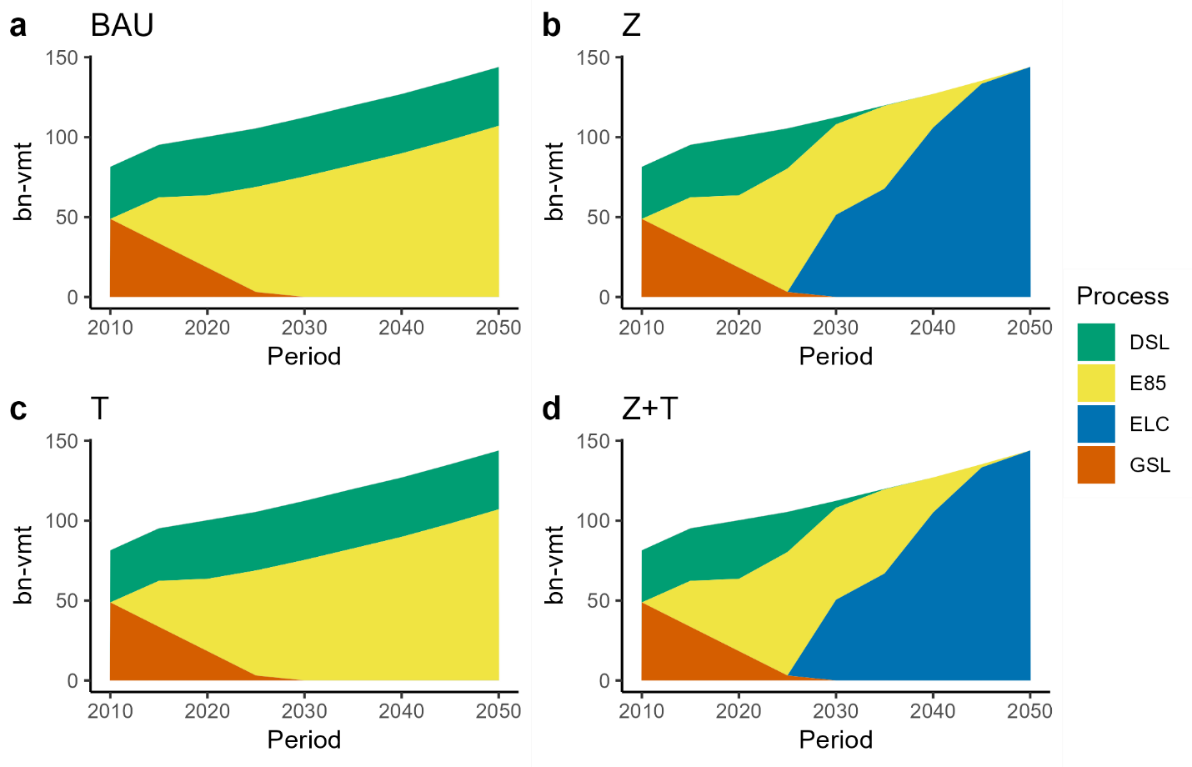


Figure S4. Light commercial truck (“TC”) demand by technology in billion vehicle miles traveled.  *DSL: diesel, E85: ethanol, ELC: battery electric, GSL: gasoline.*


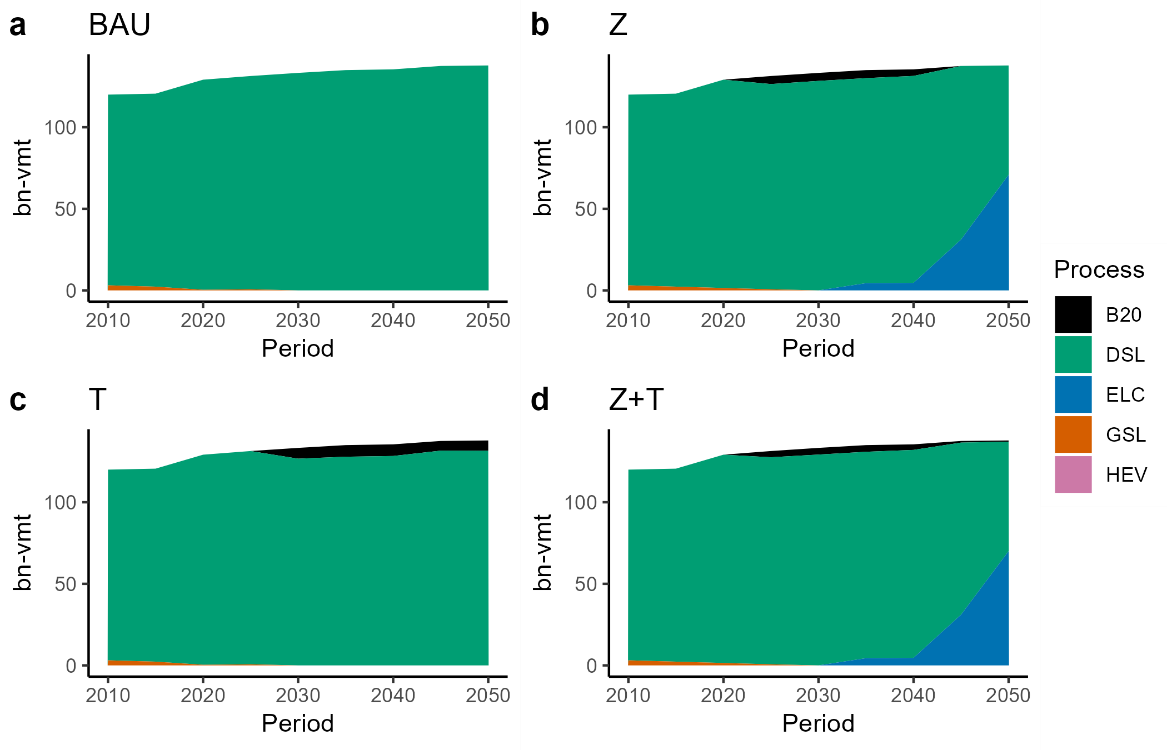


Figure S5. Heavy-duty long-haul truck (“THL”) demand by technology in billion vehicle miles traveled. *B20*: *biodiesel*, *DSL: diesel, ELC: battery electric, GSL: gasoline.*


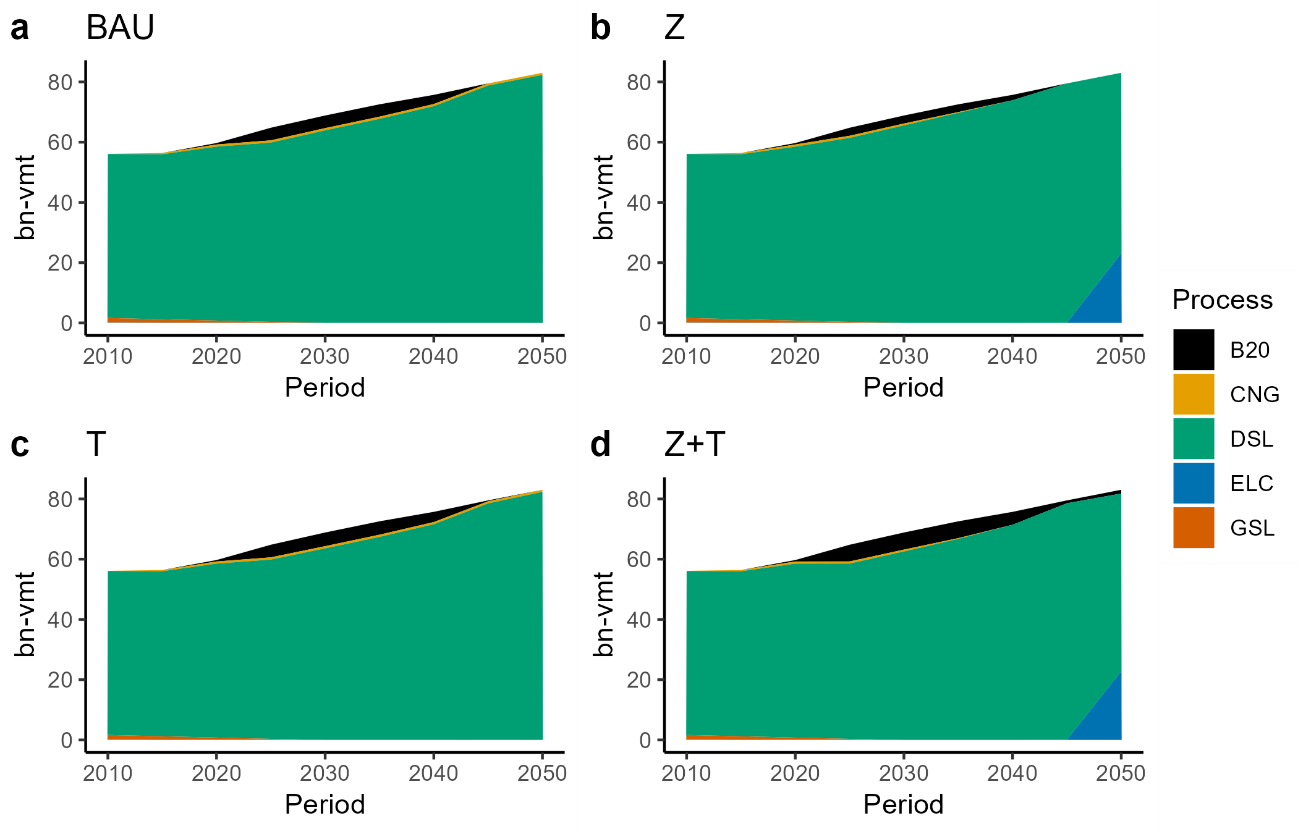


Figure S6. Heavy-duty short-haul truck (“THS”) demand by technology in billion vehicle miles traveled. *CNG: Compressed natural gas, DSL: diesel, ELC: battery electric, GSL: gasoline.*


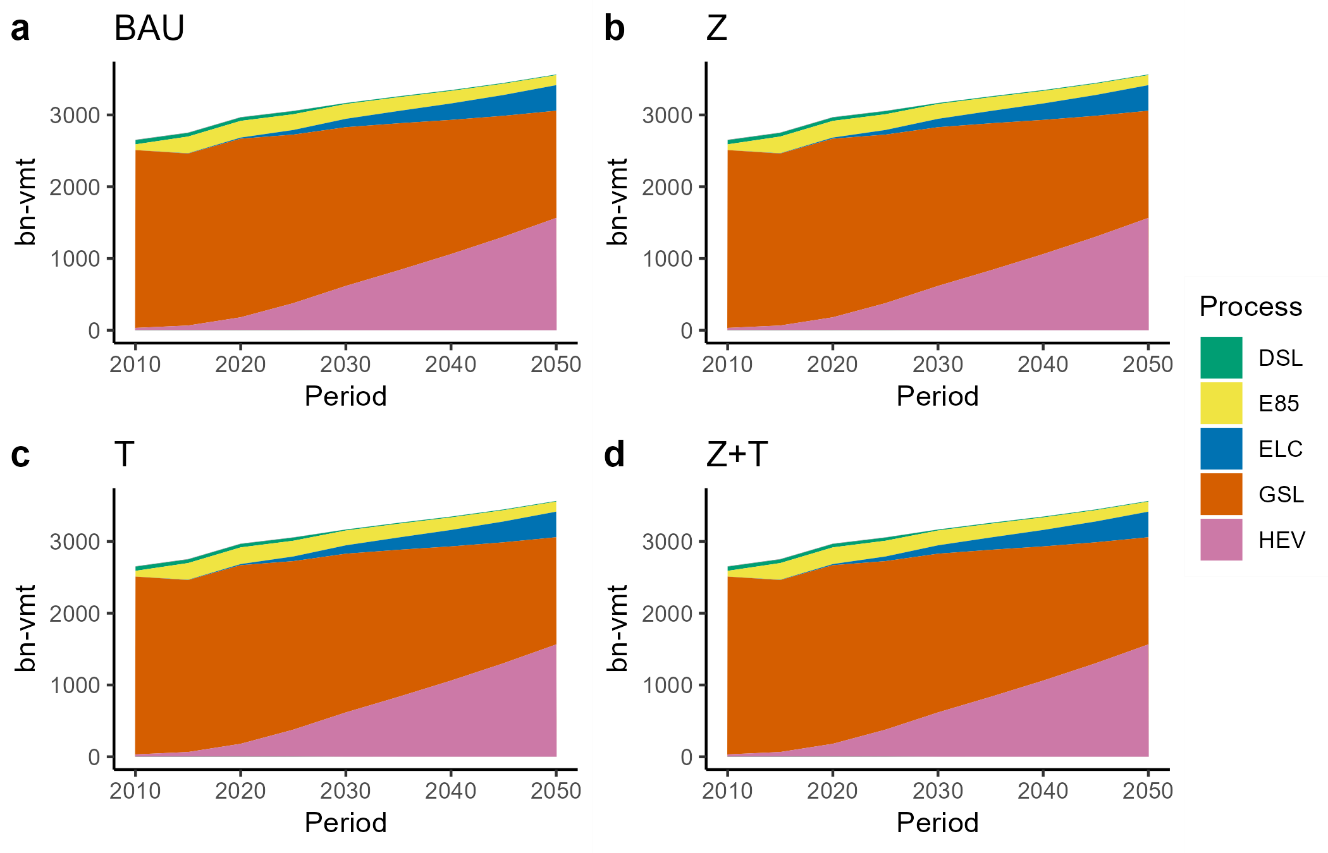


Figure S7. Light-duty (“TL”) demand by technology in billion vehicle miles traveled. *DSL: diesel, E85: ethanol, ELC: battery electric, GSL: gasoline, HEV: hybrid electric.*


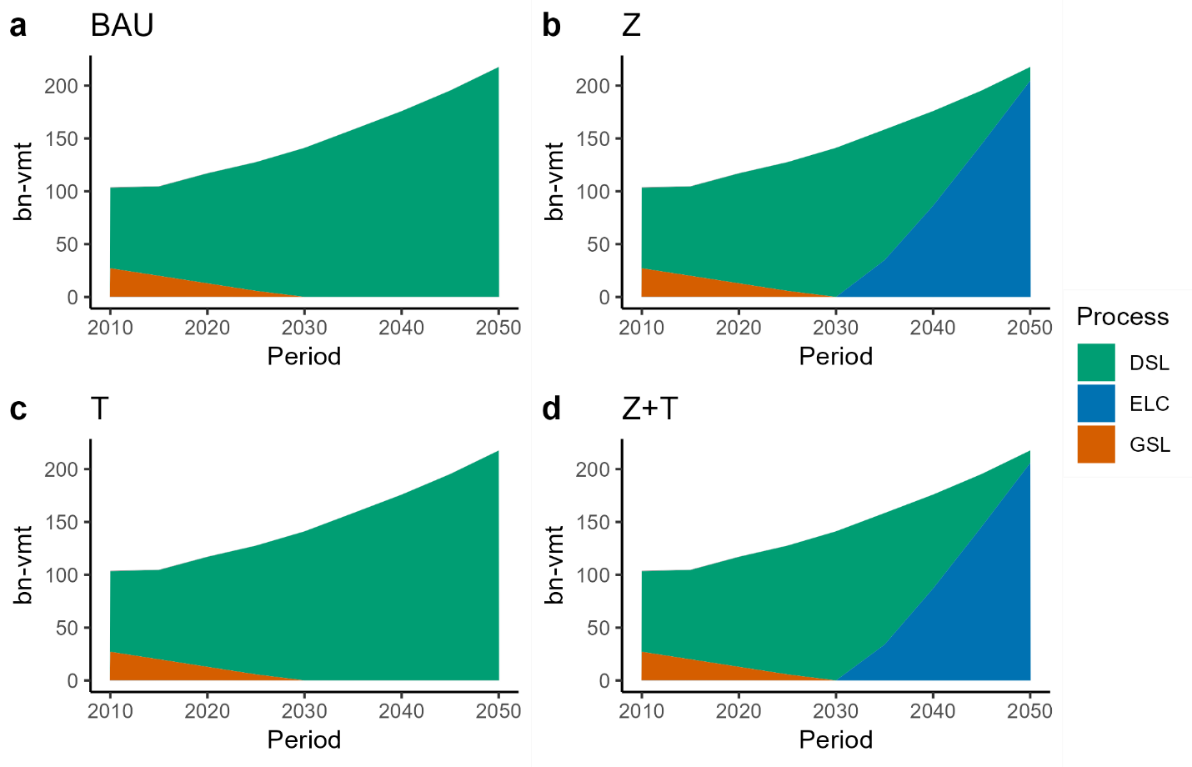


Figure S8. Medium-duty (“TM”) demand by technology in billion vehicle miles traveled. *DSL: diesel, ELC: battery electric, GSL: gasoline.*


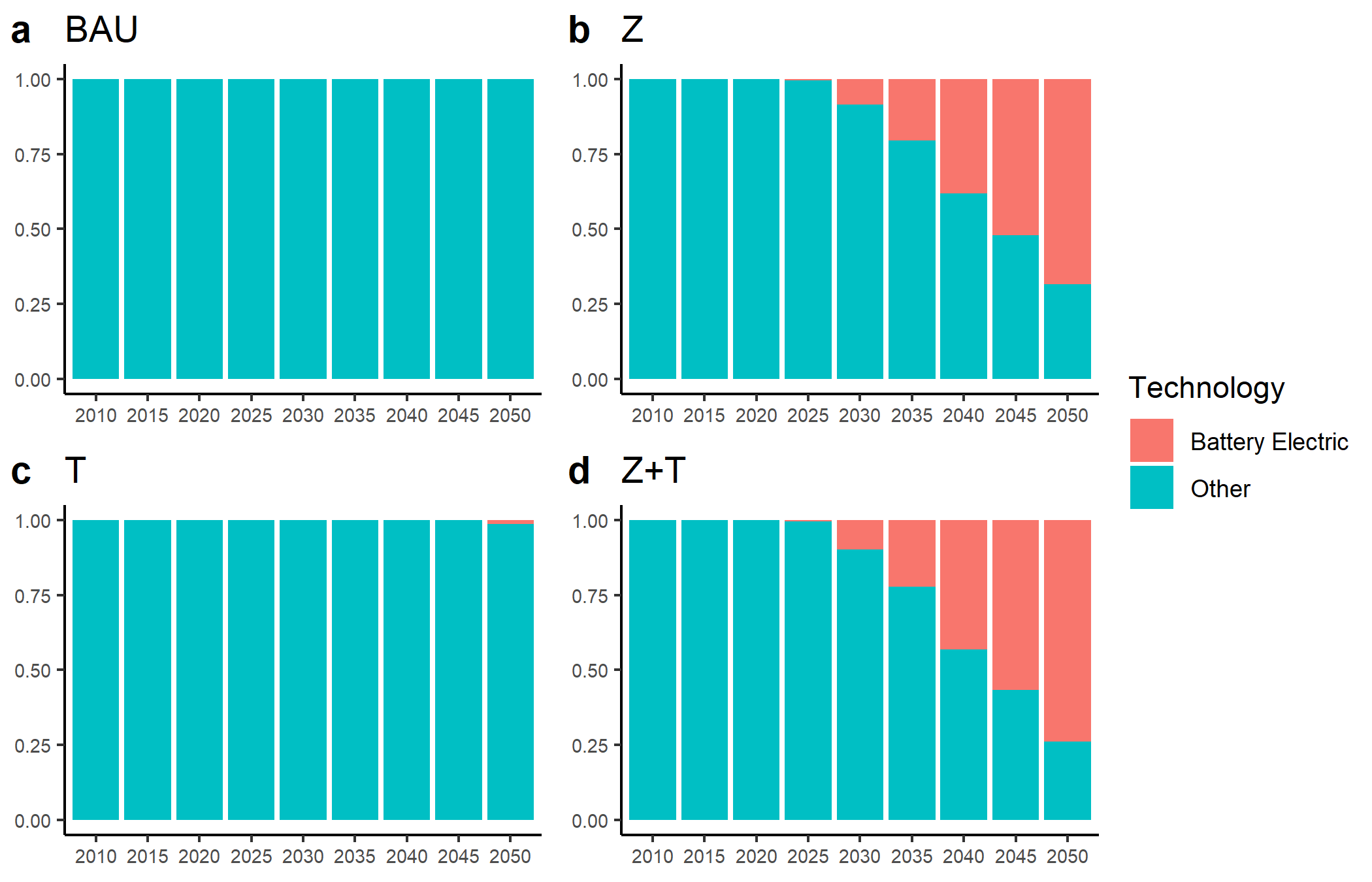


Figure S9. Fraction of MHDV demand met by battery electric vs other fuels

## Note 3: Sensitivity to Cost and Performance Improvements

**Table S11:** Cost assumptions for medium- and heavy-duty vehicles (Advanced scenario from NREL ATB)

| Technology Description | Start Year | Lifetime | Investment Cost, 2005 $US M/demand unit | | | | | | | | |
| --- | --- | --- | --- | --- | --- | --- | --- | --- | --- | --- | --- |
|  |  |  | 2011 | 2015 | 2020 | 2025 | 2030 | 2035 | 2040 | 2045 | 2050 |
| Air Jet, Passenger | 2011 | 30 | 815 | 815 | 809 | 803 | 797 | 791 | 785 |  | 774 |
| Air Jet, Passenger, EV | 2030 | 30 |  |  |  |  | 1214 |  |  |  | 971 |
| Bus Transit, Conventional Diesel | 2011 | 12 | 2752 | 2752 | 2752 | 2786 | 2975 | 3078 | 3113 | 3130 | 3164 |
| Bus Transit, Conventional GSL | 2011 | 12 | 2972 | 2972 | 2972 | 3009 | 3213 | 3325 | 3362 | 3380 | 3418 |
| Bus Transit, Conventional EV | 2015 | 12 | 6140 | 6140 | 6140 | 4196 | 3680 | 3405 | 3302 | 3199 | 3096 |
| Bus Transit, Hydrogen Fuel Cell | 2011 | 12 | 6002 | 6002 | 6002 | 4317 | 3457 | 3182 | 3130 | 3078 | 3027 |
| Bust Transit, Conventional DHEV | 2015 | 12 | 2958 | 2958 | 2958 | 2975 | 3164 | 3250 | 3268 | 3302 | 3319 |
| Bus Transit, Conventional B20 | 2011 | 12 | 2752 | 2752 | 2752 | 2786 | 2975 | 3078 | 3113 | 3130 | 3164 |
| Bus Transit, Conventional CNG | 2011 | 12 | 2752 | 2752 | 2752 | 2786 | 2975 | 3078 | 3113 | 3130 | 3164 |
| Bus School, Conventional Diesel | 2011 | 12 | 9681 | 9681 | 4277 | 4303 | 4458 | 4494 | 4525 | 4561 | 4592 |
| Bus School, Conventional GSL | 2011 | 12 | 10456 | 10456 | 4277 | 4303 | 4458 | 4494 | 4525 | 4561 | 4592 |
| Bus School, Conventional EV | 2015 | 12 | 29564 | 29564 | 5521 | 4545 | 4365 | 4236 | 4195 | 4153 | 4112 |
| Bust School, Conventional DHEV | 2015 | 12 | 16135 | 16135 | 8049 | 6295 | 5128 | 4829 | 4757 | 4690 | 4618 |
| Bus School, Conventional B20 | 2011 | 12 | 9681 | 9681 | 4783 | 4680 | 4793 | 4798 | 4819 | 4840 | 4855 |
| Bus School, Conventional LPG | 2011 | 12 | 9991 | 9991 | 4277 | 4303 | 4458 | 4494 | 4525 | 4561 | 4592 |
| Bus School, Conventional CNG | 2011 | 12 | 12233 | 12233 | 4277 | 4303 | 4458 | 4494 | 4525 | 4561 | 4592 |
| Commercial Truck, Conventional Diesel | 2011 | 15.5 | 1457 | 1457 | 4277 | 4303 | 4458 | 4494 | 4525 | 4561 | 4592 |
| Commercial Truck, Conventional Gasoline | 2011 | 15.5 | 1061 | 1061 | 4277 | 4303 | 4458 | 4494 | 4525 | 4561 | 4592 |
| Commercial Truck, ELC | 2020 | 15.5 | 2099 | 2099 | 5521 | 4545 | 4365 | 4236 | 4195 | 4153 | 4112 |
| Commercial Truck, Diesel HEV | 2025 | 15.5 | 1544 | 1544 | 8049 | 6295 | 5128 | 4829 | 4757 | 4690 | 4618 |
| Commercial Truck, Conventional B20 | 2015 | 15.5 | 1457 | 1457 | 4783 | 4680 | 4793 | 4798 | 4819 | 4840 | 4855 |
| Commercial Truck, Conventional LPG | 2011 | 15.5 | 1355 | 1355 | 4277 | 4303 | 4458 | 4494 | 4525 | 4561 | 4592 |
| Commercial Truck, Conventional CNG | 2011 | 15.5 | 1610 | 1610 | 4277 | 4303 | 4458 | 4494 | 4525 | 4561 | 4592 |
| Commercial Truck, Conventional E85 | 2011 | 15.5 | 1100 | 1100 | 4277 | 4303 | 4458 | 4494 | 4525 | 4561 | 4592 |
| Medium Duty Truck, Conventional Diesel | 2011 | 19 | 3139 | 3139 | 4277 | 4303 | 4458 | 4494 | 4525 | 4561 | 4592 |
| Medium Duty Truck, Conventional Gasoline | 2011 | 19 | 3139 | 3139 | 4619 | 4647 | 4814 | 4853 | 4887 | 4926 | 4959 |
| Medium Duty Truck, ELC | 2020 | 19 | 7414 | 7414 | 5521 | 4545 | 4365 | 4236 | 4195 | 4153 | 4112 |
| Medium Duty Truck, Diesel HEV | 2020 | 19 | 3987 | 3987 | 4783 | 4680 | 4793 | 4798 | 4819 | 4840 | 4855 |
| Medium Duty Truck, Conventional B20 | 2011 | 19 | 3139 | 3139 | 4277 | 4303 | 4458 | 4494 | 4525 | 4561 | 4592 |
| Medium Duty Truck, Conventional LPG | 2011 | 19 | 3855 | 3855 | 4277 | 4303 | 4458 | 4494 | 4525 | 4561 | 4592 |
| Medium Duty Truck, Conventional CNG | 2011 | 19 | 5835 | 5835 | 4277 | 4303 | 4458 | 4494 | 4525 | 4561 | 4592 |
| Heavy Truck Long Haul, Conv. Diesel | 2011 | 19 | 683 | 683 | 1905 | 1929 | 2060 | 2131 | 2155 | 2167 | 2191 |
| Heavy Truck Long Haul, ELC | 2025 | 19 | 3870 | 3870 | 4251 | 2905 | 2548 | 2357 | 2286 | 2215 | 2143 |
| Heavy Truck Long Haul, Hydrogen | 2025 | 19 | 4155 | 4155 | 4155 | 2989 | 2393 | 2203 | 2167 | 2131 | 2096 |
| Heavy Truck Long Haul, Diesel HEV | 2020 | 19 | 2048 | 2048 | 2048 | 2060 | 2191 | 2250 | 2262 | 2286 | 2298 |
| Heavy Truck Long Haul, Conventional B20 | 2011 | 19 | 1905 | 1905 | 1905 | 1929 | 2060 | 2131 | 2155 | 2167 | 2191 |
| Heavy Truck Short Haul, Conv. Diesel | 2011 | 19 | 4953 | 4953 | 4953 | 5015 | 5355 | 5541 | 5603 | 5634 | 5696 |
| Heavy Truck Short Haul, ELC | 2025 | 19 | 11052 | 11052 | 11052 | 7553 | 6625 | 6129 | 5944 | 5758 | 5572 |
| Heavy Truck Short Haul, Hydrogen | 2025 | 19 | 10804 | 10804 | 10804 | 7770 | 6222 | 5727 | 5634 | 5541 | 5448 |
| Heavy Truck Short Haul, Diesel HEV | 2020 | 19 | 5325 | 5325 | 5325 | 5355 | 5696 | 5851 | 5882 | 5944 | 5975 |
| Heavy Truck Short Haul, Conventional B20 | 2011 | 19 | 4953 | 4953 | 4953 | 5015 | 5355 | 5541 | 5603 | 5634 | 5696 |
| Heavy Truck Short Haul, Conventional LPG | 2011 | 19 | 4953 | 4953 | 4953 | 5015 | 5355 | 5541 | 5603 | 5634 | 5696 |
| Heavy Truck Short Haul, Conventional CNG | 2011 | 19 | 4953 | 4953 | 4953 | 5015 | 5355 | 5541 | 5603 | 5634 | 5696 |

**Table S12:** Performance assumptions for medium- and heavy-duty vehicles (Advanced scenario from NREL ATB)

| Technology Description | Start Year |  | Efficiency, demand unit/PJ | | | | | | | | | O&M Cost, 2005 $US M/demand unit | Technology-specific discount rate | | |
| --- | --- | --- | --- | --- | --- | --- | --- | --- | --- | --- | --- | --- | --- | --- | --- |
|  |  |  | 2011 | 2015 | 2020 | 2025 | 2030 | 2035 | 2040 | 2045 | 2050 |  | All years | 2025 | 2035 |
| Air Jet, Passenger | 2011 | 30 | 0.451 |  |  |  | 0.519 |  |  |  | 0.587 | 7.46 | 18% |  |  |
| Air Jet, Passenger, EV | 2030 | 30 |  |  |  |  | 1.038 |  |  |  | 1.101 | 9.65 | 24% |  |  |
| Bus Transit, Conventional Diesel | 2011 | 12 | 0.061 | 0.061 | 0.061 | 0.079 | 0.091 | 0.102 | 0.106 | 0.110 | 0.113 | 382 | 10% |  |  |
| Bus Transit, Conventional GSL | 2011 | 12 | 0.068 | 0.068 | 0.068 | 0.087 | 0.100 | 0.113 | 0.117 | 0.121 | 0.125 | 382 | 10% |  |  |
| Bus Transit, Conventional EV | 2015 | 12 | 0.107 | 0.107 | 0.107 | 0.142 | 0.163 | 0.184 | 0.192 | 0.200 | 0.209 | 246 |  | 15% | 10% |
| Bus Transit, Hydrogen Fuel Cell | 2015 | 12 | 0.057 | 0.057 | 0.057 | 0.080 | 0.096 | 0.109 | 0.116 | 0.122 | 0.129 | 246 |  | 15% | 10% |
| Bust Transit, Conventional DHEV | 2011 | 12 | 0.048 | 0.048 | 0.048 | 0.064 | 0.073 | 0.084 | 0.088 | 0.091 | 0.095 | 382 | 10% |  |  |
| Bus Transit, Conventional B20 | 2011 | 12 | 0.061 | 0.061 | 0.061 | 0.079 | 0.091 | 0.102 | 0.106 | 0.110 | 0.113 | 382 |  | 15% | 10% |
| Bus Transit, Conventional CNG | 2011 | 12 | 0.061 | 0.061 | 0.061 | 0.079 | 0.091 | 0.102 | 0.106 | 0.110 | 0.113 | 382 | 10% |  |  |
| Bus School, Conventional Diesel | 2011 | 12 | 0.057 | 0.057 | 0.083 | 0.107 | 0.119 | 0.127 | 0.132 | 0.138 | 0.143 | 817 | 10% |  |  |
| Bus School, Conventional GSL | 2015 | 12 | 0.053 | 0.053 | 0.083 | 0.107 | 0.119 | 0.127 | 0.132 | 0.138 | 0.143 | 537 |  | 15% | 10% |
| Bus School, Conventional EV | 2015 | 12 | 0.148 | 0.148 | 0.178 | 0.230 | 0.253 | 0.275 | 0.282 | 0.288 | 0.295 | 763 |  | 15% | 10% |
| Bust School, Conventional DHEV | 2011 | 12 | 0.062 | 0.062 | 0.114 | 0.149 | 0.168 | 0.184 | 0.189 | 0.195 | 0.200 | 780 | 10% |  |  |
| Bus School, Conventional B20 | 2011 | 12 | 0.057 | 0.057 | 0.083 | 0.109 | 0.122 | 0.132 | 0.137 | 0.143 | 0.149 | 817 | 10% |  |  |
| Bus School, Conventional LPG | 2011 | 12 | 0.048 | 0.048 | 0.083 | 0.107 | 0.119 | 0.127 | 0.132 | 0.138 | 0.143 | 537 |  | 15% | 10% |
| Bus School, Conventional CNG | 2011 | 15.5 | 0.049 | 0.049 | 0.083 | 0.107 | 0.119 | 0.127 | 0.132 | 0.138 | 0.143 | 830 | 10% |  |  |
| Commercial Truck, Conventional Diesel | 2011 | 15.5 | 0.103 | 0.108 | 0.083 | 0.107 | 0.119 | 0.127 | 0.132 | 0.138 | 0.143 | 309 | 10% |  |  |
| Commercial Truck, Conventional Gasoline | 2020 | 15.5 | 0.095 | 0.100 | 0.083 | 0.107 | 0.119 | 0.127 | 0.132 | 0.138 | 0.143 | 309 |  | 15% | 10% |
| Commercial Truck, ELC | 2025 | 15.5 | 0.269 | 0.269 | 0.269 | 0.269 | 0.269 | 0.275 | 0.282 | 0.288 | 0.295 | 188 |  | 15% | 10% |
| Commercial Truck, Diesel HEV | 2015 | 15.5 | 0.118 | 0.118 | 0.114 | 0.149 | 0.168 | 0.184 | 0.189 | 0.195 | 0.200 | 278 | 10% |  |  |
| Commercial Truck, Conventional B20 | 2011 | 15.5 | 0.103 | 0.103 | 0.083 | 0.109 | 0.122 | 0.132 | 0.137 | 0.143 | 0.149 | 309 |  | 15% | 10% |
| Commercial Truck, Conventional LPG | 2011 | 15.5 | 0.086 | 0.086 | 0.083 | 0.107 | 0.119 | 0.127 | 0.132 | 0.138 | 0.143 | 203 |  | 15% | 10% |
| Commercial Truck, Conventional CNG | 2011 | 15.5 | 0.082 | 0.082 | 0.083 | 0.107 | 0.119 | 0.127 | 0.132 | 0.138 | 0.143 | 203 | 10% |  |  |
| Commercial Truck, Conventional E85 | 2011 | 19 | 0.086 | 0.086 | 0.083 | 0.107 | 0.119 | 0.127 | 0.132 | 0.138 | 0.143 | 203 | 10% |  |  |
| Medium Duty Truck, Conventional Diesel | 2011 | 19 | 0.083 | 0.083 | 0.083 | 0.107 | 0.119 | 0.127 | 0.132 | 0.138 | 0.143 | 100 | 10% |  |  |
| Medium Duty Truck, Conventional Gasoline | 2020 | 19 | 0.083 | 0.083 | 0.083 | 0.107 | 0.119 | 0.127 | 0.132 | 0.138 | 0.143 | 152 |  | 15% | 10% |
| Medium Duty Truck, ELC | 2020 | 19 | 0.178 | 0.178 | 0.178 | 0.230 | 0.253 | 0.275 | 0.282 | 0.288 | 0.295 | 103 |  | 15% | 10% |
| Medium Duty Truck, Diesel HEV | 2011 | 19 | 0.083 | 0.083 | 0.083 | 0.109 | 0.122 | 0.132 | 0.137 | 0.143 | 0.149 | 117 | 10% |  |  |
| Medium Duty Truck, Conventional B20 | 2011 | 19 | 0.083 | 0.083 | 0.083 | 0.107 | 0.119 | 0.127 | 0.132 | 0.138 | 0.143 | 152 |  | 15% | 10% |
| Medium Duty Truck, Conventional LPG | 2011 | 19 | 0.083 | 0.083 | 0.083 | 0.107 | 0.119 | 0.127 | 0.132 | 0.138 | 0.143 | 100 |  | 15% | 10% |
| Medium Duty Truck, Conventional CNG | 2011 | 19 | 0.083 | 0.083 | 0.083 | 0.107 | 0.119 | 0.127 | 0.132 | 0.138 | 0.143 | 161 | 10% |  |  |
| Heavy Truck Long Haul, Conv. Diesel | 2011 | 19 | 0.061 | 0.045 | 0.061 | 0.079 | 0.091 | 0.102 | 0.106 | 0.110 | 0.113 | 156 | 10% |  |  |
| Heavy Truck Long Haul, ELC | 2025 | 19 | 0.065 | 0.065 | 0.107 | 0.142 | 0.163 | 0.184 | 0.192 | 0.200 | 0.209 | 132 |  | 15% | 10% |
| Heavy Truck Long Haul, Hydrogen | 2020 | 19 | 0.058 | 0.058 | 0.052 | 0.072 | 0.087 | 0.099 | 0.105 | 0.111 | 0.117 | 0 |  | 15% | 10% |
| Heavy Truck Long Haul, Diesel HEV | 2011 | 19 | 0.060 | 0.045 | 0.060 | 0.079 | 0.092 | 0.105 | 0.110 | 0.114 | 0.119 | 142 | 10% |  |  |
| Heavy Truck Long Haul, Conventional B20 | 2011 | 19 | 0.061 | 0.045 | 0.061 | 0.079 | 0.091 | 0.102 | 0.106 | 0.110 | 0.113 | 156 | 10% |  |  |
| Heavy Truck Short Haul, Conv. Diesel | 2011 | 19 | 0.061 | 0.044 | 0.061 | 0.079 | 0.091 | 0.102 | 0.106 | 0.110 | 0.113 | 156 | 10% |  |  |
| Heavy Truck Short Haul, ELC | 2025 | 19 | 0.107 | 0.107 | 0.107 | 0.142 | 0.163 | 0.184 | 0.192 | 0.200 | 0.209 | 132 |  | 15% | 10% |
| Heavy Truck Short Haul, Hydrogen | 2020 | 19 | 0.058 | 0.058 | 0.065 | 0.090 | 0.109 | 0.124 | 0.131 | 0.138 | 0.146 | 0 |  | 15% | 10% |
| Heavy Truck Short Haul, Diesel HEV | 2011 | 19 | 0.060 | 0.049 | 0.060 | 0.079 | 0.092 | 0.105 | 0.110 | 0.114 | 0.119 | 142 | 10% |  |  |
| Heavy Truck Short Haul, Conventional B20 | 2011 | 19 | 0.061 | 0.044 | 0.061 | 0.079 | 0.091 | 0.102 | 0.106 | 0.110 | 0.113 | 156 |  | 15% | 10% |
| Heavy Truck Short Haul, Conventional LPG | 2011 | 19 | 0.061 | 0.036 | 0.061 | 0.079 | 0.091 | 0.102 | 0.106 | 0.110 | 0.113 | 100 |  | 15% | 10% |
| Heavy Truck Short Haul, Conventional CNG | 2011 | 19 | 0.038 | 0.038 | 0.061 | 0.079 | 0.091 | 0.102 | 0.106 | 0.110 | 0.113 | 162 |  | 15% | 10% |


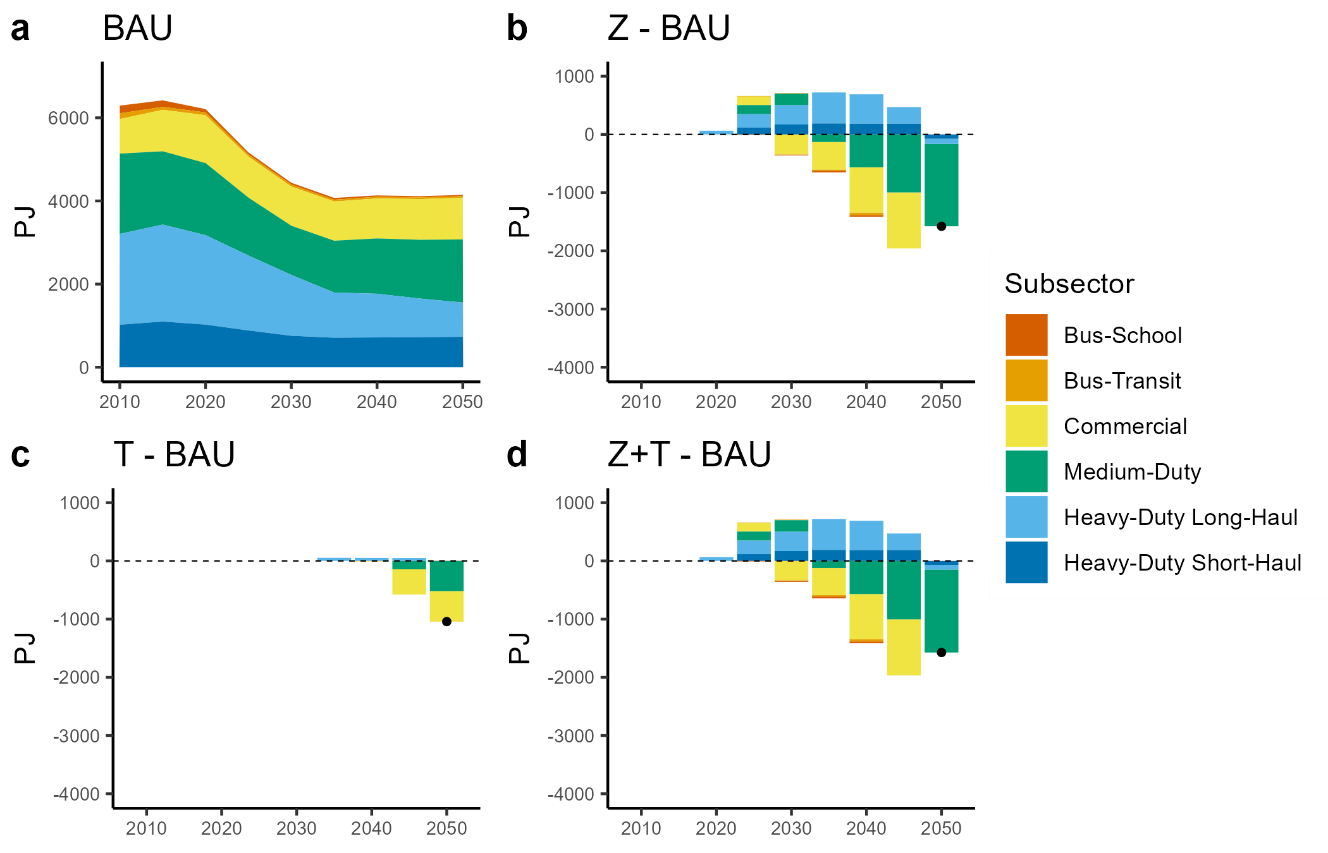


Figure S10: Fossil fuel consumption of medium- and heavy-duty vehicles by transport mode (NREL ATB Adv-Vehicle Scenario)


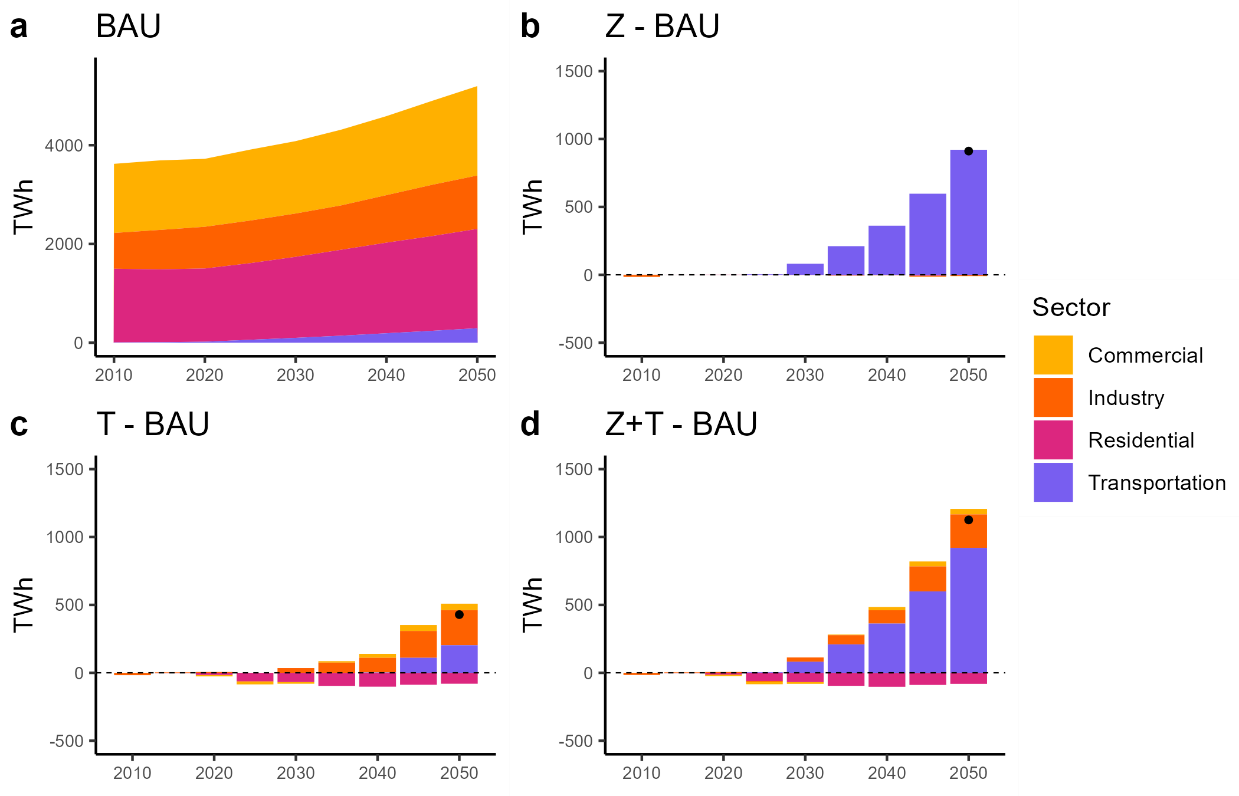


Figure S11: Electricity consumption by end-use (NREL ATB Adv-Vehicle Scenario)


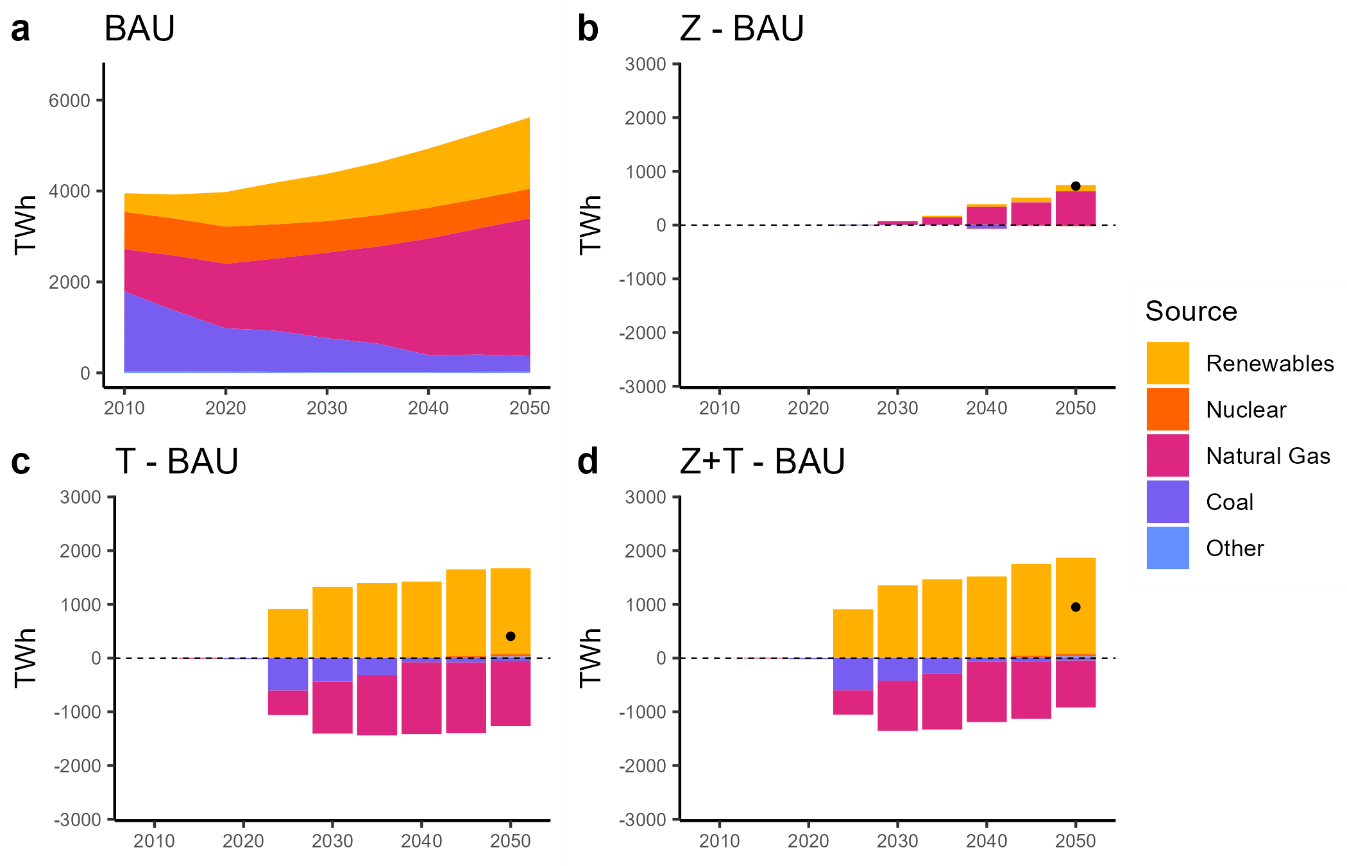


Figure S12: Electricity generation by source. (NREL ATB Adv-Vehicle Scenario)


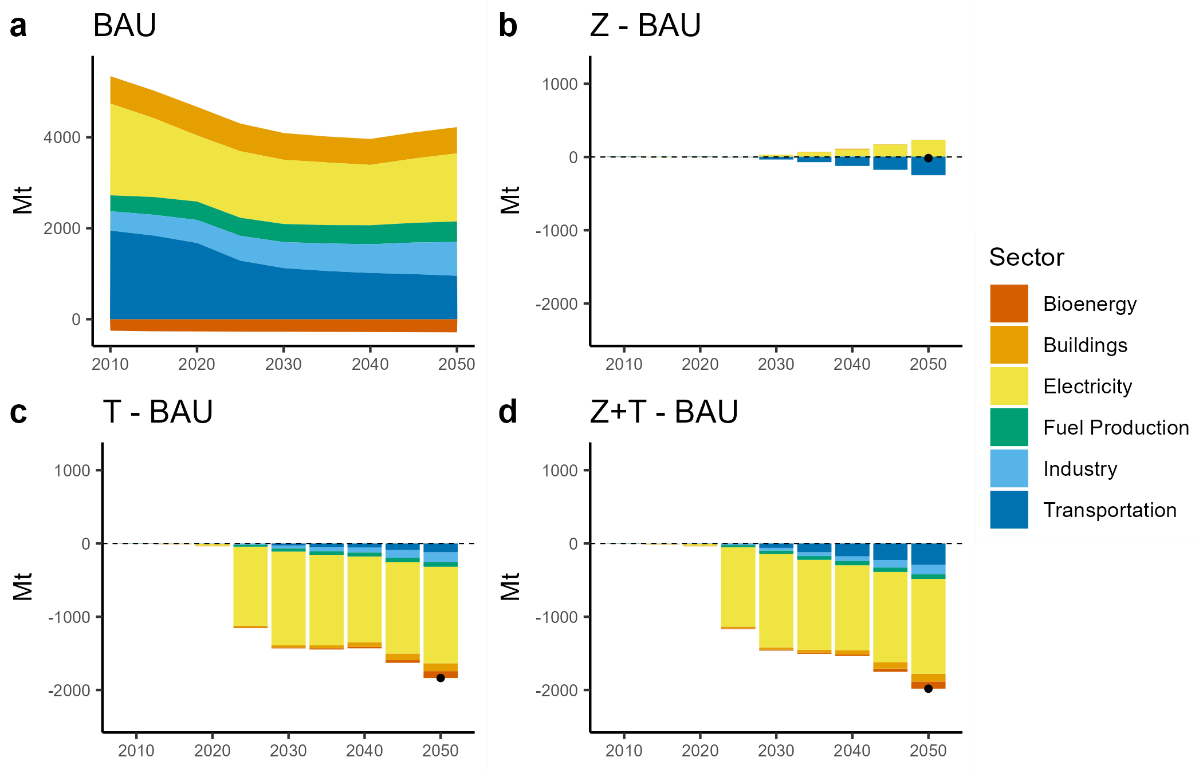


Figure S13: CO_2_ emissions by sector (NREL ATB Adv-Vehicle Scenario)


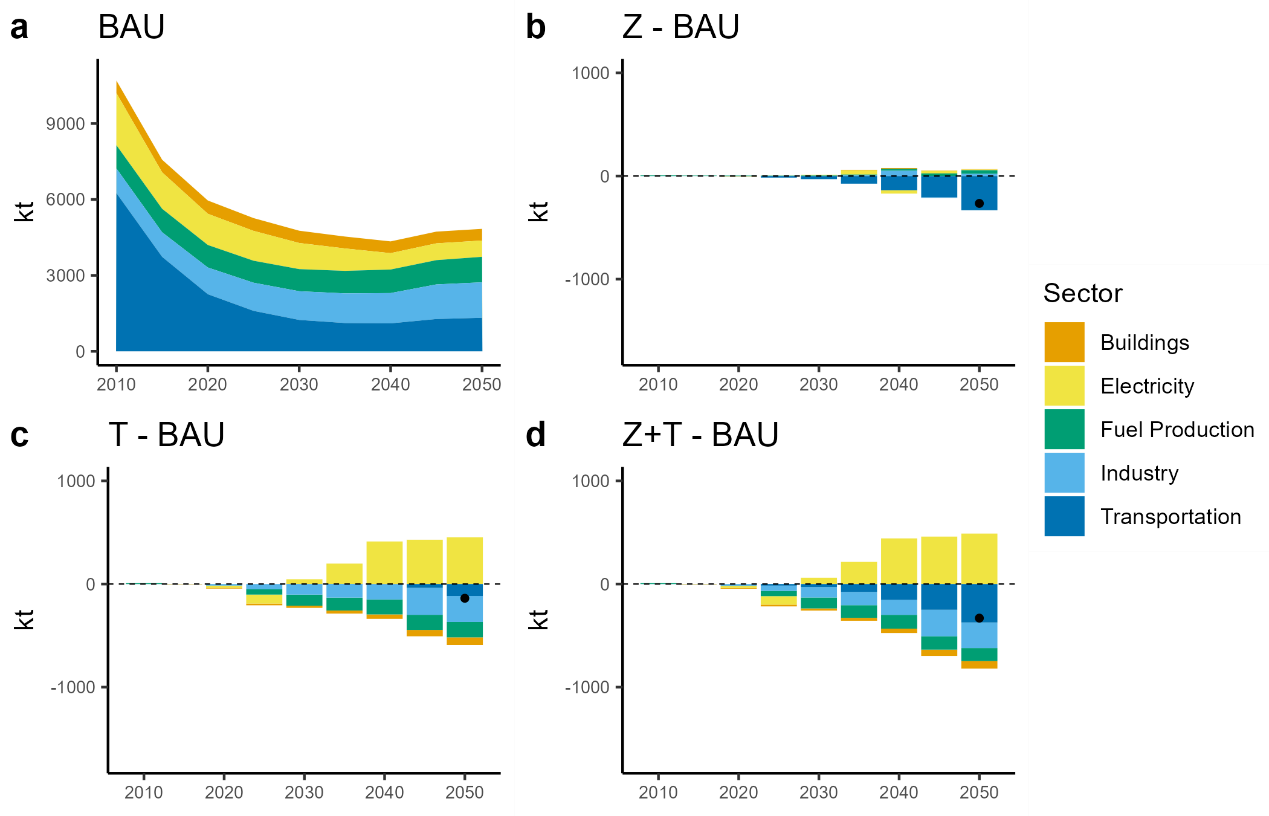


Figure S14: NO_X_ emissions by sector (NREL ATB Adv-Vehicle Scenario)


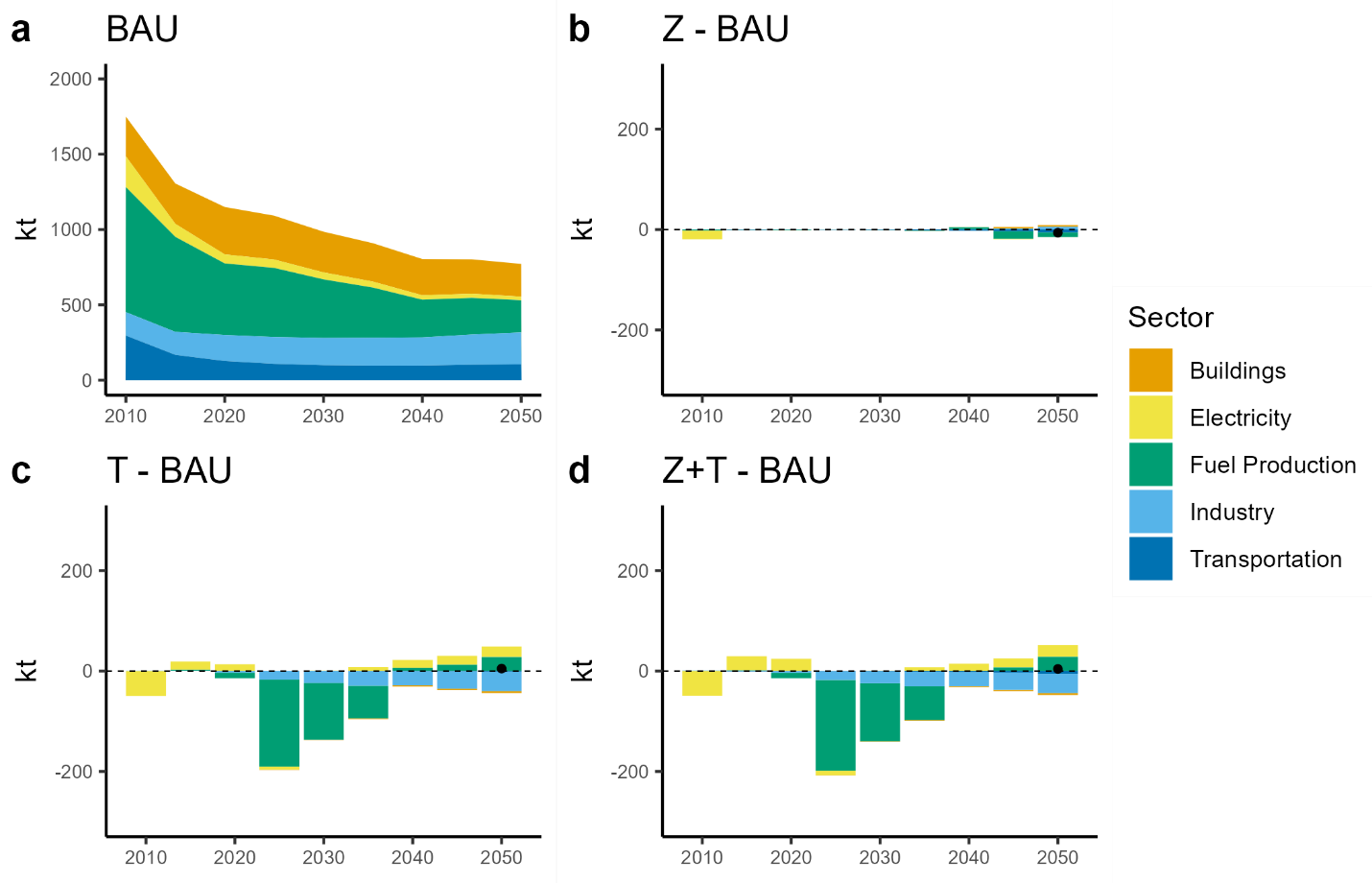


Figure S15: PM_2.5_ emissions by sector (NREL ATB Adv-Vehicle Scenario)


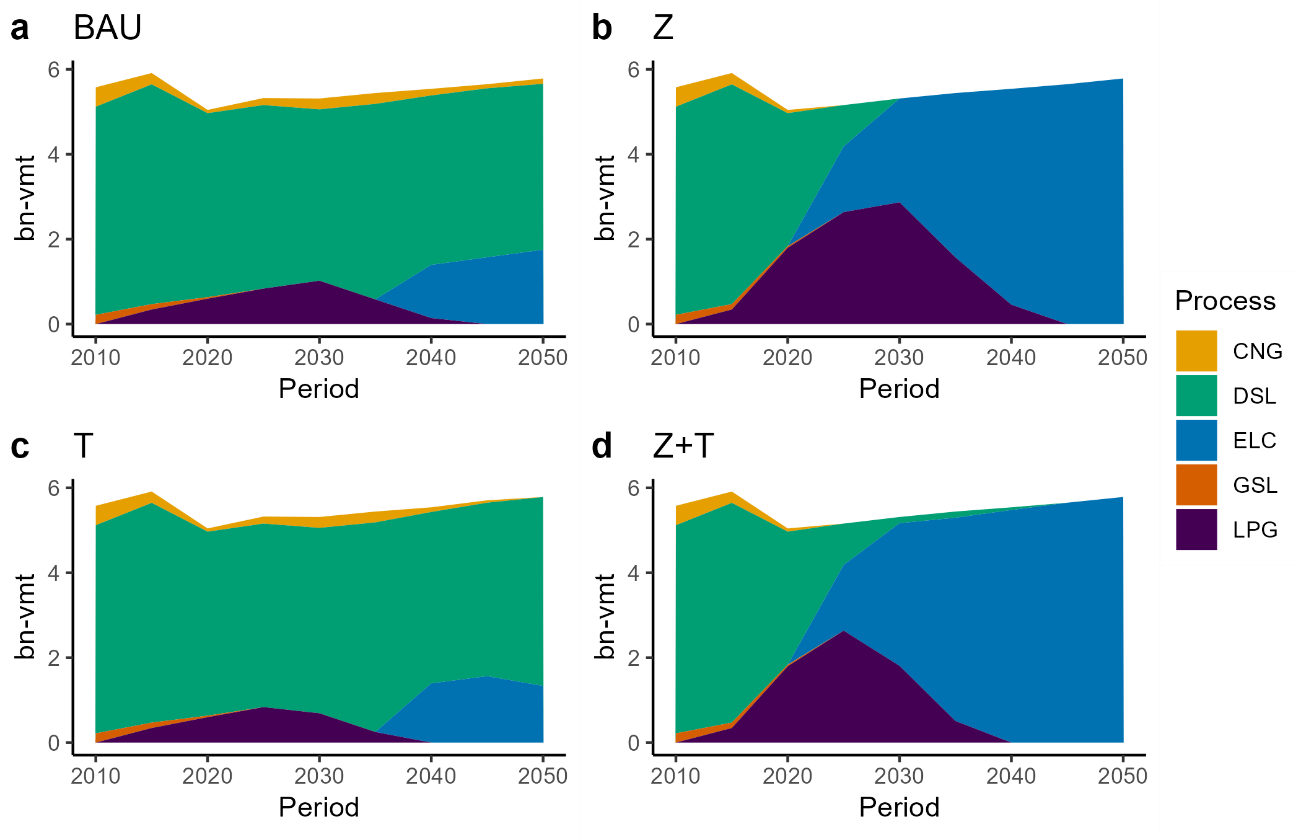


Figure S16. School bus (“TBS”) demand by technology in billion vehicle miles traveled. *CNG: Compressed natural gas, DSL: diesel, ELC: battery electric, GSL: gasoline, LPG: liquified petroleum gas.* (NREL ATB Adv-Vehicle Scenario)


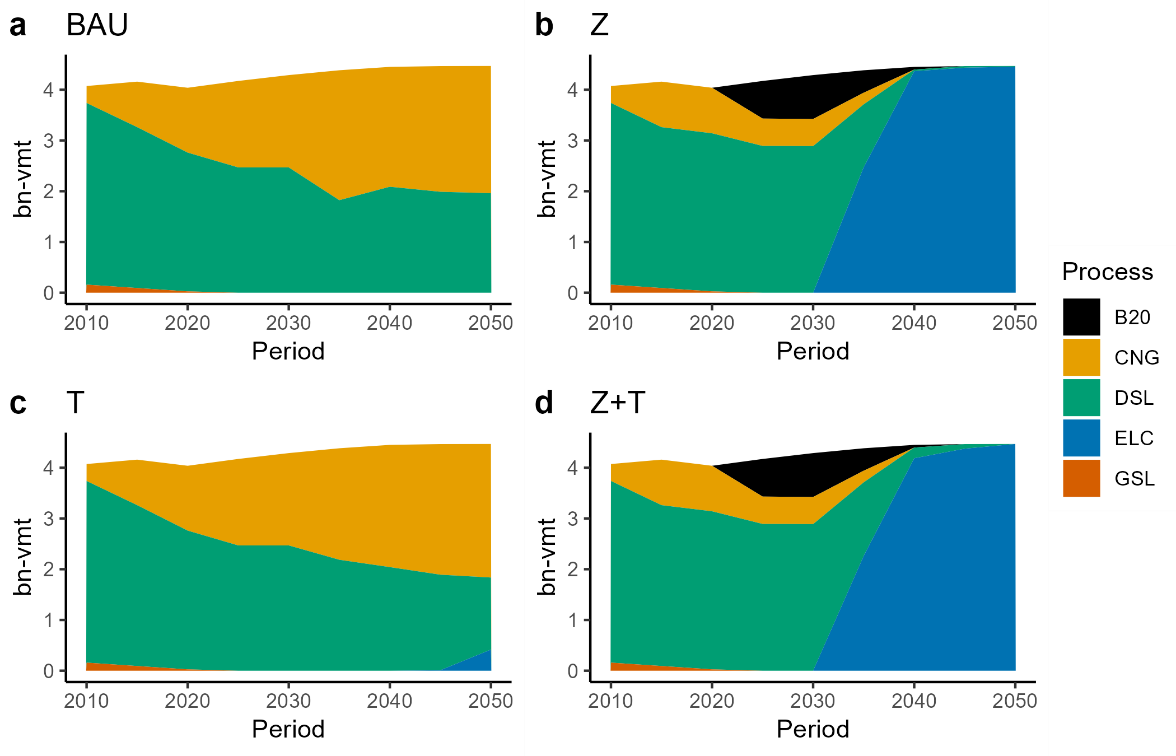


Figure S17. Transit bus (“TBT”) demand by technology in billion vehicle miles traveled. *B20: biodiesel,* *CNG: Compressed natural gas, DSL: diesel, ELC: battery electric, GSL: gasoline.* (NREL ATB Adv-Vehicle Scenario)


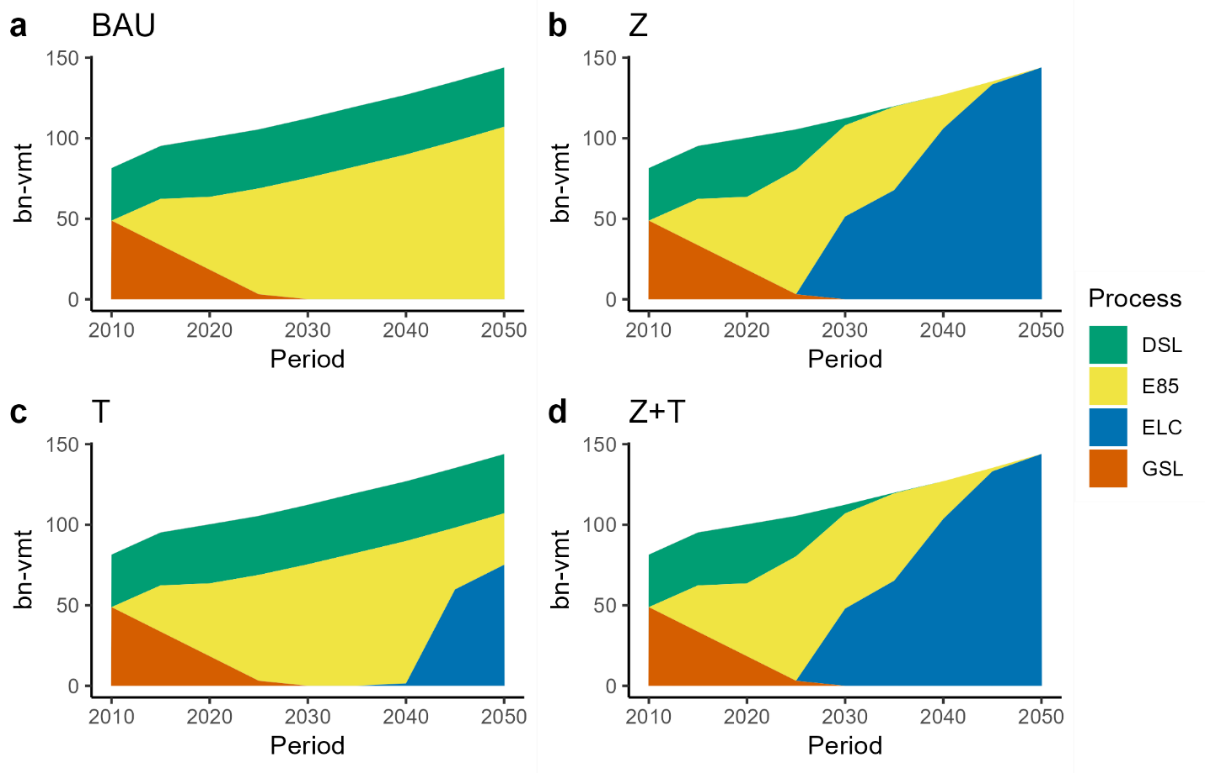


Figure S18. Light commercial truck (“TC”) demand by technology in billion vehicle miles traveled.  *DSL: diesel, E85: ethanol, ELC: battery electric, GSL: gasoline.* (NREL ATB Adv-Vehicle Scenario)


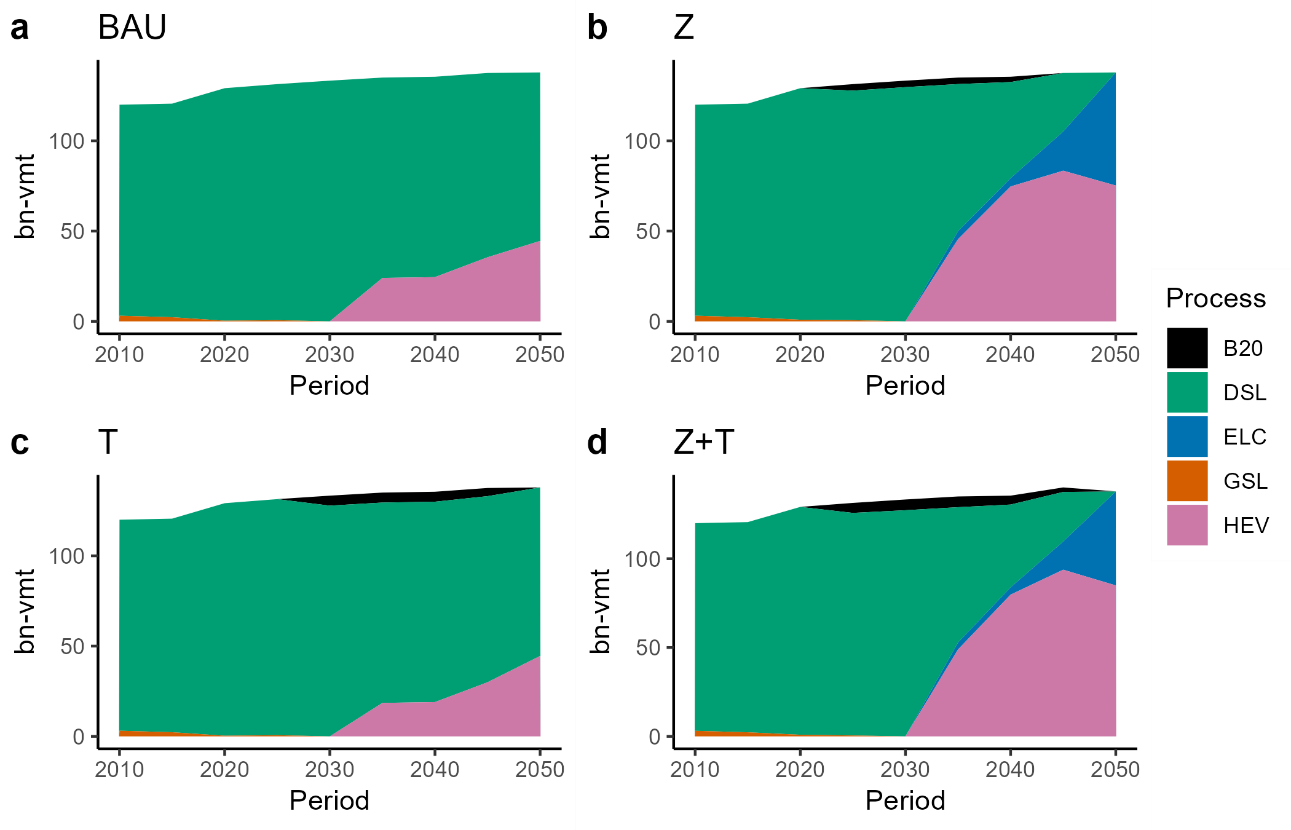


Figure S19. Heavy-duty long-haul truck (“THL”) demand by technology in billion vehicle miles traveled. *B20*: *biodiesel*, *DSL: diesel, ELC: battery electric, GSL: gasoline.* (NREL ATB Adv-Vehicle Scenario)


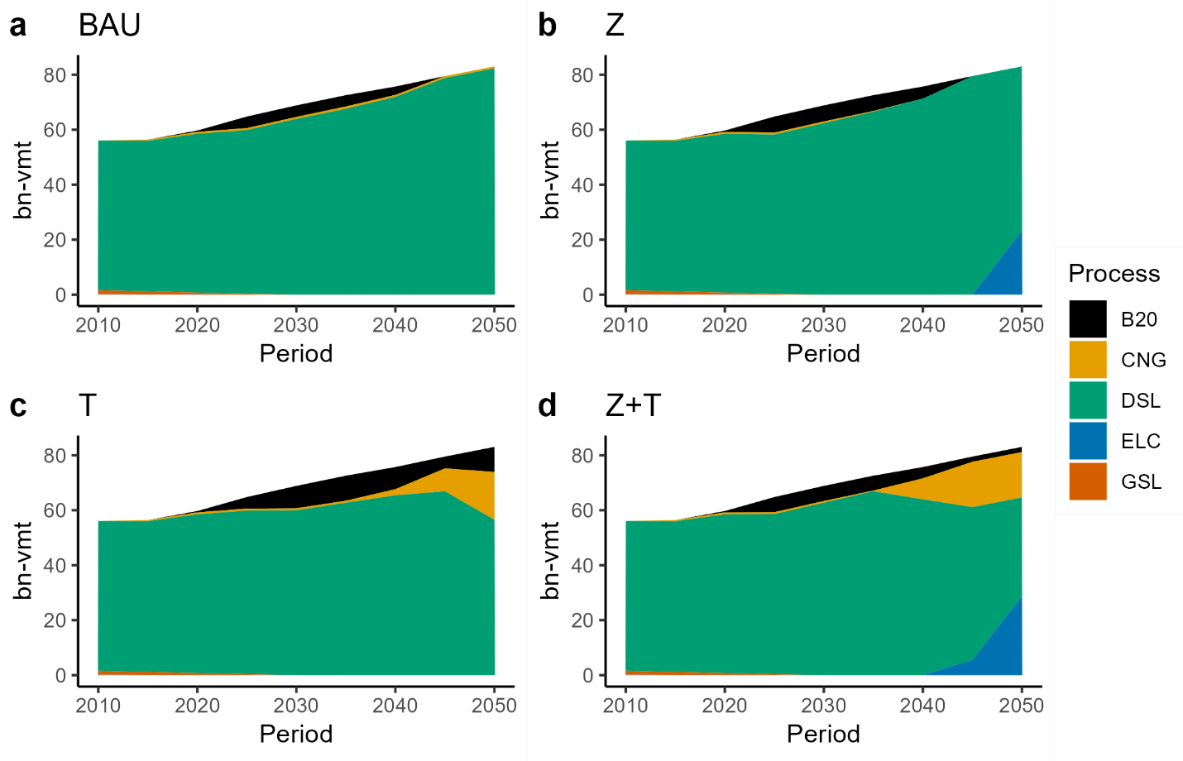


Figure S20. Heavy-duty short-haul truck (“THS”) demand by technology in billion vehicle miles traveled. *CNG: Compressed natural gas, DSL: diesel, ELC: battery electric, GSL: gasoline.* (NREL ATB Adv-Vehicle Scenario)


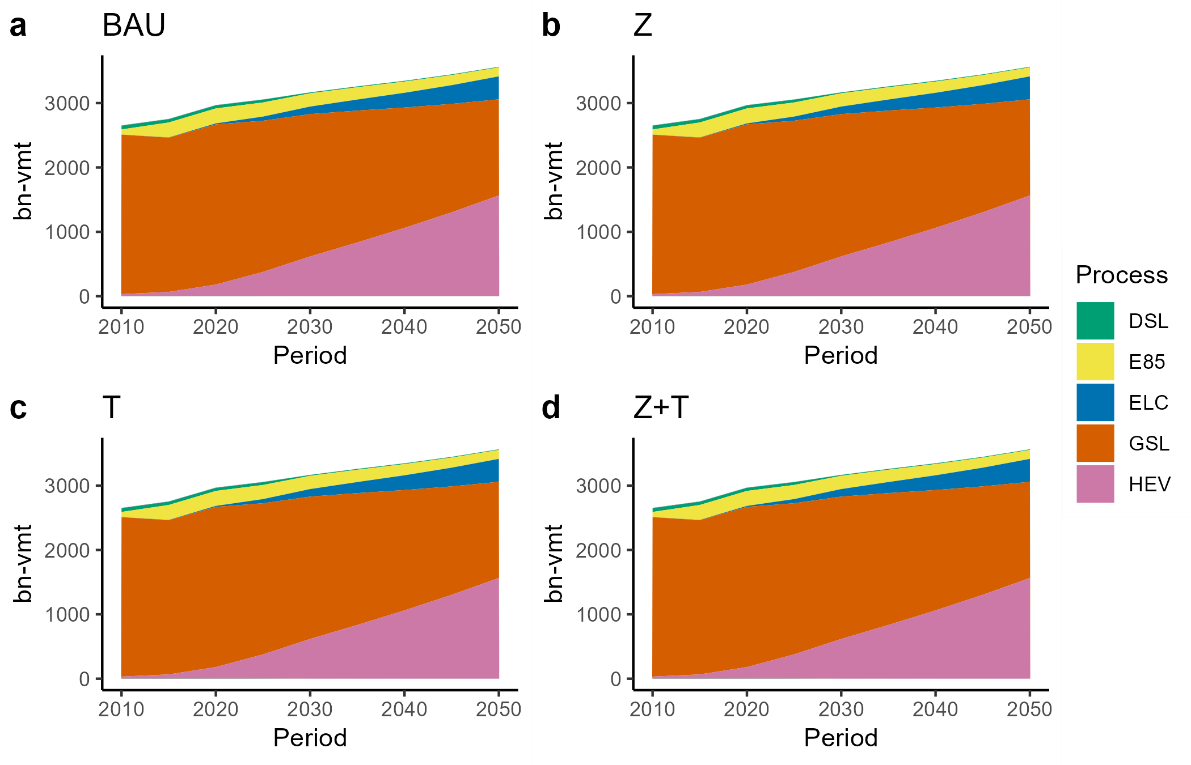


Figure S21. Light-duty (“TL”) demand by technology in billion vehicle miles traveled. *DSL: diesel, E85: ethanol, ELC: battery electric, GSL: gasoline, HEV: hybrid electric.* (NREL ATB Adv-Vehicle Scenario)


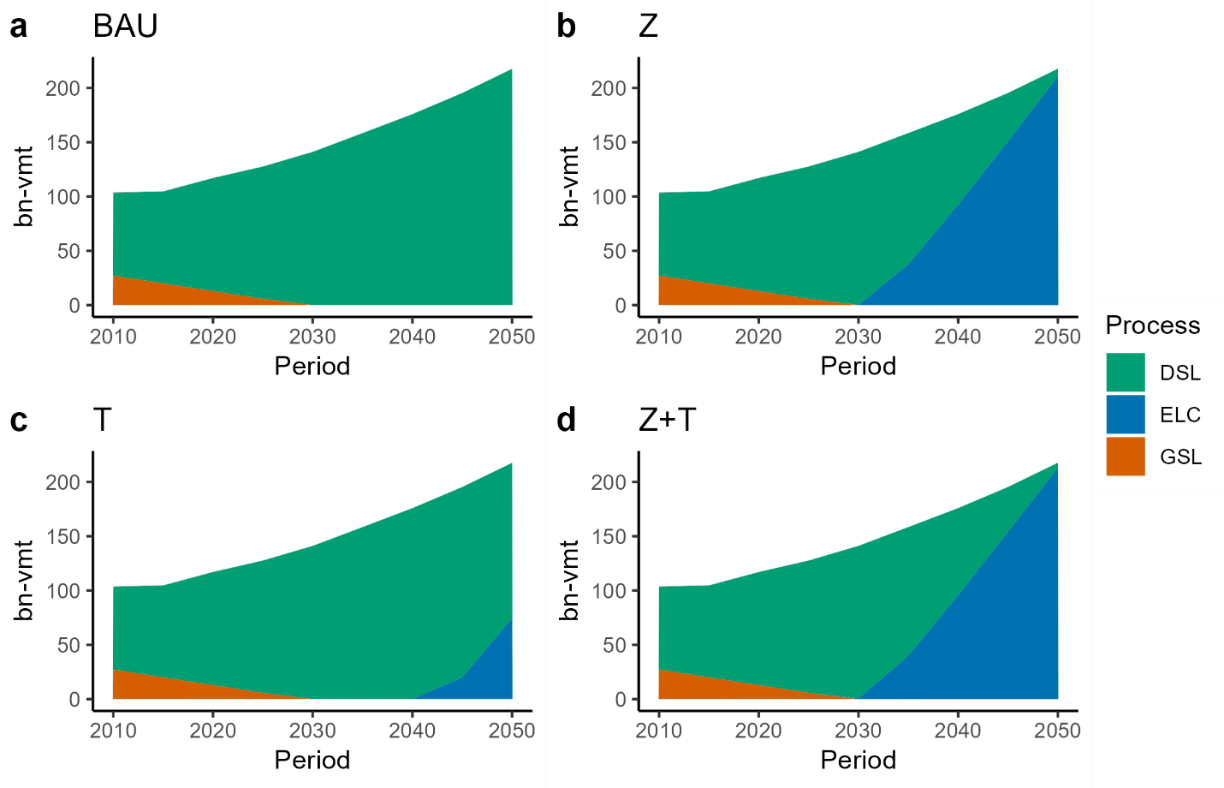


Figure S22. Medium-duty (“TM”) demand by technology in billion vehicle miles traveled. *DSL: diesel, ELC: battery electric, GSL: gasoline.* (NREL ATB Adv-Vehicle Scenario)

*
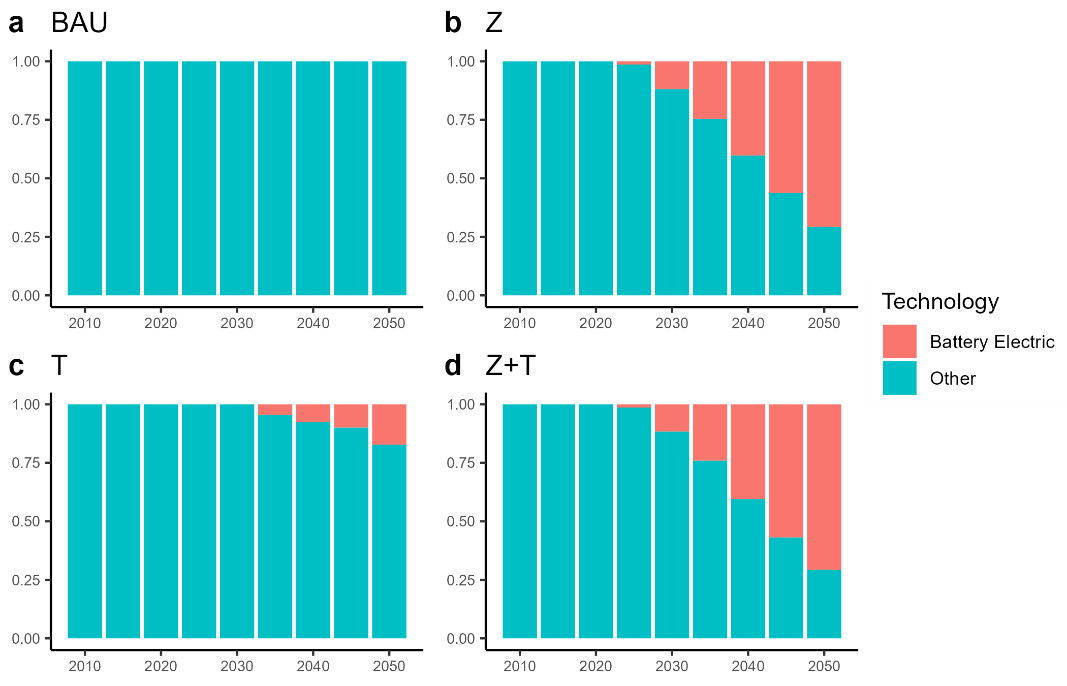
*

Figure S23. Fraction of MHDV demand met by battery electric vs other fuels (NREL ATB Adv-Vehicle Scenario)

**Table S13**: Air Quality NOx Budgets and resulting power sector NOx emissions, kT

|  | 2010 | 2015 | 2020 | 2025 | 2030 | 2035 | 2040 | 2045 | 2050 |
| --- | --- | --- | --- | --- | --- | --- | --- | --- | --- |
| CSAPR Limit on ELC NOX-> | *3317* | *3317* | *3317* | *1390.30* | *1341.37* | *1331.61* | *1321.86* | *1344.4* | *1366.94* |
| *Scenario* |  |  |  |  |  |  |  |  |  |
| reference | 311.83 | 256.26 | 260.27 | 105.77 | 102.51 | 102.51 | 103.28 | 103.65 | 104.52 |
| mou_only | 311.83 | 261.87 | 273.97 | 105.77 | 102.51 | 102.51 | 103.58 | 109.48 | 109.74 |
| tax2_w_mou | 311.83 | 254.77 | 249.26 | 137.47 | 135.87 | 136.04 | 136.69 | 146.10 | 146.38 |
| tax2_wo_mou | 311.83 | 254.76 | 249.35 | 137.66 | 136.06 | 136.06 | 136.21 | 143.89 | 147.26 |

**Table S14**: Air Quality SO2 Budgets and resulting power sector NOx emissions, kT

|  | 2010 | 2015 | 2020 | 2025 | 2030 | 2035 | 2040 | 2045 | 2050 |
| --- | --- | --- | --- | --- | --- | --- | --- | --- | --- |
| CSAPR Limit on ELC SO2-> | *8119* | *8119* | *8119* | *1363.5* | *1342.26* | *1320.77* | *1299.29* | *1308.99* | *1318.69* |
| *Scenario* |  |  |  |  |  |  |  |  |  |
| reference | 239.54 | 126.55 | 102.70 | 99.83 | 94.82 | 94.82 | 95.56 | 95.25 | 93.60 |
| mou_only | 239.54 | 126.78 | 103.23 | 99.83 | 94.82 | 94.82 | 95.31 | 95.13 | 94.26 |
| tax2_w_mou | 239.54 | 119.17 | 91.93 | 31.08 | 27.67 | 27.32 | 27.04 | 28.00 | 27.59 |
| tax2_wo_mou | 239.54 | 119.17 | 91.93 | 31.08 | 27.67 | 27.67 | 27.31 | 28.04 | 26.76 |

1. TIMES Documentation Part I. https://github.com/etsap-TIMES/TIMES_Documentation/blob/master/Documentation_for_the_TIMES_model-Part-I.docx [↑](#footnote-ref-2)
2. https://www.bpie.eu/wp-content/uploads/2015/10/Discount_rates_in_energy_system-discussion_paper_2015_ISI_BPIE.pdf [↑](#footnote-ref-3)
3. https://www.nrel.gov/docs/fy16osti/66944.pdf [↑](#footnote-ref-4)
4. Draft Memo from OnLocation, Inc to Brookhaven National Laboratory. Recommendation for establishing time slices in MARKAL modeling framework (2007). [↑](#footnote-ref-5)
5. U.S. Government Accountability Office. (2024) Electricity: Information on Peak Demand Power Plants GAO-24-106145

   Published: May 21, 2024. Publicly Released: May 21, 2024.https://www.gao.gov/assets/gao-24-106145.pdf [↑](#footnote-ref-6)
